# Trimeric and Dimeric Carbazole Alkaloids from *Murraya microphylla*

**DOI:** 10.3390/molecules26185689

**Published:** 2021-09-20

**Authors:** Xiaoli Ma, Hongwei Chen, Sisi Zhu, Pengfei Tu, Yong Jiang

**Affiliations:** State Key Laboratory of Natural and Biomimetic Drugs, School of Pharmaceutical Sciences, Peking University, No. 38 Xueyuan Road, Beijing 100191, China; love-maxiaoli@163.com (X.M.); chenhongwei@bjmu.edu.cn (H.C.); zsszmh@163.com (S.Z.); pengfeitu@bjmu.edu.cn (P.T.)

**Keywords:** *Murraya microphylla*, trimeric carbazole, dimeric carbazole, racemates, absolute configurations, ECD

## Abstract

Seventeen new carbazole alkaloid derivatives, including a trimeric carbazole racemate, (±)-microphyltrine A (**1**), 15 dimeric carbazole racemates, (±)-microphyldines A–O (**2**–**16**), and a C-6–C-3″-methyl-linked dimeric carbazole, microphyldine P (**17**), were isolated from the leaves and stems of *Murraya microphylla* (Merr. et Chun) Swingle. The structures of the new compounds were elucidated on the basis of HRESIMS and NMR data analysis. The optically pure isomers of these isolated carbazole alkaloids were obtained by chiral HPLC separation and their absolute configurations were determined by electronic circular dichroism (ECD) data analysis.

## 1. Introduction

Carbazole alkaloids, one type of bioactive constituents from the *Murraya* genus, have been demonstrated to possess anti-inflammatory, antitumor, antimicrobial, antioxidant, and antidiabetic properties [1]. Many of the biologically active carbazole alkaloids have been isolated from four closely related genera, *Clausena*, *Glycosmis*, *Murraya*, and *Micromelum* of the family Rutaceae [2,3,4]. *Murraya microphylla* (Merr. et Chun) Swing (*M. microphylla*) is a shrub distributed in the thickets of sandy areas or coastal regions in the Hainan Province of China [5]. Previous chemical investigations have confirmed that *M. microphylla* contains abundant carbazole alkaloids [6,7].

**Figure 1:**
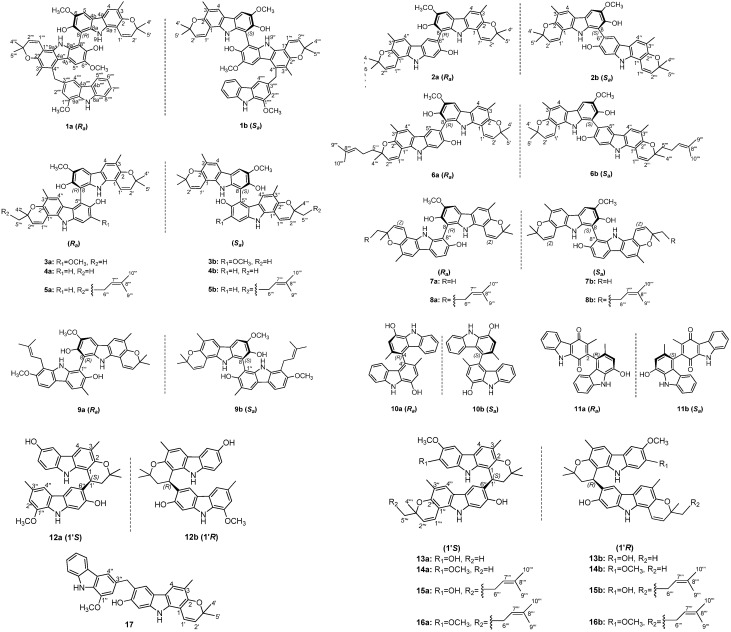
Structures of compounds **1**–**17**.

In the course of the search for bioactive carbazole alkaloids from *Murraya* species, the 95% aqueous ethanol extract of the leaves and stems of *M. microphylla* was investigated and 17 new carbazole alkaloid derivatives, namely (±)-microphyltrine A (**1**), (±)-microphyldines A–O (**2**–**16**), and microphyldine P (**17**) (Figure 1) were obtained. (±)-Microphyltrine A (**1**) is an unprecedented racemate of trimeric carbazole atropisomers. Microphyldines A–P (**2**–**17**) are new carbazole dimers, among which, (±)-microphyldines A–J (**2**–**11**) are 10 biphenyl-type carbazole dimeric racemates, while (±)-microphyldines K–O (**12**–**16**) are five pyranocarbazole dimeric racemates, and microphyldine P (**17**) is a C-6–C-3″-methyl-linked dimeric carbazole alkaloid. The optically pure isomers of these isolated carbazole racemates were obtained by chiral HPLC separation. Herein, we describe the isolation and structural characterization of compounds **1**–**17**, as well as their activity screening on lipopolysaccharide (LPS)-induced nitric oxide (NO) production in BV-2 microglial cells and murine monocytic RAW 264.7 macrophages and cytotoxicities on HepG2, Du145, HCT116, and HeLa cells.

## 2. Results

### 2.1. Structural Elucidation

**Figure 2:**
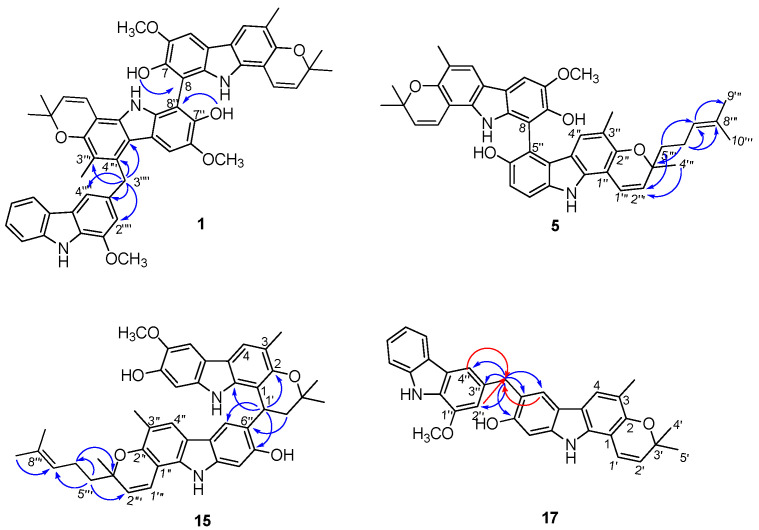
Key HMBC correlations of compounds **1**, **5**, **15**, and **17**.

(±)-Microphyltrine A (**1**) was obtained as a white amorphous powder. Its molecular formula was determined as C_52_H_47_N_3_O_7_ based on the HRESIMS (*m*/*z* 824.3330 [M − H]^−^, calcd. for C_52_H_46_N_3_O_7_, 824.3336) and ^13^C NMR data. The UV spectrum showed absorptions at 227, 252, 293, and 332 nm, suggesting the presence of a pyranocarbazole skeleton in the molecule [8,9,10]. Analysis of the ^1^H NMR data (Table 1) revealed the presence of five labile proton signals (*δ*_H_ 7.05 (1H, br s, 7″-OH), 7.16 (1H, br s, 7-OH), 9.55 (1H, br s, 9″-NH), 9.58 (1H, br s, 9-NH), 10.25 (1H, br s, 9⁗-NH)), five aromatic doublets and singlets (*δ*_H_ 7.15 (1H, d, *J* = 2.0 Hz, H-2⁗), 7.56 (1H, d, *J* = 2.0 Hz, H-4⁗), 7.59 (1H, s, H-5″), 7.60 (1H, s, H-5), 7.62 (1H, s, H-4)), an ortho-substituted phenyl moiety (*δ*_H_ 7.10 (1H, t, *J* = 8.0 Hz, H-6⁗), 7.34 (1H, t, *J* = 8.0 Hz, H-7⁗), 7.55 (1H, d, *J* = 8.0 Hz, H-8⁗), 7.91 (1H, d, *J* = 8.0 Hz, H-5⁗)), three methoxy groups (*δ*_H_ 3.82 (3H, s, 6″-OCH_3_), 3.98 (3H, s, 1⁗-OCH_3_), 4.01 (3H, s, 6-OCH_3_)), two methyl groups (*δ*_H_ 2.28 (3H, s, 3-CH_3_) 2.42 (3H, s, 3″-CH_3_)), a methylene singlet (*δ*_H_ 4.84 (2H, s, 3⁗-CH_2_)), and two 2,2-dimethyl-2*H*-pyran moieties (*δ*_H_ 1.37/1.45 (3H, s, H-5‴/H-5′), 1.38/1.49 (3H, s, H-4‴/H-4′), 5.48/5.59 (1H, d, *J* = 10 Hz, H-2‴/H-2′), 6.66/6.81 (1H, d, *J* = 10 Hz, H-1‴/H-1′)). In the ^13^C NMR data of **1**, there were 52 carbon resonances, comprising 42 olefinic, six methyl, three methoxy, and one methylene carbons. These data suggested **1** to be a carbazole trimer, consisting of three pyranocarbazole units similar to two koenigine [11,12] and a murrayafoline A moiety [13], in the combination of the HSQC and HMBC spectral analysis. The murrayafoline A unit was deduced to be connected to the middle koenigine unit via a C-4″–C-3⁗-methyl-linked mode, on the basis of HMBC correlations from 3⁗-CH_2_ to C-3″, C-4″, C-4a″, C-2⁗, C-3⁗, and C-4⁗, and the reverse correlations of H-2⁗/4⁗ to C-3⁗. The deficiency of H-8 and H-8″, together with the HMBC correlations from 7-OH to C-8 and from 7″-OH to C-8″, implied the two koenigine units were linked by a C-8–C-8″ bond (Figure 2). Thus, the planar structure of microphyltrine A was proposed as shown (Figure 1).

**Figure 3:**
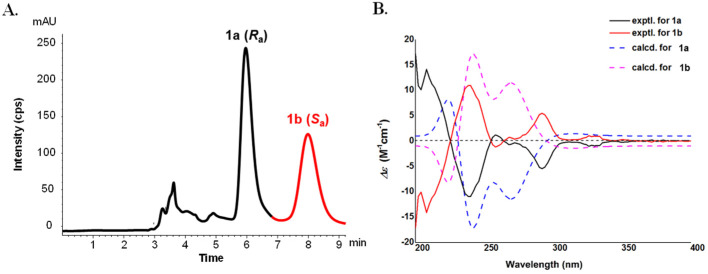
The chiral HPLC separation (**A**) and experimental and calculated ECD data (**B**) of compounds **1a** and **1b**.

Considering the high steric hindrance at the central biaryl axis of C-8–C-8″, **1** was supposed to be with an axial chirality. However, its specific rotation value approached zero and no Cotton effects were observed in its ECD spectrum, suggesting **1** to be a pair of atropisomers coexisting as a racemic mixture. Compound **1** was isolated by a chiral HPLC with *n*-hexane-isopropanol (85:15, *v*/*v*, 1 mL/min) as the mobile phase to give the enantiomers **1a** and **1b** in an approximate ratio of 1:1. Their specific rotations were detected as ${\left[\alpha \right]}_{D}^{25}$ −74 (*c* 0.1, MeOH) for **1a** and ${\left[\alpha \right]}_{D}^{25}$ +84 (*c* 0.1, MeOH) for **1b**, respectively. The ECD spectrum of **1a** exhibited a sequential negative and positive Cotton effect at 245 nm and 218 nm, i.e., a negative couplet derived from the electronic transition of the two carbazole monomers, indicating an (*R_a_*) configuration for (−)-microphyltrine A (**1a**) and, accordingly, the configuration of (+)-microphyltrine A (**1b**) was defined as (*S_a_*) [14]. Furthermore, the ECD spectra of (*R_a_*)- and (*S_a_*)-**1** were calculated using the TDDFT method at the B3LYP/6-311G(d) level to confirm the results of ECD exciton coupling. The computed ECD spectrum of (*R_a_*)-**1** matched well with the experimental curve for **1a** and the computed ECD spectrum of (*S_a_*)-**1** matched well with the experimental curve for **1b** (Figure 3). This is the second report for trimeric carbazole alkaloids from a natural source, for the first was murratrines A and B from *M. tetramera* [4], whose units are three simple carbazoles linked with a bismethylene ether and a C-3-methyl-linked mode.

(±)-Microphyldine A (**2**) was obtained as a brown amorphous powder, and its molecular formula was determined as C_37_H_34_N_2_O_5_ from the ^13^C NMR spectroscopic data and the HRESIMS ion at *m*/*z* 585.2387 [M − H]^−^ (calcd. for C_37_H_33_N_2_O_5_, 585.2389). Four labile proton signals (*δ*_H_ 7.43 (1H, br s, 7-OH), 7.43 (1H, br s, 7″-OH), 9.58 (1H, br s, 9-NH), 10.14 (1H, br s, 9″-NH)), five aromatic singlet protons (*δ*_H_ 7.04 (1H, s, H-8″), 7.61 (1H, s, H-5), 7.63 (1H, s, H-4″), 7.65 (1H, s, H-4), 7.87 (1H, s, H-5″)), a methoxy group (*δ*_H_ 4.02 (3H, s, 6-OCH_3_)), two methyl groups (*δ*_H_ 2.29 (3H, s, 3″-CH_3_) 2.31 (3H, s, 3-CH_3_)), and two 2,2-dimethyl-2*H*-pyran moieties (*δ*_H_ 1.42/1.49 (each 3H, s, H-5′/H-5‴), 1.43/1.50 (each 3H, s, H-4′/H-4‴), 5.58/5.80 (each 1H, d, *J* = 10 Hz, H-2′/H-2‴), 6.87/6.94 (each 1H, d, *J* = 10 Hz, H-1′/H-1‴)) were observed in the ^1^H NMR data (Table 2).

The ^13^C NMR data (Table 3) showed 37 carbon resonances, comprising 30 olefinic, six methyl, and one methoxy carbons. The above data, coupled with information from the literature [4,14,15] and 2D NMR analysis, indicated a dimeric carbazole skeleton of **2**, and the two carbazole units were deduced as koenigine [12] and murrayamine A [16] moieties, respectively. The HMBC correlations from H-5″ to C-8 indicated that the two units were linked by a C-8–C-6″ bond (Figure S12, Supporting Information). Therefore, the planar structure of **2** was assigned as shown (Figure 1). 

**Figure 4:**
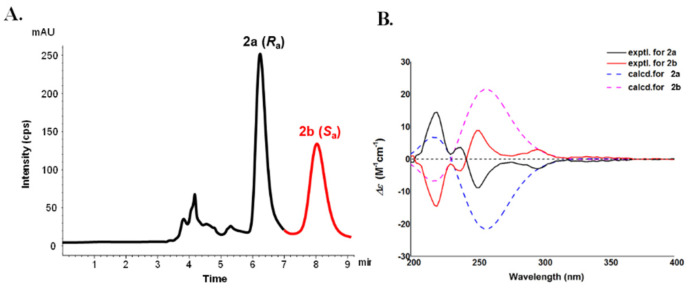
The chiral HPLC separation (**A**) and experimental and calculated ECD data (**B**) of compounds **2a** and **2b**.

Compound **2** could also be a racemate owing to the disappeared specific rotation and Cotton effects in the ECD spectrum, thus, it was then separated by a chiral HPLC to give the enantiomers **2a** and **2b** for almost equal quantity, which possess the opposite ECD curves and specific rotations. Similar to **1**, the ECD spectrum of **2a** exhibited sequential negative and positive Cotton effects at 252 nm and 224 nm, indicating an (*R_a_*) configuration for (−)-microphyldine A (**2a**) [14] and, accordingly, the configuration of (+)-microphyldine A (**2b**) was defined as (*S_a_*). Furthermore, the ECD spectra of (*R_a_*)- and (*S_a_*)-**2** were calculated and compared with the experimental spectra to support the results of ECD exciton coupling (Figure 4).

(±)-Microphyldine B (**3**) gave a molecular formula of C_38_H_36_N_2_O_6_, as established by ^13^C NMR data and an [M − H]^−^ ion at *m*/*z* 615.2486 [M − H]^−^(calcd for C_38_H_35_N_2_O_6_, 615.2495) in the HRESIMS. The ^1^H and ^13^C NMR data (Table 2 and Table 3) of **3** showed close resemblance to those of **2**, except that one of the phenyl singlets in **3** was missing, but an additional methoxy signal was observed at *δ*_H_ 3.99 (3H, s). After 2D NMR analysis (Figure S19, Supporting Information), the two units were deduced to be both koenigine units [12]. The deficiency of H-8 and H-5″ signals suggested **3** to be a C-8–C-5″-linked dimeric carbazole. Thus, the planar structure of **3** was assigned as shown (Figure 1). 

**Figure 5:**
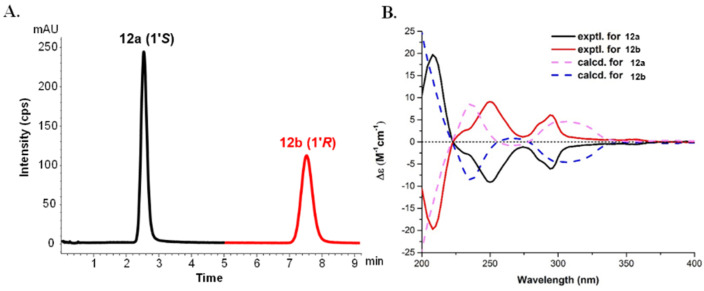
The chiral HPLC separation (**A**) and experimental and calculated ECD data (**B**) of compounds **12a** and **12b**.

Similar to **2**, compound **3** is also an atropisomeric racemate, and the following chiral HPLC resolution afforded **3a** and **3b** in a ratio of 1:1, and their absolute configurations were established as (*R_a_*) and (*S_a_*), respectively, by comparison of their ECD (Figure S18, Supporting Information) and specific rotations with those of **2a** and **2b** [14].

(±)-Microphyldine C (**4**) exhibited an [M − H]^−^ ion at *m*/*z* 585.2389 in the HRESIMS, which, in conjunction with the ^13^C NMR data, suggested a molecular formula of C_37_H_34_N_2_O_5_ (calcd. for C_37_H_33_N_2_O_5_, 585.2389). Analysis of 1D and 2D NMR data (Table 2 and Table 3) suggested that the structure of **4** resembled that of **3**, except for the disappearance of one methoxy group in **4**, and the replacement of an aromatic singlet in **3** by two aromatic doublets (*δ*_H_ 7.00 (1H, d, *J* = 8.0 Hz, H-7″), 7.31 (1H, d, *J* = 8.0 Hz, H-8″)). This suggested that **4** is the demethoxy derivative of **3**. Further 2D NMR analysis (Figure S26, Supporting Information) deduced the structure of **4** as shown (Figure 1). The isolation of individual enantiomers (**4a** and **4b**) was accomplished by a chiral HPLC separation and their absolute configurations were established as (*R_a_*) and (*S_a_*), respectively, by comparison of their ECD (Figure S25, Supporting Information) and specific rotations with those of **2a** and **2b** [14].

(±)-Microphyldine D (**5**) was isolated as an amorphous powder. Its ^13^C NMR and negative-ion HRESIMS data at *m*/*z* 653.3023 [M − H]^−^ (calcd for C_42_H_41_N_2_O_5_, 653.3015) established a molecular formula of C_42_H_42_N_2_O_5_. The ^1^H and ^13^C NMR data (Table 2 and Table 3) of **5** were comparable to those of **4**, except for the presence of a set of additional resonances for isopentenyl group (*δ*_H_ 1.52 (3H, s, H-10‴), 1.61 (3H, s, H-9‴), 2.11 (2H, m, H-6‴), 5.08 (1H, t, *J* = 6.0 Hz, H-7‴), *δ*_C_ 16.2 (C-10‴), 22.1 (C-6‴), 24.7 (C-9‴), 123.8 (C-7‴), 130.4 (C-8‴)) in **5**. The HMBC correlations from H-5‴ to C-2‴ and C-7‴ and from H-6‴ to C-3‴, C-7‴, and C-8‴ suggested that the isopentenyl moiety is located at C-5‴ (Figure 2). Compound **5** is also a pair of atropisomers, and the following chiral HPLC resolution afforded **5a** and **5b** in a ratio of 1:1, and their absolute configurations were established as (*R_a_*) and (*S_a_*), respectively, by comparison of their ECD (Figure S32, Supporting Information) and specific rotations with those of **4a** and **4b** [14]. The experimental ECD spectra of **5a**/**5b** are almost similar to those of **4a**/**4b**, indicating that the C-3″′configuration did not have much influence on the ECD curves, which was proofed by the calculated ECD data of the different configurations of C-3‴. Thus, the C-3‴configuration was undetermined in this paper. 

(±)-Microphyldine E (**6**) was obtained as an amorphous powder with a molecular formula of C_42_H_42_N_2_O_5_, as deduced from the ^13^C NMR and HRESIMS (*m*/*z* 653.3023 [M − H]^−^ (calcd for C_42_H_41_N_2_O_5_, 653.3015) data. Its ^1^H and ^13^C NMR (Table 2 and Table 3) data showed many similarities to those of **2**, except for the presence of a set of additional resonances for isopentenyl group (*δ*_H_ 1.59 (3H, s, H-10‴), 1.66 (3H, s, H-9‴), 2.23 (2H, m, H-6‴), 5.16 (1H, t, *J* = 6.0 Hz, H-7‴), *δ*_C_ 16.7 (C-10‴), 22.6 (C-6‴), 24.9 (C-9‴), 124.3 (C-7‴), 131.0 (C-8‴)) in **6**. The HMBC correlations from H-5‴ to C-2‴ and C-7‴ and from H-6‴ to C-3‴, C-7‴, and C-8‴ suggested that the isopentenyl moiety is located at C-5‴ (Figure S39, Supporting Information). Compound **6** was separated by a chiral HPLC to afford the enantiomers **6a** and **6b**. The ECD spectra of **6a/6b** were calculated using the TDDFT method at the B3LYP/6-311G(d) level to determine the absolute configuration. The computed ECD spectrum of (*R_a_*)-**6** matched the experimental curve for **6a**. and the computed ECD spectrum of (*S_a_*)-**6** matched the experimental curve for **6b**. Thus, the absolute configuration of **6a** was defined as (*R_a_*) and, accordingly, **6b** was defined as (*S_a_*) (Figure S38, Supporting Information).

(±)-Microphyldine F (**7**) was shown to have the same molecular formula as **2**, according to its ^13^C NMR data and the [M − H]^−^ ion at *m*/*z* 585.2390 in the HRESIMS (calcd. for C_37_H_33_N_2_O_5_, 585.2389). Analysis of ^1^H and ^13^C NMR data (Table 2 and Table 3) of **7** showed a close structural resemblance to **2**, a dimeric carbazole formed by koenigine [12] and murrayamine A [16] units. The difference between them is that the linkage mode is shifted from C-8–C-6″ in **2** to C-8–C-8″ in **7**, as deduced from the deficiency of H-8 and H-8″ signals in **7**. Accordingly, the structure of **7** was determined as shown (Figure 1). A subsequent chiral HPLC isolation was performed to obtain the pure enantiomers of **7a** and **7b** in a ratio of 1:1. After ECD and specific rotation determination, the absolute configurations of **7a** and **7b** were established as (*R_a_*) and (*S_a_*), respectively, by comparison of their ECD (Figure S45, Supporting Information) and specific rotations with those of **2a** and **2b** [14].

(±)-Microphyldine G (**8**) was isolated as an amorphous powder. The HRESIMS gave a deprotonated molecular ion at *m*/*z* 653.3007 [M − H]^−^ (calcd for C_42_H_41_N_2_O_5_, 653.3015), corresponding to a molecular formula of C_42_H_42_N_2_O_5_. The ^1^H and ^13^C NMR data (Table 2 and Table 3) of **8** were found to be similar to those of **7**. Their apparent difference was the presence of a set of resonances for isopentenyl group (*δ*_H_ 1.55 (3H, s, H-10‴), 1.63 (3H, s, H-9‴), 2.15 (2H, m, H-6‴), 5.11 (1H, t, *J* = 6.0 Hz, H-7‴), *δ*_C_ 16.7 (C-10‴), 22.6 (C-6‴), 24.9 (C-9‴), 124.3 (C-7‴), 130.9 (C-8‴)) in **8**. The HMBC correlations from H-5‴ to C-2‴ and C-7‴ and from H-6‴ to C-3‴, C-7‴, and C-8‴ suggested that the isopentenyl moiety is located at C-5‴ (Figure S53, Supporting Information). Thus, the planar structure of **8** was assigned as shown (Figure 1). Compound **8** was also a pair of atropisomer mixtures and was then separated by a chiral HPLC to give the enantiomers **8a** and **8b**. The ECD spectra of **8a/8b** were calculated using the TDDFT method at the B3LYP/6-311G(d) level to determine the absolute configuration. The computed ECD spectrum of (*R_a_*)-**8** matched the experimental curve for **8a**. and the computed ECD spectrum of (*S_a_*)-**8** matched the experimental curve for **8b.** Thus, the absolute configuration of **8a** was defined as (*R_a_*) and accordingly, **8b** was defined as (*S_a_*). (Figure S52, Supporting Information).

(±)-Microphyldine H (**9**) was obtained as a brown amorphous powder with a molecular formula of C_38_H_38_N_2_O_5_, as deduced from the ^13^C NMR and HRESIMS data (*m*/*z* 601.2692 [M − H]^−^, calcd for C_38_H_37_N_2_O_5_, 601.2702). The ^1^H and ^13^C NMR data (Table 2 and Table 3) analysis revealed that the structure of **9** is a dimeric carbazole formed by a koenigine [12] and an isomurrayafoline B [17,18] unit. The 2D NMR data (Figure S60, Supporting Information) analysis indicated the linkage mode of **9** was C-8–C-1″ due to the absence of H-8 and H-1″ protons. Accordingly, the structure of **9** was determined as shown (Figure 1). A subsequent chiral HPLC isolation was performed to obtain the pure enantiomers of **9a** and **9b** in a ratio of 1:1. After ECD and specific rotation determination, the absolute configurations of **9a** and **9b** were established as (*R_a_*) and (*S_a_*), respectively, by comparison of their ECD (Figure S59, Supporting Information) and specific rotations with those of **2a** and **2b** [14].

(±)-Microphyldine I (**10**) was isolated as a brown amorphous powder. Its molecular formula was defined as C_26_H_20_N_2_O_2_ via its ^13^C NMR and HRESIMS data (*m*/*z* 391.1440 [M − H]^−^, calcd for C_26_H_19_N_2_O_2_, 391.1447). The ^13^C NMR data of **10** exhibited only 13 carbon signals, suggesting that it is a symmetrical carbazole dimer. The NMR data (Table 2 and Table 3) of the monomeric unit of **10** resembled those of 1-hydroxy-3-methylcarbazole [19], except for the absence of H-4 proton and a shift of the C-4 signal downfield to *δ*_C_ 125.0, indicating that the two units are linked through C-4–C-4′. Compound **10** was a pair of atropisomer mixtures inferred from its almost zero specific rotation and weak ECD Cotton effects. The pure enantiomers of **10a** and **10b** were obtained by a chiral HPLC separation. The absolute configurations of **10a** and **10b** were defined as (*R_a_*) and (*S_a_*), respectively, from their experimental ECD spectra and computed ECD spectra using the TDDFT method at the B3LYP/6-311 + G(d) level (Figure S66, Supporting Information).

(±)-Microphyldine J (**11**) was obtained as an amorphous powder. It has a molecular formula of C_26_H_18_N_2_O_3_ determined by the HRESIMS data showing a deprotonated molecular ion at *m*/*z* 405.1232 [M − H]^−^ (calcd for C_26_H_17_N_2_O_3_, 405.1239) and its ^13^C NMR data. Its 1D and 2D NMR data (Table 2 and Table 3) showed many similarities to those of murrayaquinone A [20], except for the replacement of a methoxy singlet in murrayaquinone A by a hydroxy singlet (*δ*_H_ 8.80 (1H, br s)) in **11**. The 2D NMR analysis, especially of HMBC correlation from OH-1′ to C-9a, from CH_3_-3 to C-2, and from CH_3_-3′ to C-4′ proved the above deduction (Figure S74, Supporting Information). Compound **11** was separated by a chiral HPLC to give the enantiomers of **11a** and **11b**. The absolute configurations of **11a** and **11b** were defined as (*R_a_*) and (*S_a_*), respectively, from their experimental ECD and computed ECD spectra (Figure S73, Supporting Information). 

(±)-Microphyldine K (**12**) was obtained as a brown amorphous powder with a molecular formula of C_32_H_30_N_2_O_4_ based on the HRESIMS (*m*/*z* 505.2125 [M − H]^−^, calcd. for C_32_H_29_N_2_O_4_, 505.2127) and ^13^C NMR data. The ^1^H NMR data (Table 4) of **12** were found to be similar to those of murrafoline D [21]. The apparent differences were the replacement of two aromatic signals in murrafoline D by two active hydrogen signals in **12**, suggesting that **12** is a dihydroxy derivative of murrafoline D [21]. The two hydroxy groups were deduced to be located at C-6 and C-7″, respectively, via the HMBC correlation of one hydroxy proton (*δ*_H_ 7.66 (1H, br s)) with C-5/C-7, and the other hydroxy proton (*δ*_H_ 8.23 (1H, br s) with C-6″ and C-8″ (Figure S80, Supporting Information). The optical inactivity of **12** indicated that it is a pair of enantiomer mixture, thus a chiral HPLC isolation was performed to obtain the pure enantiomers of **12a** and **12b**. The ECD spectra of **12a**/**12b** were calculated using the TDDFT method at the B3LYP/6-311G(d) level to determine the absolute configuration. The computed ECD spectrum of (1′*S*)-**12a** matched the experimental curve for **12a** (Figure 5). Thus, the absolute configuration of **12a** was defined as (1′*S*) and, accordingly, **12b** was defined as (1′*R*).

(±)-Microphyldine L (**13**) gave a molecular formula of C_37_H_36_N_2_O_5_ on the basis of its ^13^C NMR and HRESIMS (*m*/*z* 587.2543 [M − H]^−^, calcd. for C_37_H_35_N_2_O_5_, 587.2546). The UV and NMR data (Table 4) of **13** were closely comparable to those of microphyldine K (**12**), indicating that it also has a biscarbazole skeleton like **12**. The difference between them is that **13** is a dimeric carbazole formed by koenigine [12] and murrayamine A [16] units, which were further deduced from the 2D NMR data. The linkage of these two units was determined to be via the C-1′-C-6″ bond, supported by the HMBC correlations of H-2′ and C-6″, and H-1′ and C-5″/C-7″ (Figure S87, Supporting Information). Accordingly, the structure of **13** was determined as shown (Figure 1). A subsequent chiral HPLC isolation was performed to obtain the pure enantiomers of **13a** and **13b** in a ratio of 1:1. The absolute configurations of **13a** and **13b** were defined as (1′*S*) and (1′*R*), respectively, by comparison of the experimental and calculated ECD spectra (Figure S86, Supporting Information).

(±)-Microphyldine M (**14**) gave a molecular formula of C_38_H_38_N_2_O_5_ based on the ^13^C NMR and a deprotonated ion at *m*/*z* 601.2712 [M − H]^−^ (calcd. for C_38_H_37_N_2_O_5_, 601.2702) in the negative-ion HRESIMS. Its 1D (Table 4) and 2D NMR data showed many similarities to those of **13**, except for the replacement of a hydroxy singlet (*δ*_H_ 7.14 (1H, br s)) in **13** by a methoxy singlet (*δ*_H_ 3.64 (3H, s)) in **14**. This suggested that **14** is a 7-methoxy derivative of **13**, as deduced from the HMBC correlation of the methoxy protons with C-7 (*δ*_C_ 149.6) (Figure S94, Supporting Information). Hence, the structure of **14** was defined as shown (Figure 1). The pure enantiomers of **14a** and **14b** were obtained by a chiral HPLC separation. A comparison of the experimental and calculated ECD spectra facilitated the assignment of the absolute configurations of **14a** and **14b** as (1′*S*) and (1′*R*), respectively (Figure S93, Supporting Information).

(±)-Microphyldine N (**15**) gave a molecular formula of C_42_H_44_N_2_O_5_, as determined from its ^13^C NMR and HRESIMS data (*m*/*z* 655.3156 [M − H]^−^, calcd. for C_42_H_43_N_2_O_5_, 655.3172). Its 1D (Table 4) and 2D NMR data showed many similarities to those of **13** and the apparent difference was the presence of a set of resonances for the isopentenyl group (*δ*_H_ 1.54 (3H, s, H-10‴), 1.62 (3H, s, H-9‴), 2.15 (2H, m, H-6‴), 5.11 (1H, t, *J* = 6.0 Hz, H-7‴), *δ*_C_ 18.1 (C-10‴), 23.9 (C-6‴), 26.2 (C-9‴), 125.7 (C-7‴), 132.3 (C-8‴)) in **15**. The HMBC correlations from H-5‴ to C-2‴, C-6‴, and C-7‴ and from H-6‴ to C-3‴ and C-5‴ suggested that the isopentenyl moiety is located at C-5‴(Figure 2). Hence, the planar structure of **15** was assigned as shown (Figure 1). The following chiral HPLC resolution and ECD determination defined the absolute configurations of **15a** and **15b** as (1′*S*) and (1′*R*), respectively (Figure S100, Supporting Information).

(±)-Microphyldine O (**16**) was isolated as an amorphous powder with a molecular formula of C_43_H_46_N_2_O_5_, as deduced from the ^13^C NMR and HRESIMS data (*m*/*z* 669.3328 [M − H]^−^, calcd for C_43_H_45_N_2_O_5_, 669.3328). The ^1^H and ^13^C NMR data (Table 4) of **16** were found to be similar to those of **14**. The obvious difference was the presence of a set of resonances for the isopentenyl group (*δ*_H_ 1.54 (3H, s, H-10‴), 1.61 (3H, s, H-9‴), 2.15 (2H, m, H-6‴), 5.11 (1H, t, *J* = 6.0 Hz, H-7‴), *δ*_C_ 16.2 (C-10‴), 22.1 (C-6‴), 24.7 (C-9‴), 123.8 (C-7‴), 130.4 (C-8‴)) in **16**. The HMBC correlations from H-5‴ to C-6‴ and C-7‴ and from H-6‴ to C-3‴ and C-5‴ suggested that the isopentenyl moiety is located at C-5‴ (Figure S107, Supporting Information). Thus, the planar structure of **16** was assigned as shown (Figure 1). Compound **16** is also a racemate and was then separated by a chiral HPLC to give the enantiomers of **16a** and **16b**. By comparison of the experimental and calculated ECD spectra, the absolute configurations of **16a** and **16b** were established as (1′*S*) and (1′*R*), respectively (Figure S106, Supporting Information).

Microphyldine P (**17**) was obtained as an amorphous powder. Its molecular formula was determined as C_32_H_28_N_2_O_3_ base on its ^13^C NMR and HRESIMS data (*m*/*z* 487.2016 [M − H]^−^, calcd for C_32_H_27_N_2_O_3_, 487.2022). The ^1^H and ^13^C NMR data (Table 2 and Table 3) analysis revealed that the structure of **17** is a dimeric carbazole formed by murrayamine A [16] and murrayafoline A [22,23] units. Additionally, the HMBC correlations from the 3″-CH_2_ protons to C-5/C-6/C-7/C-2″/C-3″/C-4″, and H-5/H-2″/H-4″ to 3″-CH_2_ revealed a C-6–C-3″-methyl linkage between the two carbazole moieties (Figure 2). Thus, the structure of microphyldine P (**17**) was defined as shown (Figure 1).

### 2.2. Bioactivity

All of these isolates (**1**–**17**) were subjected to an evaluation of their inhibition on nitric oxide (NO) production stimulated by lipopolysaccharide (LPS) in BV-2 microglial cells and RAW 264.7 macrophages in reference to the traditional anti-inflammation use of *Murraya* genera [24,25]. Moreover, we tested their cytotoxic activities on HepG2, Du145, HCT116, and HeLa cells. However, none of these isolates displayed significant NO inhibitory or cytotoxic activities at 50 μM. Further testing of the biological activities of these isolates is under progress.

## 3. Discussion

Seventeen previously undescribed carbazole alkaloids were isolated and identified from the 95% aqueous EtOH extract of *M. microphylla*. (±)-Microphyltrine A (**1)** is a racemate of a pair of novel trimeric carbazole atropisomers from a natural source. (±)-Microphyldines A–P (**2**–**17**) are new carbazole dimeric racemates, among which, (±)-microphyldines A–J (**2**–**11**) are 10 pairs of biphenyl-type carbazole atropisomers, and (±)-microphyldines K–O (**12**–**16**) are five pairs of C1′−C6″-linked pyranocarbazole dimeric enantiomers. The chirally pure isomers of carbazole alkaloids (**1a/1b**–**16a/16b**) were obtained by chiral HPLC separation, and their absolute configurations were determined by electronic circular dichroism (ECD) data analysis. Unfortunately, none of the isolated alkaloids demonstrated significant NO inhibition or cytotoxicity at 50 μM.

## 4. Materials and Methods

### 4.1. General Experimental Procedures

UV spectra were recorded on a Shimadzu UV-2450 spectrophotometer (Shimadzu Co., Tokyo, Japan). Optical rotations were measured on a Rudolph Autopol IV automatic polarimeter (NJ, USA). ECD data were acquired on a J-810 CD spectrophotometer (JASCO, Japan). IR spectra were recorded on a Thermo Nicolet Nexus 470 FT-IR spectrometer (MA, USA). The NMR spectra were measured with a Bruker Plus-400 NMR spectrometer (Bruker Co., Switzerland) or a Varian INOVA-500 NMR spectrometer (Varian Co., USA), using acetone-*d*_6_ or CDCl_3_ as solvent, and the chemical shifts were referenced to the solvent residual peak. HRESIMS experiments were measured on a Waters Xevo G2 Q-TOF mass spectrometer (Waters Co., Milford, MA, USA). Column chromatography (CC) was performed on silica gel (100−200 mesh or 200−300 mesh, Qingdao Marine Chemical Co. Ltd., Qingdao, China). Semipreparative HPLC was carried out using a ZORBAX Eclipse XDB-C18 column (10 mm × 250 mm, 5 μm) on an Agilent 1200 series LC instrument with a DAD detector (Agilent Technologies, Palo Alto, CA, USA). Preparative TLC and TLC analyses were carried out on the pre-coated silica gel GF254 plates (Qingdao Marine Chemical Co. Ltd., Qingdao, China). Spots were visualized under the UV lights (254 and 365 nm) or by heating after spraying with 2% vanillin-H_2_SO_4_ solution. All the solvents used for isolation were of analytical grade and the solvents used for HPLC were of HPLC grade.

### 4.2. Plant Material

The dry leaves and stems of *Murraya microphylla* were collected from the Hainan Province, People’s Republic of China, in July 2015. The plant material was identified by one of the authors (P.F. Tu). A voucher specimen (no. MM201507) has been deposited at the Herbarium of the Peking University Modern Research Center for Traditional Chinese Medicine.

### 4.3. Extraction and Isolation

Air-dried and finely powdered leaves and stems of *M. microphylla* (16 kg) were refluxed three times with 95% aqueous EtOH (160 L × 2 h) and concentrated under reduced pressure to obtain a dry extract (730 g). The extract was suspended in H_2_O and extracted with CH_2_Cl_2_ and *n*-BuOH, successively. The CH_2_Cl_2_ extract (410 g) was subjected to a silica gel column and eluted with a stepwise gradient of petroleum ether–acetone (98:2, 96:4, 90:10, 80:20, 60:40, and 40:60, *v*/*v*) to obtain eight fractions (A−H). Fraction F (8.0 g) was subjected to a Sephadex LH-20 column eluted with MeOH–CH_2_Cl_2_ (1:1, *v*/*v*) and produced three subfractions (F1–F3). Subfraction F2 (2.2 g) was separated into three fractions (F2a–F2c) using an ODS column eluted with a stepwise gradient of MeOH–H_2_O (40:60, 50:50, 70:30, and 100:0, *v*/*v*). Fraction F2b (230 mg) was purified by semipreparative HPLC (3.0 mL/min, 0–25 min MeCN/H_2_O (75:25)) to yield **1** (8.0 mg, *t*_R_ 11.8 min), **5** (11.0 mg, *t*_R_ 14.4 min), **7** (5.0 mg, *t*_R_ 17.9 min), and **12** (4.0 mg, *t*_R_ 20.1 min). Fraction G (12.0 g) was subjected to a Sephadex LH-20 column eluted with MeOH–CH_2_Cl_2_ (1:1, *v*/*v*) and produced three subfractions (G1–G3). Fraction G2 (132 mg) was purified by semipreparative HPLC (3.0 mL/min, 0–25 min MeCN/H_2_O (60:40)) to yield **2** (6.0 mg, *t*_R_ 13.4 min), **3** (5.0 mg, *t*_R_ 15.4 min), and **4** (4.0 mg, *t*_R_ 19.1 min). Subfraction G3 (4.2 g) was separated into five fractions (G3a–G3e) using an ODS column eluted with a stepwise gradient of MeOH–H_2_O (40:60, 60:40, and 80:20, *v*/*v*). Fraction G3a (230 mg) was purified by semipreparative HPLC (3.0 mL/min, 0–25 min MeCN/H_2_O (60:40)) to yield **6** (9.0 mg, *t*_R_ 9.8 min), **8** (8.0 mg, *t*_R_ 13.4 min), **9** (5.0 mg, *t*_R_ 16.9 min), and **14** (7.0 mg, *t*_R_ 22.1 min). Fraction G3e (60 mg) was purified by semipreparative HPLC (3.0 mL/min, 0–25 min MeCN/H_2_O (60:40)) to yield **10** (8.0 mg, *t*_R_ 12.8 min), **13** (7.0 mg, *t*_R_ 15.4 min), **15** (9.0 mg, *t*_R_ 18.9 min), and **17** (5.0 mg, *t*_R_ 21.1 min). Fraction H (5.0 g) was subjected to a Sephadex LH-20 column eluted with MeOH–CH_2_Cl_2_ (1:1, *v*/*v*) and produced three subfractions (H1–H3). Fraction H2 (132 mg) was purified by semipreparative HPLC (3.0 mL/min, 0–25 min MeCN/H_2_O (60:40)) to yield **16** (7.0 mg, *t*_R_ 15.4 min) and **11** (9.0 mg, *t*_R_ 19.1 min). 

Chiral separations of **1**–**16** were performed on a semipreparative NP-HPLC using a Chiralpak AD-H column (4.6 mm × 250 mm, 5 mm, Daicel, Nanning, China), eluting with *n*-hexane-isopropanol in a ratio of 85:15 (*v*/*v*), 75:25 (*v*/*v*), 75:25 (*v*/*v*), 75:25 (*v*/*v*), 75:25 (*v*/*v*), 75:25 (*v*/*v*), 75:25 (*v*/*v*), 70:30 (*v*/*v*), 75:25 (*v*/*v*), 75:25 (*v*/*v*), 78:22 (*v*/*v*), 60:40 (*v*/*v*), 60:40 (*v*/*v*), 60:40 (*v*/*v*), 60:40 (*v*/*v*), and 60:40 (*v*/*v*), respectively. The detection wavelength was 238 nm and the flow rate was 1 mL/min. Finally, compounds **1a** (3.8 mg, *t*_R_ 6.0 min), **1b** (3.8 mg, *t*_R_ 8.0 min), **2a** (2.6 mg, *t*_R_ 6.2 min), **2b** (2.9 mg, *t*_R_ 8.0 min), **3a** (2.4 mg, *t*_R_ 4.4 min), **3b** (2.3mg, *t*_R_ 5.8 min), **4a** (1.8 mg, *t*_R_ 4.3 min), **4b** (1.9 mg, *t*_R_ 6.8 min), **5a** (4.7 mg, *t*_R_ 6.3 min), **5b** (5.0 mg, *t*_R_ 7.8 min), **6a** (3.9 mg, *t*_R_ 6.1 min), **6b** (4.2 mg, *t*_R_ 7.6 min), **7a** (2.3 mg, *t*_R_ 3.4 min), **7b** (2.5 mg, *t*_R_ 5.4 min), **8a** (3.6 mg, *t*_R_ 4.8 min), **8b** (3.8 mg, *t*_R_ 7.1 min), **9a** (2.4 mg, *t*_R_ 4.0 min), **9b** (2.4 mg, *t*_R_ 6.8 min), **10a** (3.8 mg, *t*_R_ 3.9 min), **10b** (3.8 mg, *t*_R_ 6.3 min), **11a** (4.3 mg, *t*_R_ 5.4 min), **11b** (4.5 mg, *t*_R_ 7.5 min), **12a** (1.9 mg, *t*_R_ 2.6 min), **12b** (2.0 mg, *t*_R_ 7.5 min), **13a** (3.3 mg, *t*_R_ 4.2 min), **13b** (3.5 mg, *t*_R_ 8.3 min), **14a** (3.4 mg, *t*_R_ 2.6 min), **14b** (3.4 mg, *t*_R_ 7.8 min), **15a** (4.3 mg, *t*_R_ 5.4 min), **15b** (4.5 mg, *t*_R_ 7.6 min), **16a** (3.3 mg, *t*_R_ 4.4 min), and **16b** (3.5 mg, *t*_R_ 7.9 min) were yielded, respectively.

#### 4.3.1. (±)-. Microphyltrine A (**1**)

White amorphous powder; UV (MeOH) *λ*_max_ (log *ε*) 227 (4.64), 252 (4.63), 293 (2.60), 332 (2.06) nm; IR (KBr) *ν*_max_ 3421, 1633, 1454, 1259, 1129, 1025, 670 cm^−1^; ^1^H and ^13^C NMR data, see Table 1; HRESIMS *m*/*z* 824.3330 [M − H]^−^ (calcd. for C_52_H_46_N_3_O_7_, 824.3336). 

*(−)-Microphyltrine A (**1a**)*: White amorphous powder, ${\left[\alpha \right]}_{D}^{25}$ −74 (*c* 0.1, MeOH); ECD (MeOH) *λ*_max_ (Δ*ε*) 218 (+13.79), 245 (−10.01) nm. 

*(+)-Microphyltrine A (**1b**)*: White amorphous powder, ${\left[\alpha \right]}_{D}^{25}$ +84 (*c* 0.1, MeOH); ECD (MeOH) *λ*_max_ (Δ*ε*) 218 (−13.68), 245 (+10.30) nm.

#### 4.3.2. (±)-. Microphyldine A (**2**)

Brown amorphous powder; UV (MeOH) *λ*_max_ (log *ε*) 224 (3.85), 247 (3.86), 305 (3.33), 340 (2.73) nm; IR (KBr) *ν*_max_ 3413, 2968, 1720, 1628, 1462, 1301, 1128, 1025, 890, 838, 728 cm^−1^; ^1^H and ^13^C NMR data, see Table 2 and Table 3; HRESIMS *m*/*z* 585.2387 [M − H]^−^ (calcd for C_37_H_33_N_2_O_5_, 585.2389).

*(−)-Microphyldine A (**2a**)*: Brown amorphous powder, ${\left[\alpha \right]}_{D}^{25}$ −20 (*c* 0.01, MeOH); ECD (MeOH) *λ*_max_ (Δ*ε*) 224 (+14.81), 252 (−7.66) nm. 

*(+)-Microphyldine A (**2b**)*: Brown amorphous powder, ${\left[\alpha \right]}_{D}^{25}$ +20 (*c* 0.01, MeOH); ECD (MeOH) *λ*_max_ (Δ*ε*) 224 (−14.73), 252 (+7.68) nm.

#### 4.3.3. (±)-. Microphyldine B (**3**)

Brown amorphous powder; UV (MeOH) *λ*_max_ (log *ε*) 229 (4.37), 301 (2.16) nm; IR (KBr) *ν*_max_ 3552, 2968, 2919, 1642, 1453, 1375, 1168, 1128, 1023, 891, 577, 451 cm^−1^; ^1^H and ^13^C NMR data, see Table 2 and Table 3; HRESIMS *m*/*z* 615.2486 [M − H]^−^ (calcd for C_38_H_35_N_2_O_6_, 615.2495).

*(−)-Microphyldine B (**3a**)*: Brown amorphous powder, ${\left[\alpha \right]}_{D}^{25}$ −100 (*c* 0.01, MeOH); ECD (MeOH) *λ*_max_ (Δ*ε*) 226 (+13.87), 250 (−7.95) nm.

*(+)-Microphyldine B (**3b**)*: Brown amorphous powder, ${\left[\alpha \right]}_{D}^{25}$ +100 (*c* 0.01, MeOH); ECD (MeOH) *λ*_max_ (Δ*ε*) 226 (−14.23), 250 (+8.39) nm.

#### 4.3.4. (±)-. Microphyldine C (**4**)

Brown amorphous powder; UV (MeOH) *λ*_max_ (log *ε*) 228 (4.86), 303 (3.26) nm; IR (KBr) *ν*_max_ 3418, 2969, 2922, 1715, 1640, 1444, 1423, 1377, 1186, 1128, 1024, 663 cm^−1^; ^1^H and ^13^C NMR data, see Table 2 and Table 3; HRESIMS *m*/*z* 585.2389 [M − H]^−^ (calcd for C_37_H_33_N_2_O_5_, 585.2389).

*(−)-Microphyldine C (**4a**)*: Brown amorphous powder, ${\left[\alpha \right]}_{D}^{25}$ −80 (*c* 0.01, MeOH); ECD (MeOH) *λ*_max_ (Δ*ε*) 226 (+81.61), 252 (−32.80) nm.

*(+)-Microphyldine C (**4b**)*: Brown amorphous powder, ${\left[\alpha \right]}_{D}^{25}$ +80 (*c* 0.01, MeOH); ECD (MeOH) *λ*_max_ (Δ*ε*) 226 (−79.45), 254 (+32.11) nm.

#### 4.3.5. (±)-. Microphyldine D (**5**)

Brown amorphous powder; UV (MeOH) *λ*_max_ (log *ε*) 227 (4.86), 248 (4.83), 307 (3.26) nm; IR (KBr) *ν*_max_ 3444, 2922, 1746, 1644, 1454, 1428, 1185, 1020, 578 cm^−1^; ^1^H and ^13^C NMR data, see Table 2 and Table 3; HRESIMS *m*/*z* 653.3023 [M − H]^−^ (calcd for C_42_H_41_N_2_O_5_, 653.3015).

*(−)-Microphyldine D (**5a**)*: Brown amorphous powder, ${\left[\alpha \right]}_{D}^{25}$ −80 (*c* 0.01, MeOH); ECD (MeOH) *λ*_max_ (Δ*ε*) 222 (+9.61), 252 (−6.80), 296 (−2.78) nm.

*(+)-Microphyldine D (**5b**)*: Brown amorphous powder, ${\left[\alpha \right]}_{D}^{25}$ +80 (*c* 0.01, MeOH); ECD (MeOH) *λ*_max_ (Δ*ε*) 222 (−9.45), 254 (+7.11), 298 (+2.63) nm.

#### 4.3.6. (±)-. Microphyldine E (**6**)

Brown amorphous powder; UV (MeOH) *λ*_max_ (log *ε*) 217 (4.86), 224 (4.43), 300 (3.26) nm; IR (KBr) *ν*_max_ 3445, 2979, 2901, 2123, 1646, 1454, 1383, 1160, 1044, 577 cm^−1^; ^1^H and ^13^C NMR data, see Table 2 and Table 3; HRESIMS *m*/*z* 653.3023 [M − H]^−^ (calcd for C_42_H_41_N_2_O_5_, 653.3015).

*(−)-Microphyldine E (**6a**)*: Brown amorphous powder, ${\left[\alpha \right]}_{D}^{25}$ −80 (*c* 0.01, MeOH); ECD (MeOH) *λ*_max_ (Δ*ε*) 218 (+3.31), 272 (−0.50) nm.

*(+)-Microphyldine E (**6b**)*: Brown amorphous powder, ${\left[\alpha \right]}_{D}^{25}$ +80 (*c* 0.01, MeOH); ECD (MeOH) *λ*_max_ (Δ*ε*) 218 (−3.15), 272 (+0.50) nm.

#### 4.3.7. (±)-. Microphyldine F (**7**)

Brown amorphous powder; UV (MeOH) *λ*_max_ (log *ε*) 227 (4.86), 248.8 (4.83), 296.6 (2.80) nm; IR (KBr) *ν*_max_ 3646, 3612, 3361, 2921, 2851, 1713, 1642, 1503, 1458, 1379, 1143, 1019, 576 cm^−1^; ^1^H and ^13^C NMR data, see Table 2 and Table 3; HRESIMS *m*/*z* 585.2390 [M − H]^−^ (calcd for C_37_H_33_N_2_O_5_ 585.2389).

*(−)-Microphyldine F (**7a**)*: Brown amorphous powder, ${\left[\alpha \right]}_{D}^{25}$ −80 (*c* 0.01, MeOH); ECD (MeOH) *λ*_max_ (Δ*ε*) 218 (+7.11), 248 (−3.10) nm.

*(+)-Microphyldine F (**7b**)*: Brown amorphous powder, ${\left[\alpha \right]}_{D}^{25}$ +80 (*c* 0.01, MeOH); ECD (MeOH) *λ*_max_ (Δ*ε*) 218 (−7.45), 248 (+2.91) nm.

#### 4.3.8. (±)-. Microphyldine G (**8**)

Brown amorphous powder; UV (MeOH) *λ*_max_ (log *ε*) 227 (4.86), 296.6 (4.80), 334 (4.26) nm; IR (KBr) *ν*_max_ 3647, 3420, 2966, 2920, 2851, 1728, 1608, 1448, 1428, 1185, 1018, 578 cm^−1^; ^1^H and ^13^C NMR data, see Table 2 and Table 3; HRESIMS *m*/*z* 653.3007 [M − H]^−^ (calcd for C_42_H_41_N_2_O_5_, 653.3015).

*(−)-Microphyldine G (**8a**)*: Brown amorphous powder, ${\left[\alpha \right]}_{D}^{25}$ −80 (*c* 0.01, MeOH); ECD (MeOH) *λ*_max_ (Δ*ε*) 200 (+8.61), 242 (−1.80) nm.

*(+)-Microphyldine G (**8b**)*: Brown amorphous powder, ${\left[\alpha \right]}_{D}^{25}$ +80 (*c* 0.01, MeOH); ECD (MeOH) *λ*_max_ (Δ*ε*) 200 (−8.45), 244 (+2.11) nm.

#### 4.3.9. (±)-. Microphyldine H (**9**)

Brown amorphous powder; UV (MeOH) *λ*_max_ (log *ε*) 238.8 (4.83), 309 (4.80), 334 (4.26) nm; IR (KBr) *ν*_max_ 3712, 3444, 2920, 1729, 1610, 1454, 1419, 1175, 1019, 575 cm^−1^; ^1^H and ^13^C NMR data, see Table 2 and Table 3; HRESIMS *m*/*z* 601.2692 [M − H]^−^ (calcd for C_38_H_37_N_2_O_5_, 601.2702).

*(−)-Microphyldine H (**9a**)*: Brown amorphous powder, ${\left[\alpha \right]}_{D}^{25}$ −80 (*c* 0.01, MeOH); ECD (MeOH) *λ*_max_ (Δ*ε*) 218 (+23.61), 254 (−13.80) nm.

*(+)-Microphyldine H (**9b**)*: Brown amorphous powder, ${\left[\alpha \right]}_{D}^{25}$ +80 (*c* 0.01, MeOH); ECD (MeOH) *λ*_max_ (Δ*ε*) 218 (−23.45), 254 (+13.11) nm.

#### 4.3.10. (±)-. Microphyldine I (**10**)

Brown amorphous powder; UV (MeOH) *λ*_max_ (log *ε*) 234 (4.86), 248 (4.83), 291 (4.80), 334 (4.26) nm; IR (KBr) *ν*_max_ 3419, 2969, 2920, 1714, 1613, 1453, 1387, 1174, 1019, 582 cm^−1^; ^1^H and ^13^C NMR data, see Table 2 and Table 3; HRESIMS *m*/*z* 391.1440 [M − H]^−^ (calcd for C_26_H_19_N_2_O_2_, 391.1447).

*(−)-Microphyldine I (**10a**)*: Brown amorphous powder, ${\left[\alpha \right]}_{D}^{25}$ −80 (*c* 0.01, MeOH); ECD (MeOH) *λ*_max_ (Δ*ε*) 203 (+11.61), 275 (−24.80) nm.

*(+)-Microphyldine I (**10b**)*: Brown amorphous powder, ${\left[\alpha \right]}_{D}^{25}$ +80 (*c* 0.01, MeOH); ECD (MeOH) *λ*_max_ (Δ*ε*) 204 (−19.45), 275 (+25.11) nm.

#### 4.3.11. (±)-. Microphyldine J (**11**)

Brown amorphous powder; UV (MeOH) *λ*_max_ (log *ε*) 226 (4.85), 243 (4.81), 294 (4.76), 334 (4.26) nm; IR (KBr) *ν*_max_ 3404, 1746, 1633, 1470, 1453, 1111, 1019, 579 cm^−1^; ^1^H and ^13^C NMR data, see Table 2 and Table 3; HRESIMS *m*/*z* 405.1232 [M − H]^−^ (calcd for C_26_H_17_N_2_O_3_, 405.1239).

*(−)-Microphyldine J (**11a**)*: Brown amorphous powder, ${\left[\alpha \right]}_{D}^{25}$ −80 (*c* 0.01, MeOH); ECD (MeOH) *λ*_max_ (Δ*ε*) 218 (−12.61), 272 (+25.20), 266 (−16.28) nm.

*(+)-Microphyldine J (**11b**)*: Brown amorphous powder, ${\left[\alpha \right]}_{D}^{25}$ +80 (*c* 0.01, MeOH); ECD (MeOH) *λ*_max_ (Δ*ε*) 218 (+19.45), 274 (−24.11), 268 (+16.63) nm.

#### 4.3.12. (±)-. Microphyldine K (**12**)

Brown amorphous powder; UV (MeOH) *λ*_max_ (log *ε*) 238 (4.83), 306 (4.80), 324 (4.26) nm; IR (KBr) *ν*_max_ 3601, 3425, 2969, 2917, 2855, 1720, 1631, 1464, 1135, 1025, 584 cm^−1^; ^1^H and ^13^C NMR data, see Table 4; HRESIMS *m*/*z* 505.2125 [M − H]^−^ (calcd for C_32_H_29_N_2_O_4_, 505.2127).

*(−)-Microphyldine K (**12a**)*: Brown amorphous powder, ${\left[\alpha \right]}_{D}^{25}$ −80 (*c* 0.01, MeOH); ECD (MeOH) *λ*_max_ (Δ*ε*) 214 (+19.61), 252 (−8.80), 296 (−5.78) nm.

*(+)-Microphyldine K (**12b**)*: Brown amorphous powder, ${\left[\alpha \right]}_{D}^{25}$ +80 (*c* 0.01, MeOH); ECD (MeOH) *λ*_max_ (Δ*ε*) 213 (−19.45), 254 (+9.11), 298 (+5.63) nm.

#### 4.3.13. (±)-. Microphyldine L (**13**)

Brown amorphous powder; UV (MeOH) *λ*_max_ (log *ε*) 243 (4.76), 300 (4.30), 334 (4.26) nm; IR (KBr) *ν*_max_ 3525, 3459, 3363, 2969, 2923, 2855, 1706, 1622, 1450, 1420, 1186, 1047, 580 cm^−1^; ^1^H and ^13^C NMR data, see Table 4; HRESIMS *m*/*z* 587.2543 [M − H]^−^ (calcd for C_37_H_35_N_2_O_5_, 587.2546).

*(−)-Microphyldine L (**13a**)*: Brown amorphous powder, ${\left[\alpha \right]}_{D}^{25}$ −80 (*c* 0.01, MeOH); ECD (MeOH) *λ*_max_ (Δ*ε*) 222 (−4.61), 296 (−3.78) nm.

*(+)-Microphyldine L (**13b**)*: Brown amorphous powder, ${\left[\alpha \right]}_{D}^{25}$ +80 (*c* 0.01, MeOH); ECD (MeOH) *λ*_max_ (Δ*ε*) 222 (+4.45), 298 (+3.63) nm.

#### 4.3.14. (±)-. Microphyldine M (**14**)

Brown amorphous powder; UV (MeOH) *λ*_max_ (log *ε*) 224 (4.86), 238.8 (4.83), 296.6 (4.80), 334 (4.26) nm; IR (KBr) *ν*_max_ 3525, 3459, 3363, 2969, 2923, 2855, 1706, 1622, 1450, 1420, 1186, 1047, 580 cm^−1^; ^1^H and ^13^C NMR data, see Table 4; HRESIMS *m*/*z* 601.2712 [M − H]^−^ (calcd for C_38_H_37_N_2_O_5_, 601.2702).

*(−)-Microphyldine M (**14a**)*: Brown amorphous powder, ${\left[\alpha \right]}_{D}^{25}$ −80 (*c* 0.01, MeOH); ECD (MeOH) *λ*_max_ (Δ*ε*) 208 (+6.81), 242 (−7.20), 296 (−2.78) nm.

*(+)-Microphyldine M (**14b**)*: Brown amorphous powder, ${\left[\alpha \right]}_{D}^{25}$ +80 (*c* 0.01, MeOH); ECD (MeOH) *λ*_max_ (Δ*ε*) 208 (−6.45), 244 (+7.11), 296 (+2.63) nm.

#### 4.3.15. (±)-. Microphyldine N (**15**)

Brown amorphous powder; UV (MeOH) *λ*_max_ (log *ε*) 224 (4.86), 245 (4.83), 301 (4.80), 334 (4.26) nm; IR (KBr) *ν*_max_ 3725, 3600, 3383, 2964, 2917, 2850, 1721, 1617, 1494, 1475, 1259, 1025, 580 cm^−1^; ^1^H and ^13^C NMR data, see Table 4; HRESIMS *m*/*z* 655.3156 [M − H]^−^ (calcd for C_42_H_43_N_2_O_5_, 655.3172).

*(−)-Microphyldine N (**15a**)*: Brown amorphous powder, ${\left[\alpha \right]}_{D}^{25}$ −80 (*c* 0.01, MeOH); ECD (MeOH) *λ*_max_ (Δ*ε*) 214 (+1.21), 248 (−1.10), 326 (−0.78) nm.

*(+)-Microphyldine N (**15b**)*: Brown amorphous powder, ${\left[\alpha \right]}_{D}^{25}$ +80 (*c* 0.01, MeOH); ECD (MeOH) *λ*_max_ (Δ*ε*) 213 (−1.45), 247 (+1.11), 328 (+0.63) nm.

#### 4.3.16. (±)-. Microphyldine O (**16**)

Brown amorphous powder; UV (MeOH) *λ*_max_ (log *ε*) 226 (4.86), 238.8 (4.83), 302 (4.80), 334 (4.26) nm; IR (KBr) *ν*_max_ 3725, 3624, 3383, 2920, 2849, 1719, 1617, 1476, 1438, 1162, 1028, 580 cm^−1^; ^1^H and ^13^C NMR data, see Table 4; HRESIMS *m*/*z* 669.3328 [M − H]^−^ (calcd for C_43_H_45_N_2_O_5_, 669.3328).

*(−)-Microphyldine O (**16a**)*: Brown amorphous powder, ${\left[\alpha \right]}_{D}^{25}$ −80 (*c* 0.01, MeOH); ECD (MeOH) *λ*_max_ (Δ*ε*) 218 (+5.91), 252 (−5.80), 296 (−0.78) nm.

*(+)-Microphyldine O (**16b**)*: Brown amorphous powder, ${\left[\alpha \right]}_{D}^{25}$ +80 (*c* 0.01, MeOH); ECD (MeOH) *λ*_max_ (Δ*ε*) 218 (−5.85), 254 (+5.71), 298 (+0.93) nm.

#### 4.3.17. Microphyldine P (**17**)

Brown amorphous powder; UV (MeOH) *λ*_max_ (log *ε*) 245 (4.83), 294 (4.80), 334 (4.26) nm; IR (KBr) *ν*_max_ 3442, 2954, 2920, 2850, 1734, 1656, 1494, 1458, 1214, 1024, 578 cm^−1^; ^1^H and ^13^C NMR data see, Table 2 and Table 3; HRESIMS *m*/*z* 487.2016 [M − H]^−^ (calcd for C_32_H_27_N_2_O_3_, 487.2022).

### 4.4. Computational Methods

The *R*_a_/*S*_a_ configurations of compounds **1**, **2**, **6**, **8**, **10**, **11** and the (1′*S*)/(1′*R*) configurations of compounds **12**–**16** were submitted to random conformational analysis, respectively, with the MMFF94s force field using the Sybyl-X 2.0 software package. The conformers were further optimized by using the TDDFT method at the B3LYP/6-31G(d) level, and the frequency was calculated at the same level of theory. The stable conformers without imaginary frequencies were subjected to ECD calculation by the TDDFT method at the B3LYP/6-31+G(d) level with the CPCM model in MeOH. ECD spectra of different conformers were simulated using SpecDis v1.51 with a half-band width of 0.3 eV, and the final ECD spectra were computed according to the Boltzmann-calculated contribution of each conformer. The calculated ECD spectra were compared with the experimental data. All calculations were performed with the Gaussian 09 program package [26,27].

### 4.5. Anti-Inflammatory Activity Assay

The murine BV-2 microglial cells or the monocytic RAW 264.7 macrophages were purchased from Peking Union Medical College (PUMC) Cell Bank (Beijing, China). Cell maintenance, experimental procedures, and data determination for the inhibition of NO production are the same as previously described [15,28]. Cell viability was evaluated by MTT assay. Dexamethasone was used as a positive control. 

### 4.6. Cytotoxicity Assay

HepG2, Du145, HCT116, and HeLa cells (PUMC Cell Bank, Beijing, China) were used for the cytotoxicity assays. Cytotoxic activities were determined using the MTT method. Cell culture, experimental procedures, and data processing were performed according to the literature report [29], with taxol serving as a positive control.

## Figures and Tables

**Table 1:** ^1^H NMR and ^13^C NMR data of compound **1** in acetone-*d*_6_ (*δ* in ppm, *J* in Hz, ^1^H NMR: 400 MHz; ^13^C NMR: 100 MHz) ***^a^***.

Position	*δ*_H_	*δ*_C_	Position	*δ*_H_	*δ*_C_
1		105.1, C	1‴	6.66, d (10.0)	119.9, CH
2		149.5, C	2‴	5.48, d (10.0)	129.6, CH
3		117.9, C	3‴		76.5, C
4	7.62, s	120.9, CH	4‴	1.38, s	28.1, CH_3_
4a		119.3, C	5‴	1.37, s	28.4, CH_3_
4b		115.9, C	1⁗		147.6, C
5	7.60, s	105.9, CH	2⁗	7.15, d (2.0)	108.4, CH
6		144.1, C	3⁗		132.9, C
7		144.5, C	4⁗	7.56, d (2.0)	112.9, CH
8		105.1, C	4a⁗		125.7, C
8a		136.6, C	4b⁗		124.6, C
9a		136.5, C	5⁗	7.91, d (8.0)	121.3, CH
1′	6.81, d (10.0)	119.8, CH	6⁗	7.10, t (8.0)	120, CH
2′	5.59, d (10.0)	129.3, CH	7⁗	7.34, t (8.0)	126.6, CH
3′		76.7, C	8⁗	7.55, d (8.0)	112.6, CH
4′	1.49, s	27.9, CH_3_	8a⁗		141.6, C
5′	1.45, s	28.5, CH_3_	9a⁗		130.1, C
1″		106.2, C	3-CH_3_	2.28, s	16.8, CH_3_
2″		149.5, C	6-OCH_3_	4.01, s	57.7, CH_3_
3″		117.7, C	7-OH	7.16, br s	
4″		134, C	9-NH	9.58, br s	
4a″		118.6, C	3″-CH_3_	2.42, s	12, CH_3_
4b″		115.9, C	6″-OCH_3_	3.82, s	57.4, CH_3_
5″	7.59, s	103, CH	7″-OH	7.05, br s	
6″		143.5, C	9″-NH	9.55, br s	
7″		145, C	1⁗-OCH_3_	3.98, s	56.4, CH_3_
8″		105.4, C	3⁗-CH_2_	4.84, s	37.3, CH_2_
8a″		136.6, C	9⁗-NH	10.25, br s	
9a″		136.6, C			

***^a^*** Assignments were based on HSQC and HMBC experiments.

**Table 2:** ^1^H NMR data of compounds **2**–**11** and **17** in acetone-*d*_6_ (*δ* in ppm, *J* in Hz) ***^a^***.

Position	2	3	4	5 *^b^*	6 *^b^*	7	8	9 *^c^*	10 *^b^*	11 *^b^*	17
**2**									7.03, s		
**4**	7.65, s	7.66, s	7.66, s	7.67, s	7.62, s	7.66, s	7.65, s	7.64, s			7.49, s
**5**	7.61, s	7.71, s	7.72, s	7.73, s	7.60, s	7.63, s	7.63, s	7.58, s	7.44, d (8.0)	8.31, d (8.0)	7.68, s
**6**									7.12, t (8.0)	7.43, t (8.0)	
**7**									6.59, t (8.0)	7.49, t (8.0)	
**8**									6.52, d (8.0)	7.71, d (8.0)	6.69, s
**1′**	6.87, d (10.0)	6.66, d (10.0)	6.64, d (10.0)	6.64, d (10.0)	6.86, d (10.0)	6.72, d (10.0)	6.74, d (10.0)	6.37, d (10.0)			6.87, d (10.0)
**2′**	5.58, d (10.0)	5.51, d (10.0)	5.49, d (10.0)	5.50, d (10.0)	5.58, d (10.0)	5.55, d (10.0)	5.53, d (10.0)	5.57, d (10.0)	7.03, s	6.92, s	5.74, d (10.0)
**4′**	1.43, s	1.43, s	1.38, s	1.39, s	1.43, s	1.41, s	1.41, s	1.47, s			1.45, s
**5′**	1.42, s	1.42, s	1.35, s	1.36, s	1.43, s	1.41, s	1.41, s	1.47, s	7.44, d (8.0)	7.78, d (8.0)	1.45, s
**6′**									7.12, t (8.0)	6.94, t (8.0)	
**7′**									6.59, t (8.0)	7.29, t (8.0)	
**8′**									6.52, d (8.0)	7.56, d (8.0)	
**9′**											
**10′**											
**2″**											7.03, s
**4″**	7.63, s	6.44, s	6.42, s	6.43, s	7.65, s	7.67, s	7.67, s	7.86, s			7.65, s
**5″**	7.87, s				7.86, s	7.82, d (8.0)	7.81, d (8.0)	7.81, d (8.0)			8.01, d (8.0)
**6″**						6.87, d (8.0)	6.86, d (8.0)	6.87, d (8.0)			7.13, t (8.0)
**7″**			7.00, d (8.0)	7.00, d (8.0)							7.35, t (8.0)
**8″**	7.04, s	7.09, s	7.31, d (8.0)	7.32, d (8.0)	7.03, s						7.55, d (8.0)
**1‴**	6.94, d (10.0)	6.87, d (10.0)	6.87, d (10.0)	6.92, d (10.0)	6.98, d (10.0)	6.75, d (10.0)	6.75, d (10.0)	3.44, d (7.2)			
**2‴**	5.80, d (10.0)	5.58, d (10.0)	5.69, d (10.0)	5.69, d (10.0)	5.78, d (10.0)	5.56, d (10.0)	5.57, d (10.0)	5.12, t (7.2)			
**4‴**	1.50, s	1.39, s	1.38, s	1.50, s	1.47, s	1.41, s	1.39, s	1.26, s			
**5‴**	1.49, s	1.36, s	1.38, s	1.70, m	1.80, m	1.41, s	1.71, m	1.26, s			
**6‴**				2.11, m	2.23, m		2.15, m				
**7‴**				5.08, t (6.0)	5.16, t (6.0)		5.11, t (6.0)				
**9‴**				1.61, s	1.66, s		1.63, s				
**10‴**				1.52, s	1.59, s		1.55, s				
**1-OH**									8.65, br s		
**3-CH_3_**	2.31, s	2.29, s	2.28, s	2.28, s	2.31, s	2.31, s	1.81, s	2.37, s	2.06, s	1.89, s	2.23, s
**6-OCH_3_**	4.02, s	4.05, s	4.04, s	4.04, s	4.08, s	4.04, s	4.03, s	4.13, s			
**7-OH**	7.43, br s	7.08, br s	7.23, br s	7.23, br s	7.62, br s	7.66, br s	7.00, br s	6.21, br s			8.20, br s
**9-NH**	9.58, br s	9.40, br s	9.40, br s	9.40, br s	9.57, br s	9.58, br s	10.02, br s	7.47, br s	10.11, br s	11.70, br s	9.92, br s
**1′-OH**									8.65, br s	8.80, br s	
**3′-CH_3_**									2.06, s	2.25, s	
**9′-NH**									10.11, br s	10.26, br s	
**1″-OCH_3_**											3.96, s
**2″-OH**								5.56, br s			
**3″-CH2**											4.28, s
**3″-CH_3_**	2.29, s	1.85, s	1.81, s	1.81, s	2.30, s	2.32, s	2.33, s	2.54, s			
**6″-OH**			7.02, br s								
**6″-OCH_3_**		3.99, s									
**7″-OH**	7.43, br s	6.71, br s		7.00, br s	7.42, br s	7.67, br s	7.67, br s				
**7″-OCH_3_**								3.92, s			
**9″-NH**	10.14, br s	9.95, br s	10.02, br s	10.02, br s	10.15, br s	9.66, br s	9.67, br s	7.66, br s			10.20, br s

*^a^* Assignments were based on HSQC and HMBC experiments.^*b*^ Measured in 500 MHz, and others in 400 MHz. *^c^* **9** was measured in CDCl_3_ and others were measured in acetone-*d*_6_.

**Table 3:** ^13^C NMR data of compounds **2**–**11** and **17** (Measured in acetone-*d*_6_, *δ* in ppm) ***^a^***.

Position	2	3	4	5 *^b^*	6 *^b^*	7	8	9 *^c^*	10 *^b^*	11 *^b^*	17
**1**	104.9, C	104.7, C	104.9, C	104.3, C	104.9, C	104.9, C	104.7, C	104.9, C	141.8, C	179.1, C	104.5, C
**2**	148.3, C	148.1, C	149.1, C	147.7, C	148.3, C	148.2, C	148.2, C	149.1, C	112.6, CH	142.2, C	148.2, C
**3**	116.8, C	116.5, C	118.1, C	116.2, C	116.9, C	116.8, C	118.0, C	118.7, C	126.8, C	145.6, C	116.8, C
**4**	119.7, CH	119.8, CH	120.7, CH	119.3, CH	120.4, CH	119.6, CH	119.7, CH	120.0, CH	125.0, C	183.1, C	120.0, CH
**4a**	117.9, C	118.0, C	118.8, C	117.4, C	117.9, C	114.8, C	116.7, C	116.2, C	123.9, C	116.5, C	117.3, C
**4b**	115.0, C	114.7, C	115.8, C	114.4, C	115.0, C	118.0, C	114.8, C	117.3, C	123.4, C	124.3, C	116.8, C
**5**	101.0, CH	101.7, CH	102.9, CH	101.5, CH	101.0, CH	101.8, CH	101.7, CH	101.8, CH	110.6, CH	122.4, CH	120.5, CH
**6**	142.3, C	142.8, C	143.8, C	142.4, C	142.7, C	142.8, C	142.8, C	142.1, C	124.6, CH	123.8, CH	121.4, C
**7**	142.7, C	143.4, C	144.5, C	143.1, C	142.8, C	143.8, C	143.8, C	141.6, C	118.0, CH	126.5, CH	153.2, C
**8**	109.1, C	106.6, C	106.7, C	109.5, C	109.0, C	103.5, C	103.5, C	101.7, C	121.2, CH	113.6, CH	96.5, CH
**8a**	135.1, C	134.9, C	135.9, C	134.5, C	135.1, C	135.3, C	135.5, C	132.7, C	140.4, C	138.0, C	140.1, C
**9a**	135.3, C	135.3, C	136.2, C	134.8, C	135.3, C	135.7, C	135.1, C	134.7, C	128.5, C	136.2, C	135.2, C
**1′**	118.4, CH	118.3, CH	119.2, CH	117.8, CH	118.4, CH	118.3, CH	118.3, CH	117.3, CH	141.8, C	142.6, C	117.9, CH
**2′**	128.3, CH	128.2, CH	129.2, CH	127.7, CH	128.3, CH	128.4, CH	128.4, CH	129.4, CH	112.6, CH	112.3, CH	128.9, CH
**3′**	75.2, C	75.1, C	76, C	74.6, C	75.2, C	75.2, C	75.1, C	75.7, C	126.8, C	118.6, C	75.3, C
**4′**	27, CH_3_	27, CH_3_	27.9, CH_3_	26.4, CH_3_	27, CH_3_	26.8, CH_3_	27, CH_3_	27.4, CH_3_	125, C	126.8, C	26.9, CH_3_
**4a′**									123.9, C	122.4, C	
**4b′**									123.4, C	123.0, C	
**5′**	26.9, CH_3_	26.8, CH_3_	27.8, CH_3_	26.4, CH_3_	26.9, CH_3_	26.8, CH_3_	27, CH_3_	27.5, CH_3_	110.6, CH	120.7, CH	26.9, CH_3_
**6′**									124.6, CH	118.8, CH	
**7′**									118.0, CH	125.1, CH	
**8′**									121.2, CH	111.3, CH	
**8a′**									140.4, C	140.4, C	
**9′**											
**9a′**									128.5, C	128.2, C	
**10′**											
**1″**	104.6, C	104, C	105.7, C	103.2, C	104.5, C	104.9, C	104.9, C	102.1, C			145.7, C
**2″**	148.6, C	148.1, C	150, C	148.7, C	148.7, C	148.3, C	148.4, C	150.0, C			107.8, CH
**3″**	117.1, C	116, C	117.1, C	115.4, C	116.8, C	117.0, C	117.7, C	118.1, C			133.7, C
**4″**	120.3, CH	121.8, CH	123.6, CH	122.3, CH	119.7, CH	119.7, CH	119.8, CH	121.6, CH			112.4, CH
**4a″**	117.3, C	117.7, C	118.7, C	116.7, C	117.2, C	117.7, C	116.7, C	118.5, C			124, C
**4b″**	117.5, C	116, C	124.3, C	122.8, C	117.5, C	116.8, C	116.8, C	118.7, C			123.4, C
**5″**	122.1, CH	112.8, C	113.6, C	112.2, C	122.0, CH	119.2, CH	119.6, CH	117.3, CH			119.9, CH
**6″**	114.2, C	139.2, C	149.6, C	148.2, C	114.2, CH	108.9, CH	108.8, C	104.7, CH			118.5, CH
**7″**	153.4, C	146.5, C	114.5, CH	113.1, CH	153.4, C	153.2, C	153.2, C	154.7, C			125.1, CH
**8″**	97.9, CH	93.9, CH	111.5, CH	110.1, CH	97.8, CH	103.7, C	103.7, C	111.0, C			111.2, CH
**8a″**	141.5, C	133.9, C	135.6, C	134.3, C	141.5, C	141.0, C	141.0, C	140.4, C			140.2, C
**9a″**	135.5, C	135.4, C	137.3, C	136.0, C	135.5, C	135.4, C	135.3, C	137.7, C			128.6, C
**1‴**	117.9, CH	117.9, CH	118.7, CH	117.7, CH	118.2, CH	118.3, CH	118.7, CH	24, CH_2_			
**2‴**	129.1, CH	128.5, CH	129.8, CH	127, CH	128.2, CH	128.5, CH	127.6, CH	122.1, CH			
**3‴**	75.4, C	75.2, C	76.3, C	77.3, C	77.7, C	75.2, C	77.5, C	132.7, C			
**4‴**	26.9, CH_3_	26.9, CH_3_	27.9, CH_3_	24.7, CH_3_	25.3, CH_3_	27, CH_3_	26.8, CH_3_	25, CH_3_			
**5‴**	26.9, CH_3_	26.8, CH_3_	27.8, CH_3_	40.2, CH_2_	40.7, CH_2_	26.8, CH_3_	40.6, CH_2_	16.9, CH_3_			
**6‴**				22.1, CH_2_	22.6, CH_2_		22.6, CH_2_				
**7‴**				123.8, CH	124.3, CH		124.3, CH				
**8‴**				130.4, C	131.0, C		130.9, C				
**9‴**				24.7, CH_3_	24.9, CH_3_		24.9, CH_3_				
**10‴**				16.2, CH_3_	16.7, CH_3_		16.7, CH_3_				
**3-CH_3_**	15.4, CH_3_	15.4, CH_3_	16.4, CH_3_	14.9, CH_3_	15.4, CH_3_	15.4, CH_3_	16.3, CH_3_	16.1, CH_3_	18.2, CH_3_	12.7, CH_3_	15.2, CH_3_
**6-OCH_3_**	56.3, CH_3_	56.6, CH_3_	57.5, CH_3_	56.1, CH_3_	56.3, CH_3_	56.3, CH_3_	56.3, CH_3_	56.9, CH_3_			
**3″-CH_3_**	15.3, CH_3_	15.5, CH_3_	16.3, CH_3_	15, CH_3_	15.3, CH_3_	15.5, CH_3_	15.4, CH_3_	16.9, CH_3_	18.2, CH_3_	18.4, CH_3_	
**3″-CH_2_**											36.4, CH_3_
**1″-OCH_3_**											54.9, CH_3_
**6″-OCH_3_**											
**7″-OCH_3_**		55.7, CH_3_						56.7, CH_3_			

*^a^* Assignments were based on HSQC and HMBC experiments.^*b*^ Measured in 500 MHz, and others in 400 MHz. *^c^* **9** was measured in CDCl_3_ and others were measured in acetone-*d*_6_.

**Table 4:** ^1^H NMR and ^13^C NMR data of compounds **12**–**16** in acetone-*d*_6_ (*δ* in ppm, *J* in Hz) ***^a^***.

Position	12		13		14 *^b^*		15 *^b^*		16 *^b^*	
	*δ*_H_	*δ*_C_	*δ*_H_	*δ*_C_	*δ*_H_	*δ*_C_	*δ*_H_	*δ*_C_	*δ*_H_	*δ*_C_
**1**		106.6, C		106.8, C		108.0, C		108.4, C		106.3, C
**2**		151.9, C		150.7, C		152.0, C		152.0, C		150.3, C
**3**		118.1, C		117.9, C		117.6, C		120.1, C		117.7, C
**4**	7.65, s	119.4, CH	7.62, s	118.5, CH	7.65, s	120.0, CH	7.63, s	119.5, CH	7.66, s	118.2, CH
**4a**		115.8, C		115.4, C		117.5, C		117.9, C		115.9, C
**4b**		124.4, C		116.5, C		117.0, C		116.8, C		115.3, C
**5**	7.32, d (2.0)	103.9, CH	7.47, s	101.8, CH	7.47, s	104.3, CH	7.48, s	103.3, CH	7.48, s	102.6, CH
**6**		150.6, C		145.0, C		145.7, C		143.8, C		143.9, C
**7**	6.61, dd (2.0, 8.0)	112.4, CH		142.4, C		149.6, C		146.4, C		147.9, C
**8**	6.87, d (8.0)	110.8, CH	6.55, d (8.0)	97.0, CH	6.69, s	96.7, CH	6.56, s	98.4, CH	6.70, s	94.9, CH
**8a**		134.0, C		134.8, C		135.7, C		136.2, C		133.9, C
**9a**		138.7, C		137.6, C		138.9, C		139.0, C		137.1, C
**1′**	4.94, dd (7.2, 10.0)	30.1, CH	4.93, dd (7.2, 10.0)	30.5, CH	4.93, dd (7.2, 10.0)	31.9, CH	4.94, dd (7.2, 10.0)	30.2, CH	4.95, dd (7.2, 10.0)	30.1, CH
**2′a**	2.07, dd (10.0, 14.0)	42.8, CH_2_	2.06, dd (10.0, 14.0)	43, CH_2_	2.08, dd (10.0, 14.0)	44.2, CH_2_	2.13, dd (10.0, 14.0)	44.4, CH_2_	2.05, dd (10.0, 14.0)	42.4, CH_2_
**2′b**	2.42, dd (7.2, 14.0)		2.39, dd (7.2, 14.0)		2.40, dd (7.2, 14.0)		2.41, dd (7.2, 14.0)		2.37, dd (7.2, 14.0)	
**3′**		74.4, C		74.2, C		75.4, C		75.6, C		73.8, C
**4′**	1.37, s	23.9, CH_3_	1.35, s	23.8, CH_3_	1.36, s	25.1, CH_3_	1.37, s	25.2, CH_3_	1.38, s	23.3, CH_3_
**5′**	1.45, s	29.1, CH_3_	1.44, s	29.3, CH_3_	1.45, s	30.4, CH_3_	1.46, s	30.4, CH_3_	1.47, s	28, CH_3_
**1″**		145.3, C		104.5, C		105.7, C		105.7, C		103.8, C
**2″**	6.63, d (2.0)	106.4, CH		148.4, C		149.6, C		149.9, C		148.0, C
**3″**		128.5, C		117.0, C		118.3, C		118.1, C		116.3, C
**4″**	7.01, d (2.0)	111.6, CH	7.25, s	120, CH	7.27, s	121.3, CH	7.27, s	121.5, CH	7.28, s	119.6, CH
**4a″**		124.5, C		117.0, C		119.4, C		118.3, C		117.6, C
**4b″**		117.1, C		117.4, C		118.2, C		118.8, C		116.9, C
**5″**	7.46, s	119.6, CH	7.39, s	118.7, CH	7.42, s	119.9, CH	7.41, s	119.9, CH	7.42, s	118.2, CH
**6″**		122.2, C		122.2, C		123.4, C		123.6, C		121.7, C
**7″**		153.9, C		153.0, C		154.2, C		154.3, C		152.5, C
**8″**	7.13, s	97.1, CH	7.02, s	97.0, CH	7.04, s	98.3, CH	7.04, s	98.4, CH	7.05, s	96.6, CH
**8a″**		140.2, C		140.2, C		141.4, C		141.5, C		139.7, C
**9a″**		127.9, C		135.2, C		136.5, C		136.2, C		134.8, C
**1‴**			6.83, d (10.0)	118, CH	6.84, d (10.0)	119, CH	6.88, d (10.0)	119.3, CH	6.89, d (10.0)	117.6, CH
**2‴**			5.72, d (10.0)	128.9, CH	5.72, d (10.0)	130.2, CH	5.71, d (10.0)	129.4, CH	5.72, d (10.0)	127.6, CH
**3‴**				75.3, C		76.6, C		79.1, C		77.2, C
**4‴**			1.41, s	27, CH_3_	1.41, s	28.1, CH_3_	1.40, s	26.2, CH_3_	1.40, s	24.7, CH_3_
**5‴**			1.41, s	27, CH_3_	1.40, s	28.1, CH_3_	1.73, m	42.0, CH_2_	1.73, m	40.1, CH_2_
**6‴**							2.15, m	23.9, CH_2_	2.15, m	22.1, CH_2_
**7‴**							5.11, t (6.0)	125.7, CH	5.11, t (6.0)	123.8, CH
**8‴**								132.3, C		130.4, C
**9‴**							1.62, s	26.2, CH_3_	1.61, s	24.7, CH_3_
**10‴**							1.54, s	18.1, CH_3_	1.54, s	16.2, CH_3_
**3-CH_3_**	2.36, s	16.3, CH_3_	2.36, s	16.3, CH_3_	2.37, s	17.6, CH_3_	2.37, s	17.7, CH_3_	2.38, s	15.8, CH_3_
**6-OH**	7.66, br s									
**6-OCH_3_**			3.88, s	56.2, CH_3_	3.82, s	57.4, CH_3_	3.89, s	57.6, CH_3_	3.83, s	55.7, CH_3_
**7-OH**			7.14, br s				7.12, br s			
**7-OCH_3_**					3.64, s	56.5, CH_3_			3.66, s	54.8, CH_3_
**9-NH**	8.55, br s		8.43, br s		8.40, br s		8.43, br s		8.42, br s	
**1″-OCH_3_**	3.91, s	54.8, CH_3_								
**3″-CH_3_**	2.30, s	20.8, CH_3_	2.12, s	15.0, CH_3_	2.13, s	16.3, CH_3_	2.16, s	16.4, CH_3_	2.15, s	14.6, CH_3_
**7″-OH**	8.23, br s		8.18, br s		8.14, br s		8.16, br s		8.15, br s	
**9″-NH**	9.88, br s		9.97, br s		9.97, br s		9.96, br s		9.98, br s	

***^a^*** Assignments were based on HSQC and HMBC experiments. ^b1^H NMR: 500 MHz; ^13^C NMR: 125 MHz. Others: ^1^H NMR: 400 MHz; ^13^C NMR: 100 MHz.

## Data Availability

Not applicable.

## Supplementary Materials

The following are available online, Figures S1, the HRESIMS, ^1^H NMR, ^13^C NMR, HSQC, and HMBC spectra of compounds **1**–**17**, along with the chiral HPLC separation and ECD data of compounds **3**–**11** and **13**–**16**.

## Sample Availability

Samples of the compounds are not available from the authors.
