# Supplementary material for: Trimeric and Dimeric Carbazole Alkaloids from Murraya microphylla
Source: Molecules. 2021 Sep 20;26(18):5689. doi: 10.3390/molecules26185689 (PMC8469674; doi:10.3390/molecules26185689)
Supplement: Supplementary file 1 [file molecules-26-05689-s001.zip › molecules-1365605-supplementary.pdf]

**SUPPLEMENTARY MATERIAL**

# Nitrogen Oxide Inhibitory Trimeric and Dimeric Carbazole Alkaloids from *Murraya microphylla*

Xiaoli Ma, Hongwei Chen, Sisi Zhu, Pengfei Tu and Yong Jiang*

*State Key Laboratory of Natural and Biomimetic Drugs, School of Pharmaceutical Sciences,*

*Peking University, Beijing 100191, P.R. China*

*Corresponding author. E-mail: yongjiang@bjmu.edu.cn.

# CONTENTS

**For compound 1**
**Figure S1** HRESIMS for compound **1**.
**Figure S2** UV spectrum for compound **1**.
**Figure S3** $^{1}$H NMR spectrum (400 MHz, acetone-$d_6$) of compound **1**.
**Figure S4** $^{13}$C NMR spectrum (100 MHz, acetone-$d_6$) of compound **1**.
**Figure S5** HSQC spectrum of compound **1**.
**Figure S6** HMBC spectrum of compound **1**.

**For compound 2**
**Figure S7** HRESIMS for compound **2**
**Figure S8** $^{1}$H NMR spectrum (400 MHz, acetone-$d_6$) of compound **2**.
**Figure S9** $^{13}$C NMR spectrum (100 MHz, acetone-$d_6$) of compound **2**.
**Figure S10** HSQC spectrum of compound **2**.
**Figure S11** HMBC spectrum of compound **2**.
**Figure S12** Key HMBC correlations of compounds **2.**

**For compound 3**
**Figure S13** HRESIMS for compound **3**
**Figure S14** $^{1}$H NMR spectrum (400 MHz, acetone-$d_6$) of compound **3**.
**Figure S15** $^{13}$C NMR spectrum (100 MHz, acetone-$d_6$) of compound **3**.
**Figure S16** HSQC spectrum of compound **3**.
**Figure S17** HMBC spectrum of compound **3**.
**Figure S18** The chiral HPLC separation and experimental ECD data of compounds **3a** and **3b**.
**Figure S19** Key HMBC correlations of compounds **3.**

**For compound 4**
**Figure S20** HRESIMS for compound **4**
**Figure S21** $^{1}$H NMR spectrum (400 MHz, acetone-$d_6$) of compound **4**.
**Figure S22** $^{13}$C NMR spectrum (100 MHz, acetone-$d_6$) of compound **4**.
**Figure S23** HSQC spectrum of compound **4**.
**Figure S24** HMBC spectrum of compound **4**.
**Figure S25** The chiral HPLC separation and experimental ECD data of compounds **4a** and **4b**.
**Figure S26** Key HMBC correlations of compounds **4.**

**For compound 5**
**Figure S27** HRESIMS for compound **5**.
**Figure S28** $^{1}$H NMR spectrum (500 MHz, acetone-$d_6$) of compound **5**.
**Figure S29** $^{13}$C NMR spectrum (125 MHz, acetone-$d_6$) of compound **5**.
**Figure S30** HSQC spectrum of compound **5**.
**Figure S31** HMBC spectrum of compound **5**.

**Figure S32** The chiral HPLC separation and experimental ECD data of compounds **5a** and **5b**.

**For compound 6**

**Figure S33** HRESIMS for compound **6**.
**Figure S34** $^{1}$H NMR spectrum (500 MHz, acetone-$d_6$) of compound **6**.
**Figure S35** $^{13}$C NMR spectrum (125 MHz, acetone-$d_6$) of compound **6**.
**Figure S36** HSQC spectrum of compound **6**.
**Figure S37** HMBC spectrum of compound **6**.
**Figure S38** The chiral HPLC separation and experimental and calculated ECD data of compounds **6a** and **6b**.
**Figure S39** Key HMBC correlations of compounds **6.**

**For compound 7**

**Figure S40** HRESIMS for compound **7**.
**Figure S41** $^{1}$H NMR spectrum (400 MHz, acetone-$d_6$) of compound **7**.
**Figure S42** $^{13}$C NMR spectrum (100 MHz, acetone-$d_6$) of compound **7**.
**Figure S43** HSQC spectrum of compound **7**.
**Figure S44** HMBC spectrum of compound **7**.
**Figure S45** The chiral HPLC separation and experimental ECD data of compounds **7a** and **7b**.
**Figure S46** Key HMBC correlations of compounds **7.**

**For compound 8**

**Figure S47** HRESIMS for compound **8**.
**Figure S48** $^{1}$H NMR spectrum (400 MHz, acetone-$d_6$) of compound **8**.
**Figure S49** $^{13}$C NMR spectrum (100 MHz, acetone-$d_6$) of compound **8**.
**Figure S50** HSQC spectrum of compound **8**.
**Figure S51** HMBC spectrum of compound **8**.
**Figure S52** The chiral HPLC separation and experimental and calculated ECD data of compounds **8a** and **8b**.
**Figure S53** Key HMBC correlations of compounds **8.**

**For compound 9**

**Figure S54** HRESIMS for compound **9**.
**Figure S55** $^{1}$H NMR spectrum (400 MHz, $CDCl_3$) of compound **9**.
**Figure S56** $^{13}$C NMR spectrum (100 MHz, $CDCl_3$) of compound **9**.
**Figure S57** HSQC spectrum of compound **9**.
**Figure S58** HMBC spectrum of compound **9**.
**Figure S59** The chiral HPLC separation and experimental ECD data of compounds **9a** and **9b**.
**Figure S60** Key HMBC correlations of compounds **9.**

**For compound 10**

**Figure S61** HRESIMS for compound **10**.

**Figure S62** $^{1}$H NMR spectrum (500 MHz, acetone-$d_6$) of compound **10**.

**Figure S63**$^{13}$C NMR spectrum (125 MHz, acetone-$d_6$) of compound **10**.

**Figure S64** HSQC spectrum of compound **10**.

**Figure S65** HMBC spectrum of compound **10**.

**Figure S66** The chiral HPLC separation and experimental and calculated ECD data of compounds **10a** and **10b**.

**Figure S67** Key HMBC correlations of compounds **10.**

**For compound 11**

**Figure S68** HRESIMS for compound **11**.

**Figure S69** $^{1}$H NMR spectrum (500 MHz, acetone-$d_6$) of compound **11**.

**Figure S70** $^{13}$C NMR spectrum (125 MHz, acetone-$d_6$) of compound **11**.

**Figure S71** HSQC spectrum of compound **11**.

**Figure S72** HMBC spectrum of compound **11**.

**Figure S73** The chiral HPLC separation and experimental and calculated ECD data of compounds **11a** and **11b**.

**Figure S74** Key HMBC correlations of compounds **11.**

**For compound 12**

**Figure S75** HRESIMS for compound **12**.

**Figure S76** $^{1}$H NMR spectrum (400 MHz, acetone-$d_6$) of compound **12**.

**Figure S77** $^{13}$C NMR spectrum (100 MHz, acetone-$d_6$) of compound **12**.

**Figure S78** HSQC spectrum of compound **12**.

**Figure S79** HMBC spectrum of compound **12**.

**Figure S80** Key HMBC correlations of compounds **12.**

**For compound 13**

**Figure S81** HRESIMS for compound **13**.

**Figure S82** $^{1}$H NMR spectrum (400 MHz, acetone-$d_6$) of compound **13**.

**Figure S83** $^{13}$C NMR spectrum (100 MHz, acetone-$d_6$) of compound **13**.

**Figure S84** HSQC spectrum of compound **13**.

**Figure S85** HMBC spectrum of compound **13**.

**Figure S86** The chiral HPLC separation and experimental and calculated ECD data of compounds **13a** and **13b**.

**Figure S87** Key HMBC correlations of compounds **13.**

**For compound 14**

**Figure S88** HRESIMS for compound **14**.

**Figure S89** $^{1}$H NMR spectrum (500 MHz, acetone-$d_6$) of compound **14**.

**Figure S90** $^{13}$C NMR spectrum (125 MHz, acetone-$d_6$) of compound **14**.

**Figure S91** HSQC spectrum of compound **14**.

**Figure S92** HMBC spectrum of compound **14**.

**Figure S93** The chiral HPLC separation and experimental and calculated ECD data of compounds **14a** and **14b**.
**Figure S94** Key HMBC correlations of compounds **14.**

**For compound 15**

**Figure S95** HRESIMS for compound **15**.
**Figure S96** $^{1}$H NMR spectrum (500 MHz, acetone-$d_6$) of compound **15**.
**Figure S97** $^{13}$C NMR spectrum (125 MHz, acetone-$d_6$) of compound **15**.
**Figure S98** HSQC spectrum of compound **15**.
**Figure S99** HMBC spectrum of compound **15**.
**Figure S100** The chiral HPLC separation and experimental and calculated ECD data of compounds **15a** and **15b**.

**For compound 16**

**Figure S101** HRESIMS for compound **16**.
**Figure S102** $^{1}$H NMR spectrum (500 MHz, acetone-$d_6$) of compound **16**.
**Figure S103** $^{13}$C NMR spectrum (125 MHz, acetone-$d_6$) of compound **16**.
**Figure S104** HSQC spectrum of compound **16**.
**Figure S105** HMBC spectrum of compound **16**.
**Figure S106** The chiral HPLC separation and experimental and calculated ECD data of compounds **16a** and **16b**.
**Figure S107** Key HMBC correlations of compounds **16.**

**For compound 17**

**Figure S108** HRESIMS for compound **17**.
**Figure S109** $^{1}$H NMR spectrum (400 MHz, acetone-$d_6$) of compound **17**.
**Figure S110** $^{13}$C NMR spectrum (100 MHz, acetone-$d_6$) of compound **17**.
**Figure S111** HSQC spectrum of compound **17**.
**Figure S112** HMBC spectrum of compound **17**.

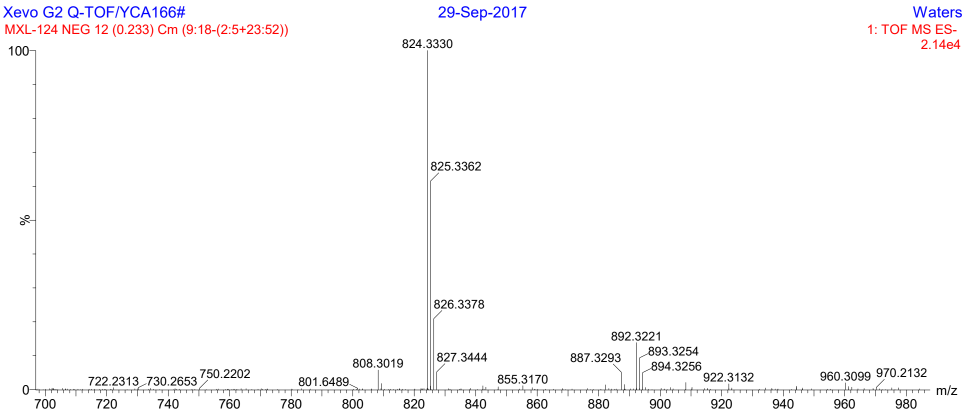


| Mass | Calc. Mass | m*Da* | PPM | *DBE* | i-FIT | Norm | Conf(%) | Formula |
|---|---|---|---|---|---|---|---|---|
| 824.3330 | 824.3336 | -0.6 | -0.7 | 31.5 | 37.5 | 0.567 | 56.71 | C52 H46 N3 O7 |

**Figure S1** HRESIMS for compound **1**.

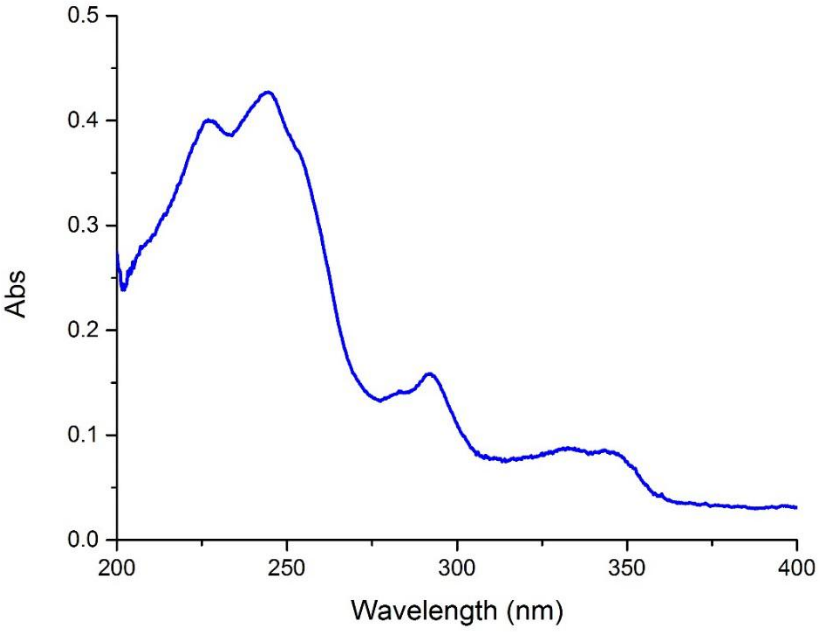


**Figure S2** UV spectrum for compound **1**.

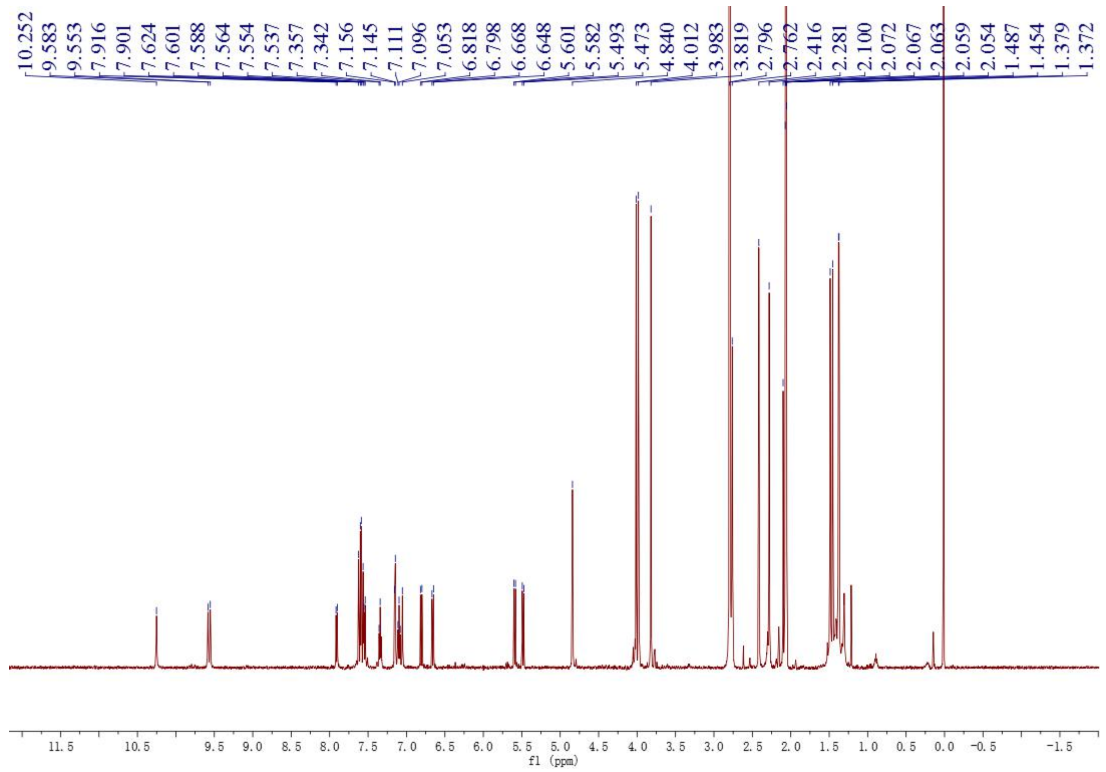


**Figure S3** $^{1}$H NMR spectrum (400 MHz, acetone-$d_6$) of compound **1**.

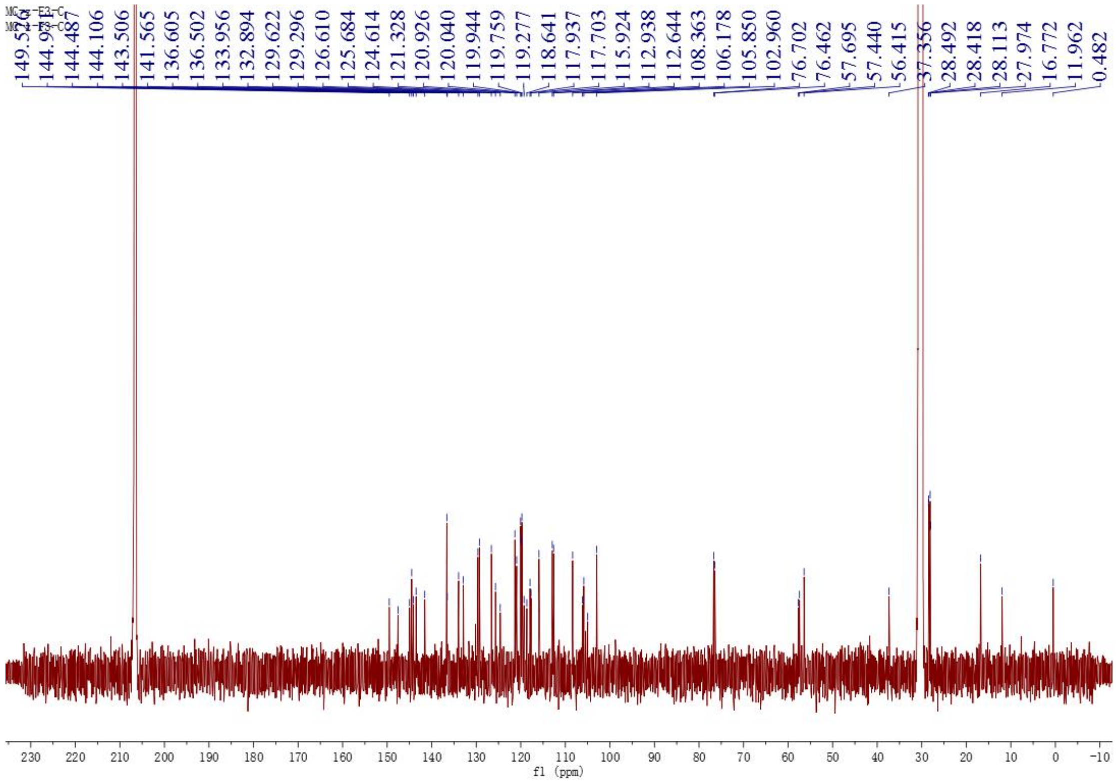


**Figure S4** $^{13}$C NMR spectrum (100 MHz, acetone-$d_6$) of compound **1**.

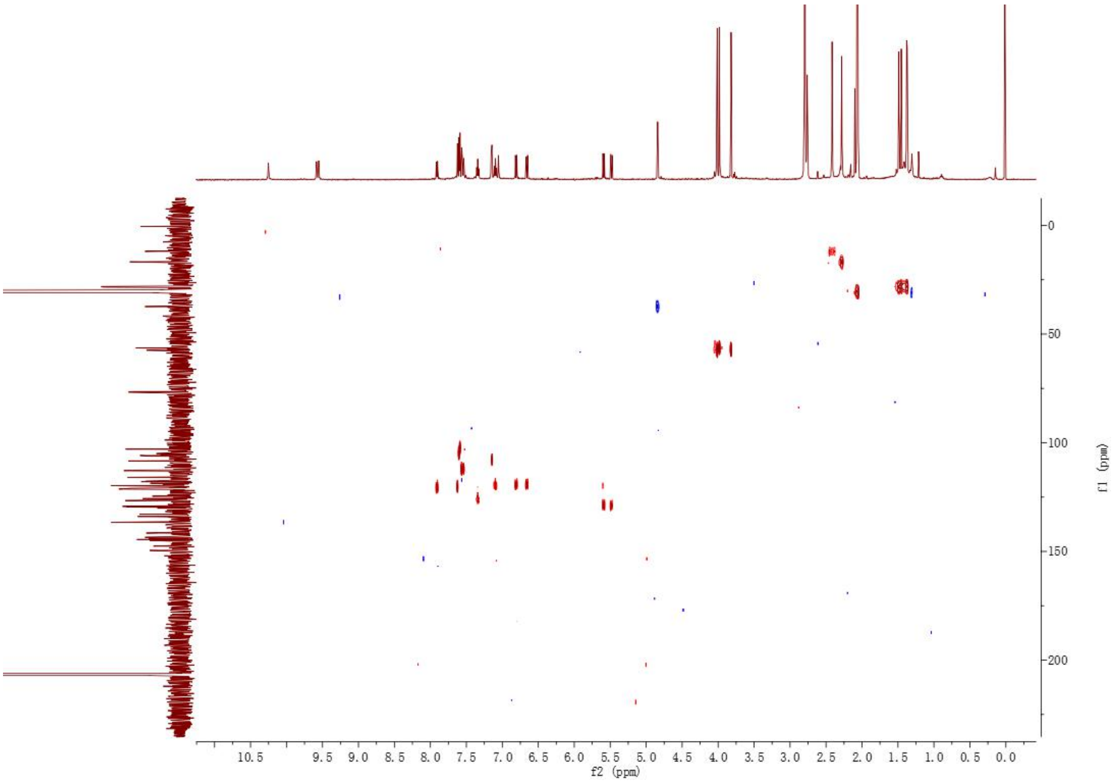


**Figure S5** HSQC spectrum of compound **1**.

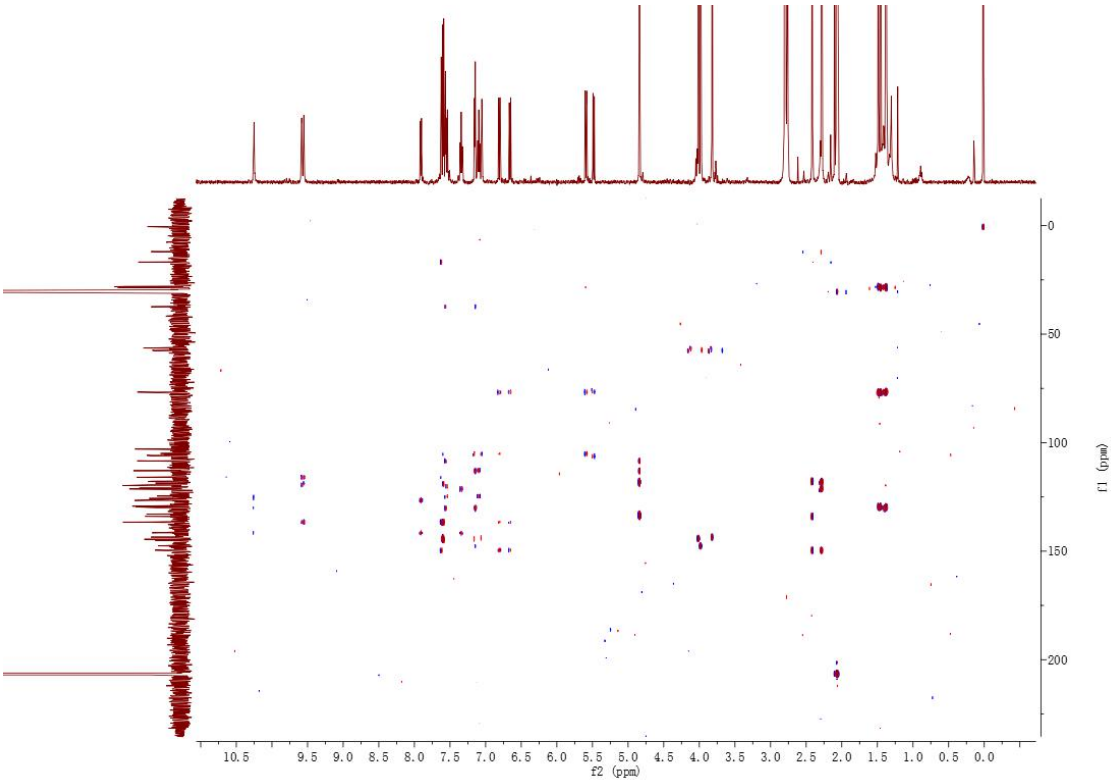


**Figure S6** HMBC spectrum of compound **1**.

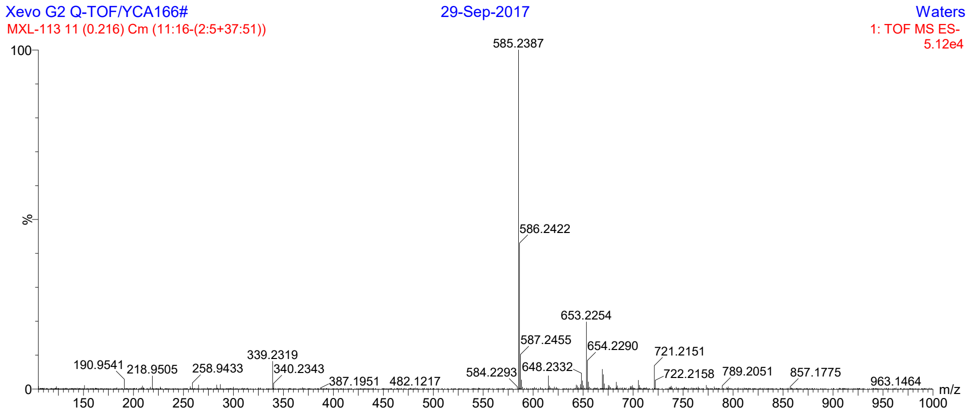


| Mass | Calc. Mass | m*Da* | PPM | *DBE* | i-FIT | Norm | Conf(%) | Formula |
|---|---|---|---|---|---|---|---|---|
| 585.2387 | 585.2389 | -0.2 | -0.3 | 22.5 | 86.8 | 0.000 | 99.99 | C37 H33 N2 O5 |

**Figure S7** HRESIMS for compound **2**

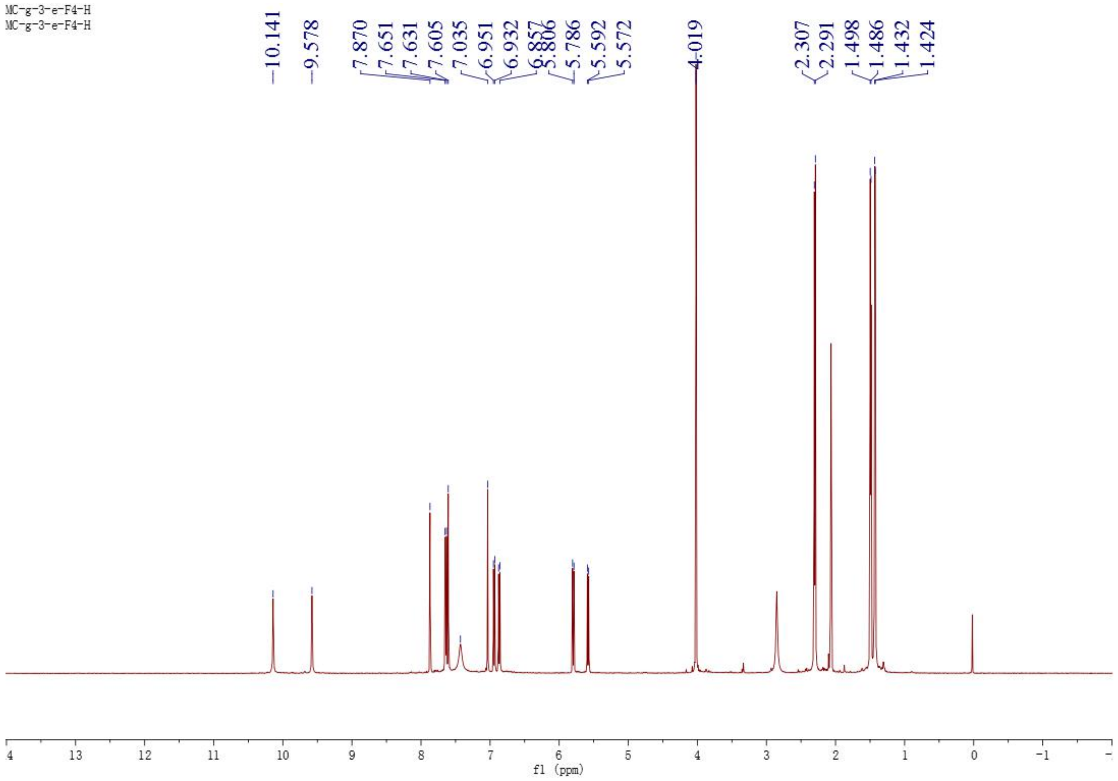


**Figure S8** $^{1}$H NMR spectrum (400 MHz, acetone-$d_6$) of compound **2**.

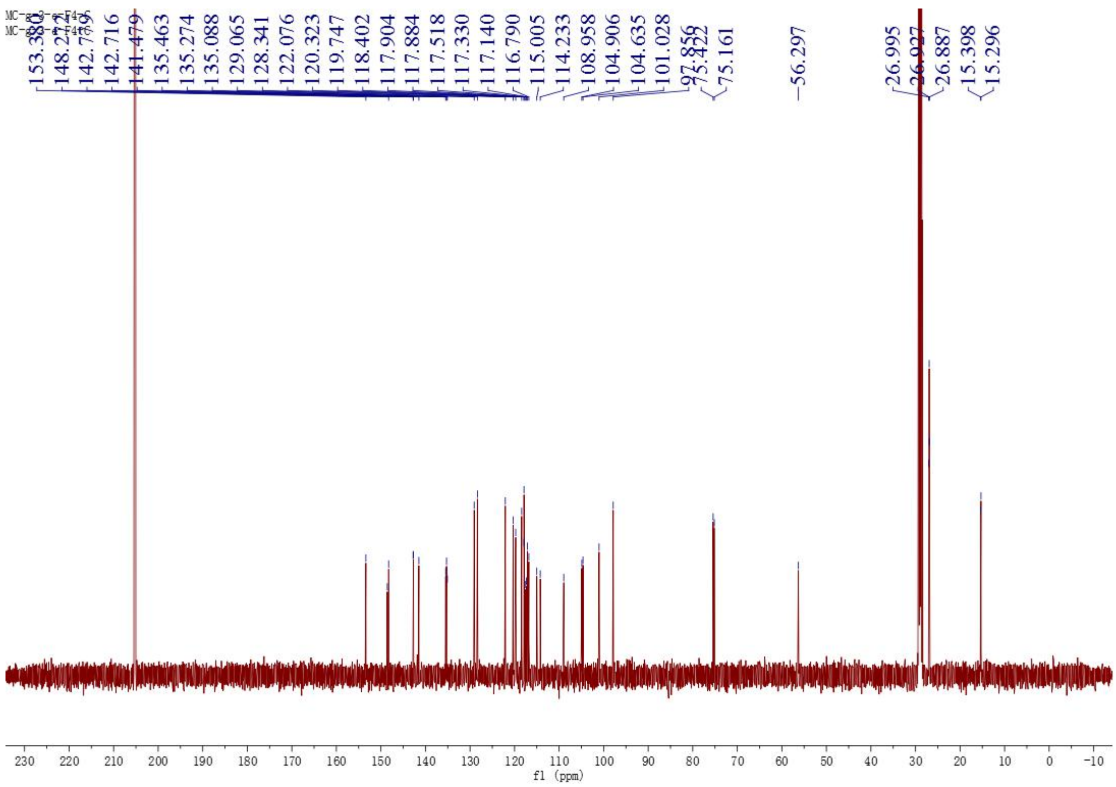


**Figure S9** $^{13}C$ NMR spectrum (100 MHz, acetone-$d_6$) of compound **2**.

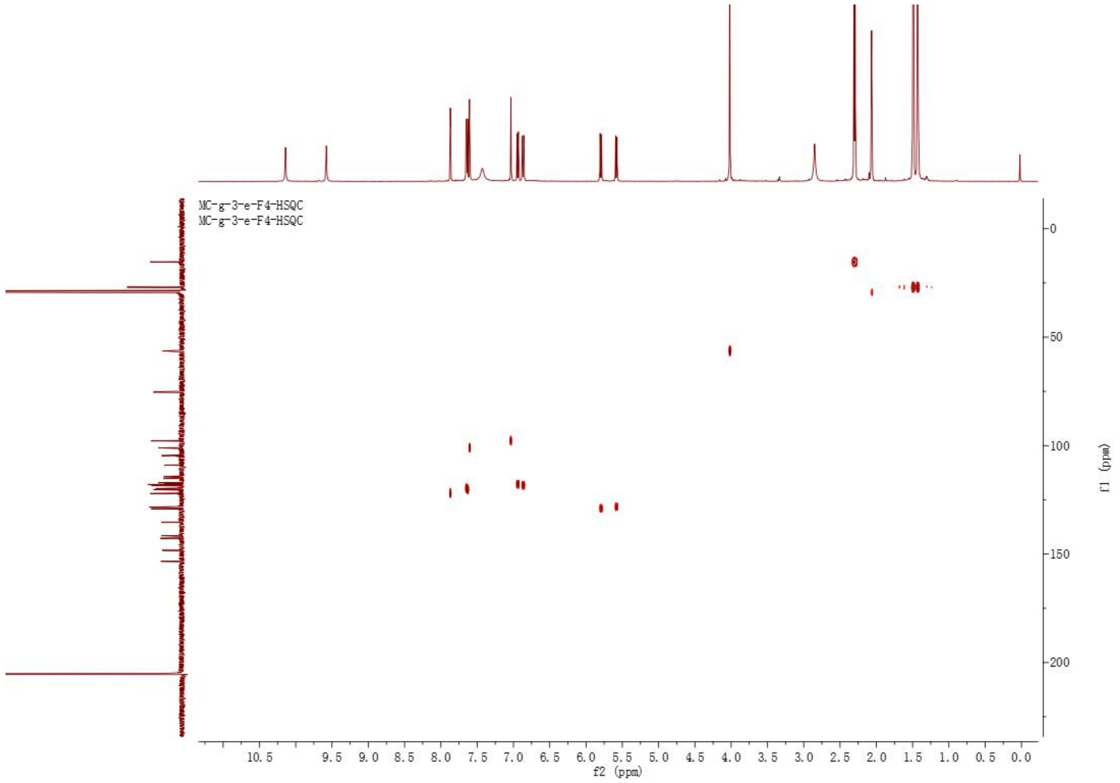


**Figure S10** HSQC spectrum of compound **2**.

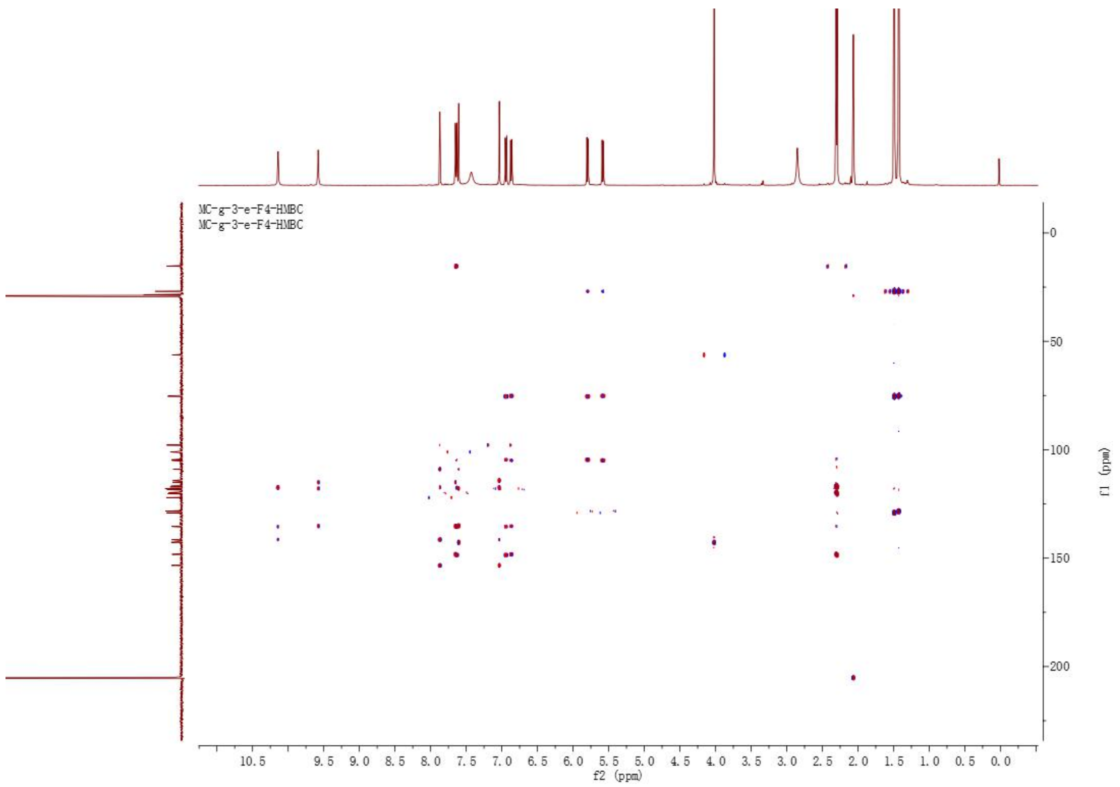


**Figure S11** HMBC spectrum of compound **2**.

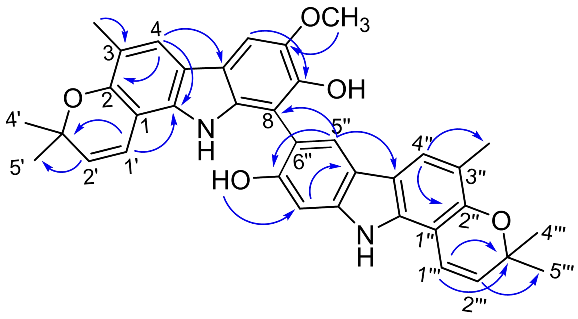


**Figure S12** Key HMBC correlations of compounds **2.**

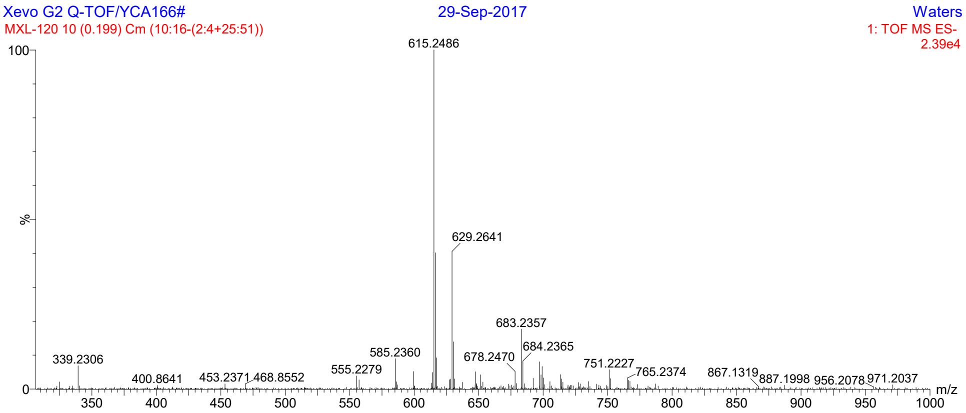


| Mass | Calc. Mass | m*Da* | PPM | *D*BE | i-FIT | Norm | Conf(%) | Formula |
|---|---|---|---|---|---|---|---|---|
| 615.2486 | 615.2495 | -0.9 | -1.5 | 22.5 | 52.8 | 0.528 | 58.99 | C38 H35 N2 O6 |

**Figure S13** HRESIMS for compound **3**

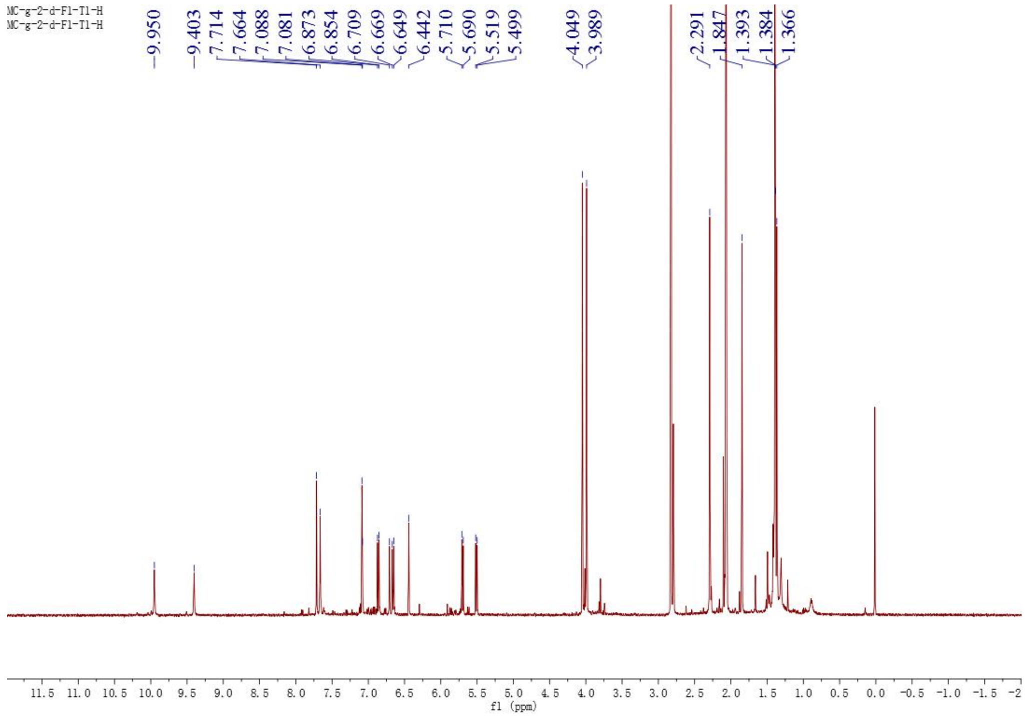


**Figure S14** $^{1}$H NMR spectrum (400 MHz, acetone-$d_6$) of compound **3**.

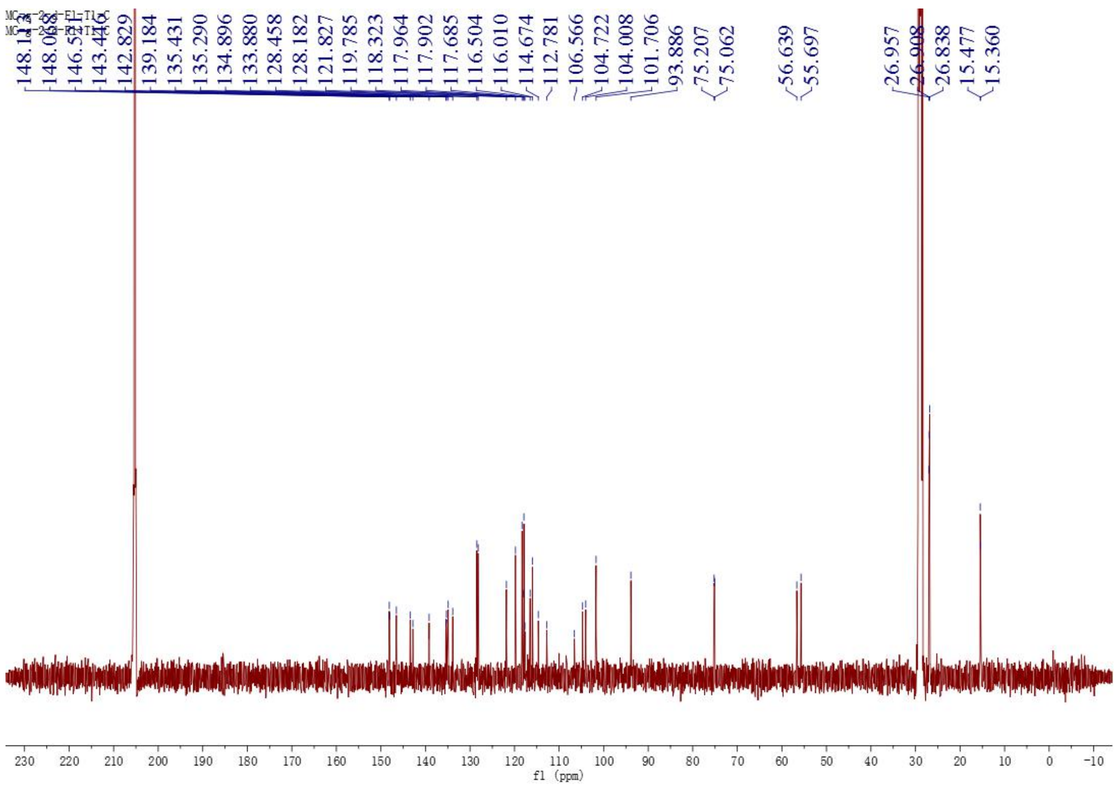


**Figure S15** $^{13}$C NMR spectrum (100 MHz, acetone-$d_6$) of compound **3**.

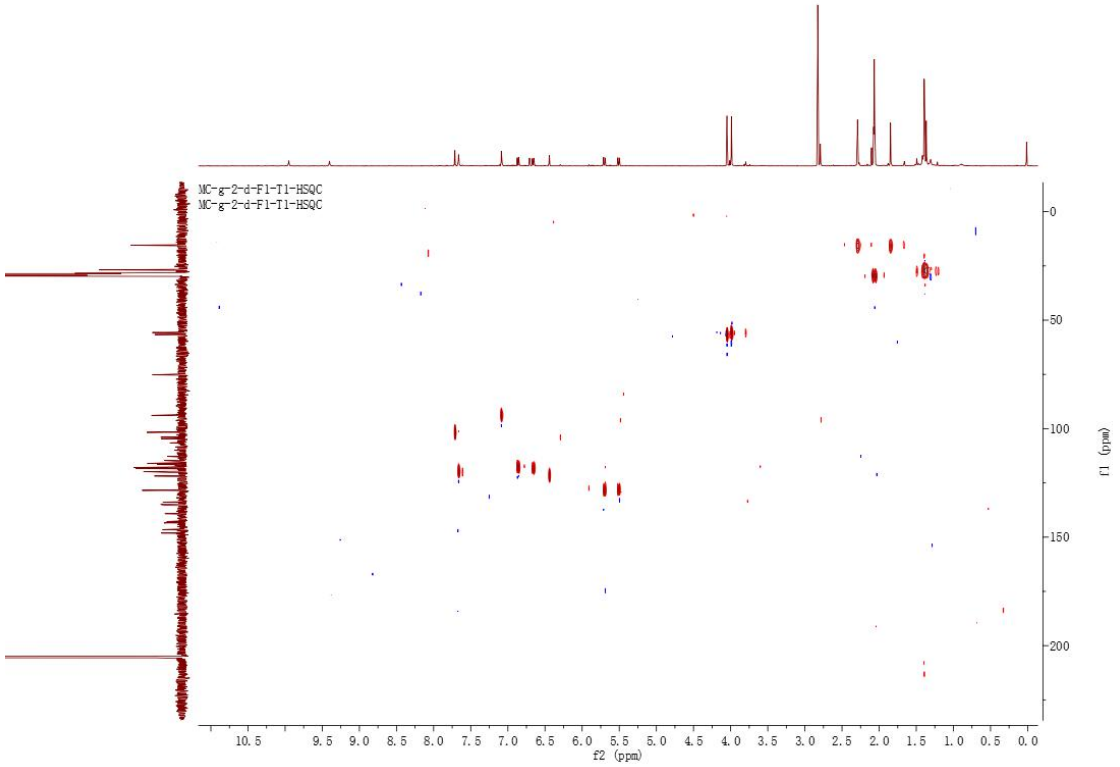


**Figure S16** HSQC spectrum of compound **3**.

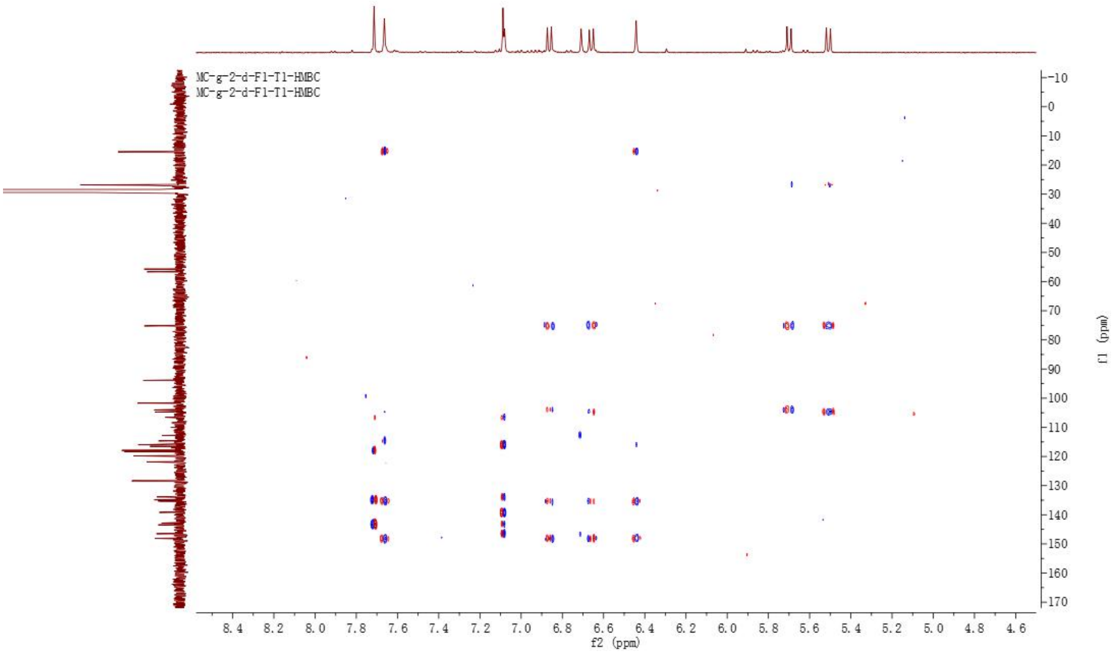


**Figure S17** HMBC spectrum of compound **3**.

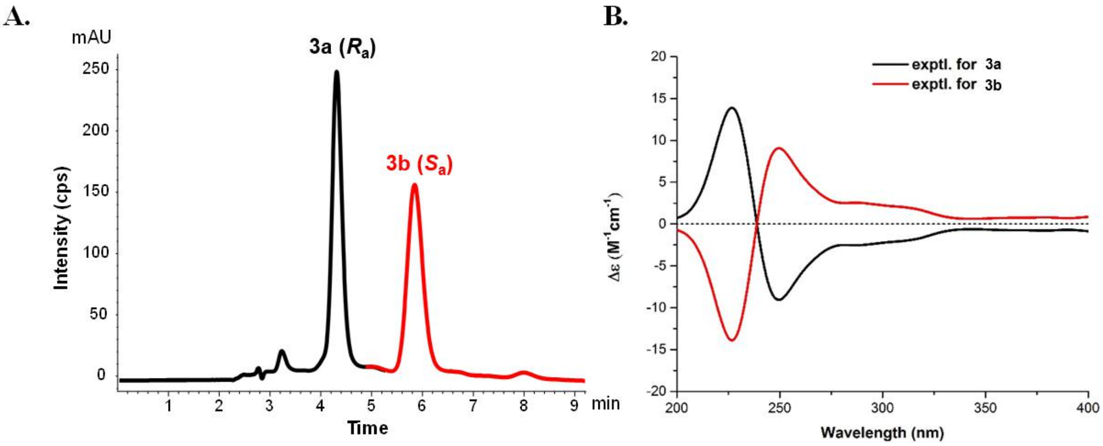


**Figure S18** The chiral HPLC separation (A) and experimental ECD data (B) of compounds **3a** and **3b**.

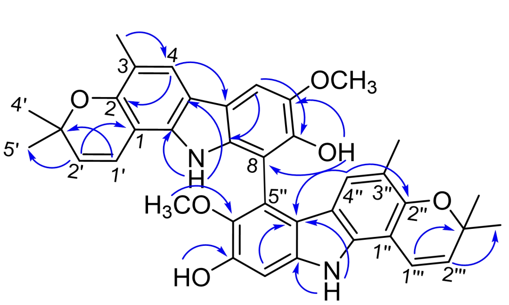

**Figure S19** Key HMBC correlations of compounds **3.**

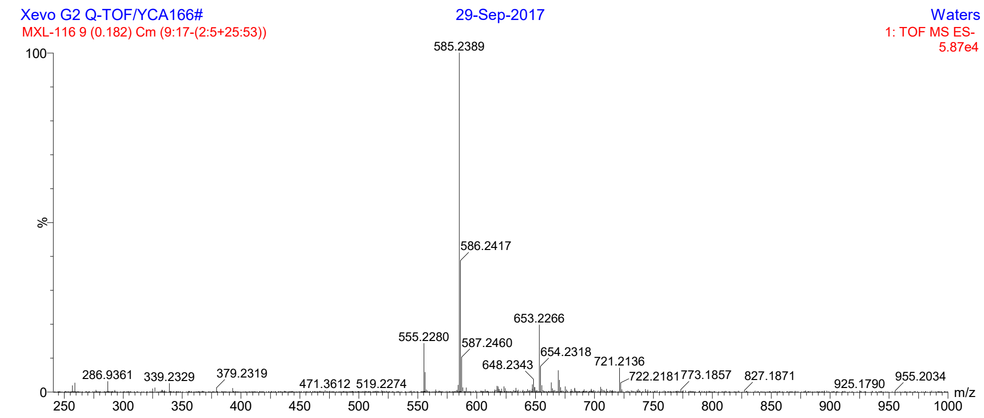


| Mass | Calc. Mass | m*Da* | PPM | *D*BE | i-FIT | Norm | Conf(%) | Formula |
|---|---|---|---|---|---|---|---|---|
| 585.2389 | 585.2389 | 0.0 | 0.0 | 22.5 | 92.1 | 0.001 | 99.91 | C37 H33 N2 O5 |

**Figure S20** HRESIMS for compound **4**

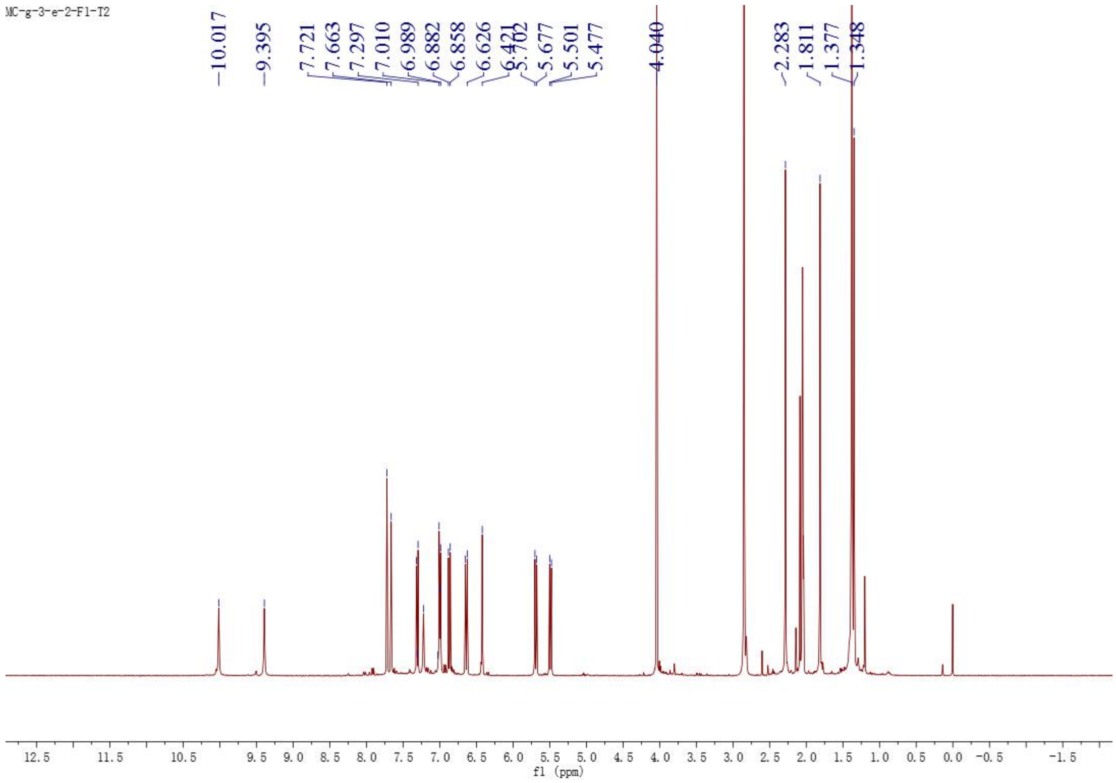


**Figure S21** $^{1}$H NMR spectrum (400 MHz, acetone-$d_6$) of compound **4**.

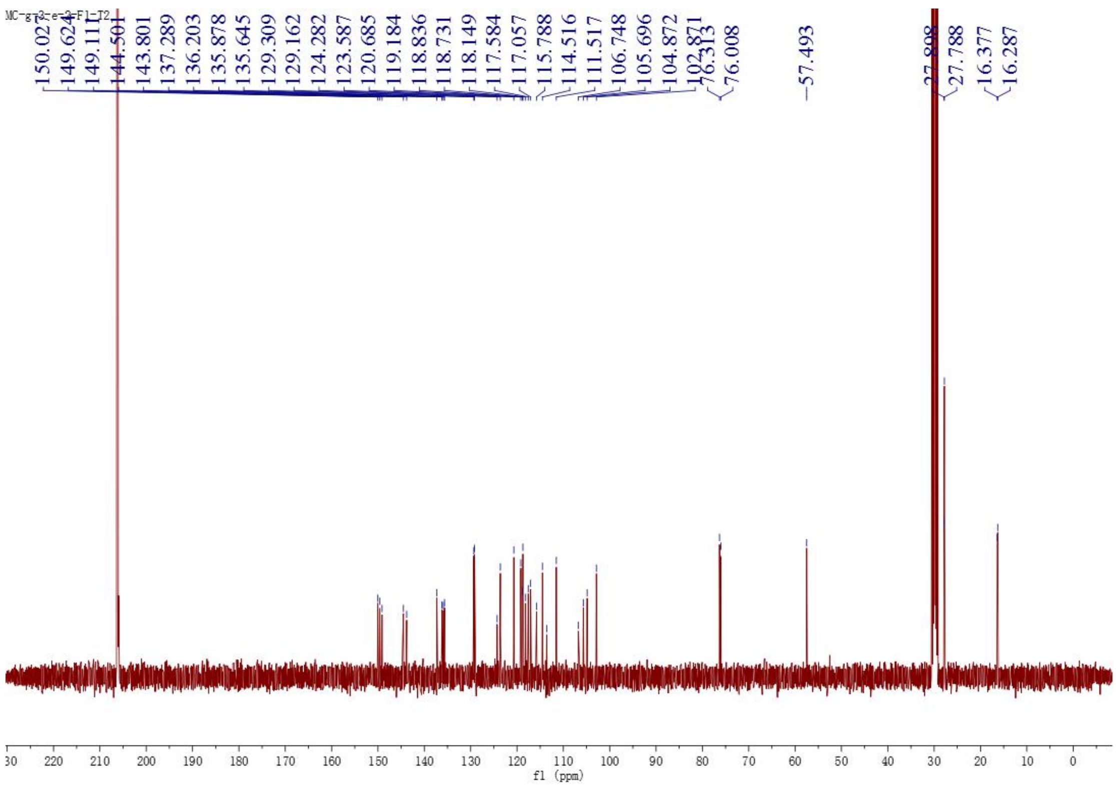


**Figure S22** $^{13}$C NMR spectrum (100 MHz, acetone-$d_6$) of compound **4**.

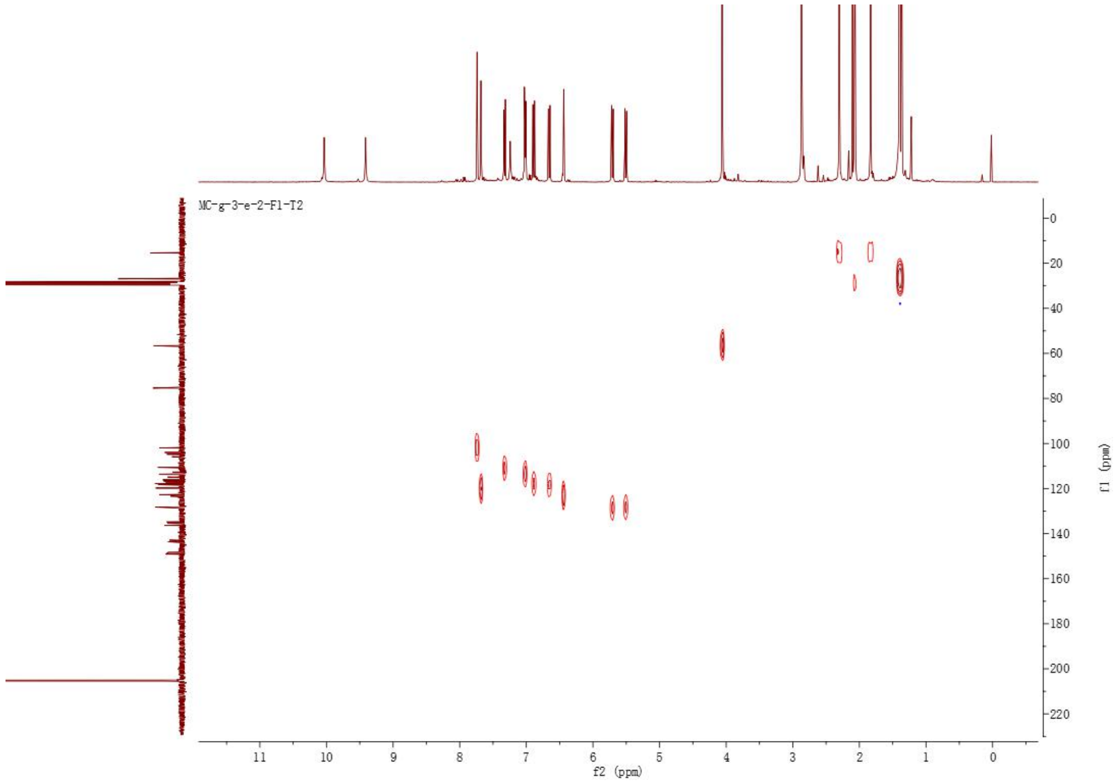


**Figure S23** HSQC spectrum of compound **4**.

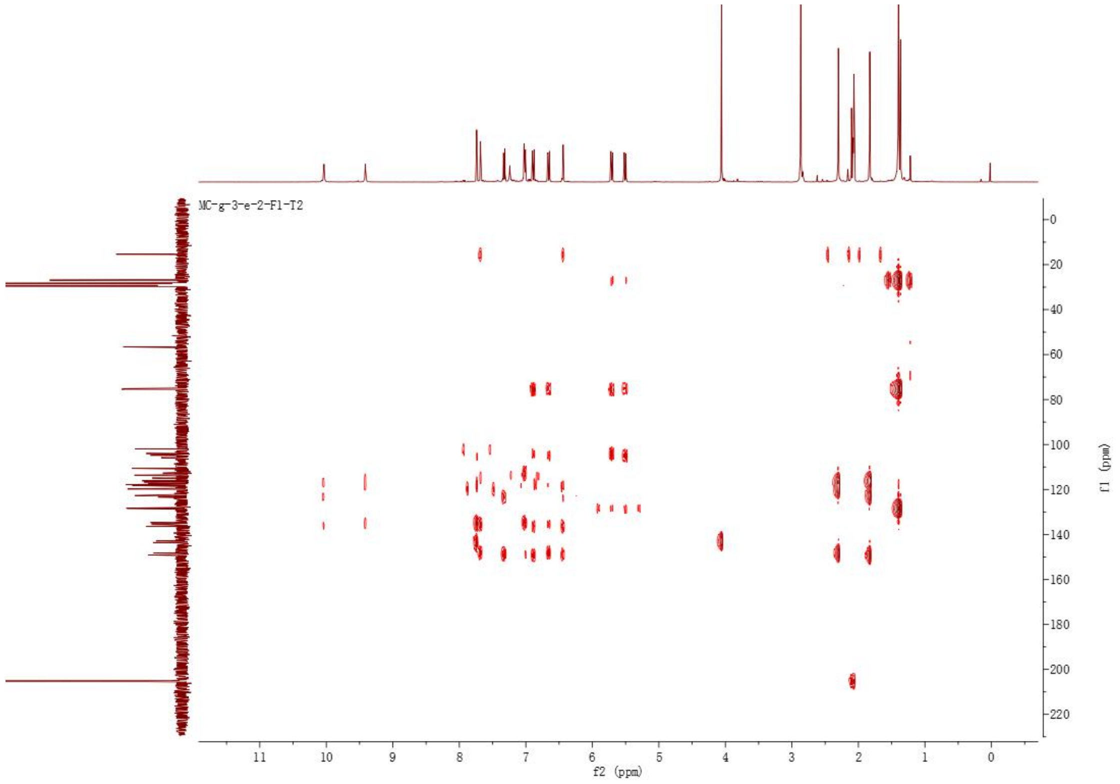


**Figure S24** HMBC spectrum of compound **4**.

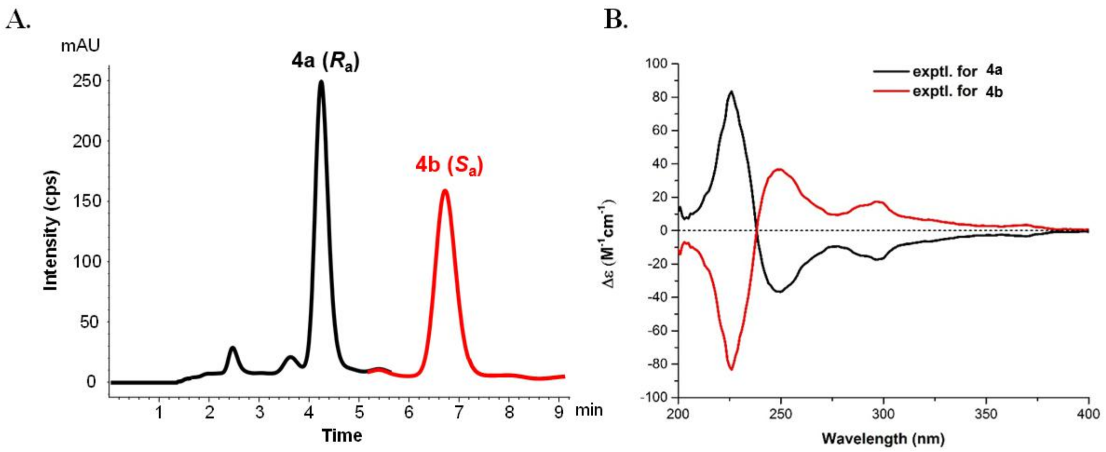


**Figure S25** The chiral HPLC separation (A) and experimental ECD data (B) of compounds **4a** and **4b**.

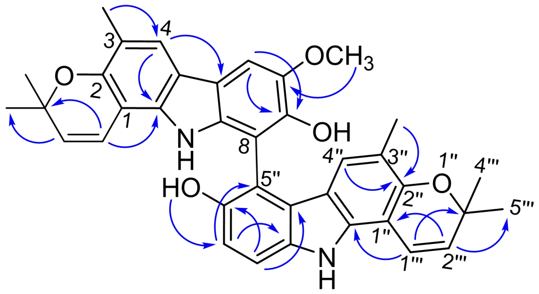

**Figure S26** Key HMBC correlations of compounds **4.**

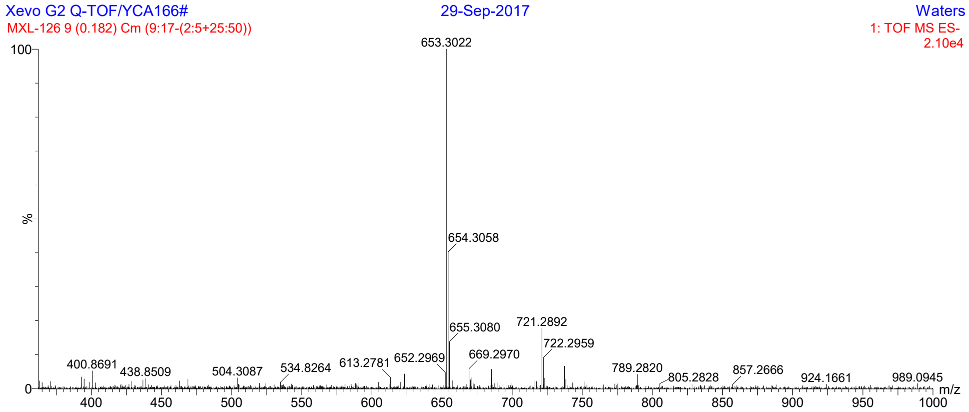


| Mass | Calc. Mass | m*Da* | PPM | *DBE* | i-FIT | Norm | Conf(%) | Formula |
|---|---|---|---|---|---|---|---|---|
| 653.3022 | 653.3015 | 0.7 | 1.1 | 23.5 | 104.5 | 0.766 | 46.49 | C42 H41 N2 O5 |

**Figure S27** HRESIMS for compound **5**.

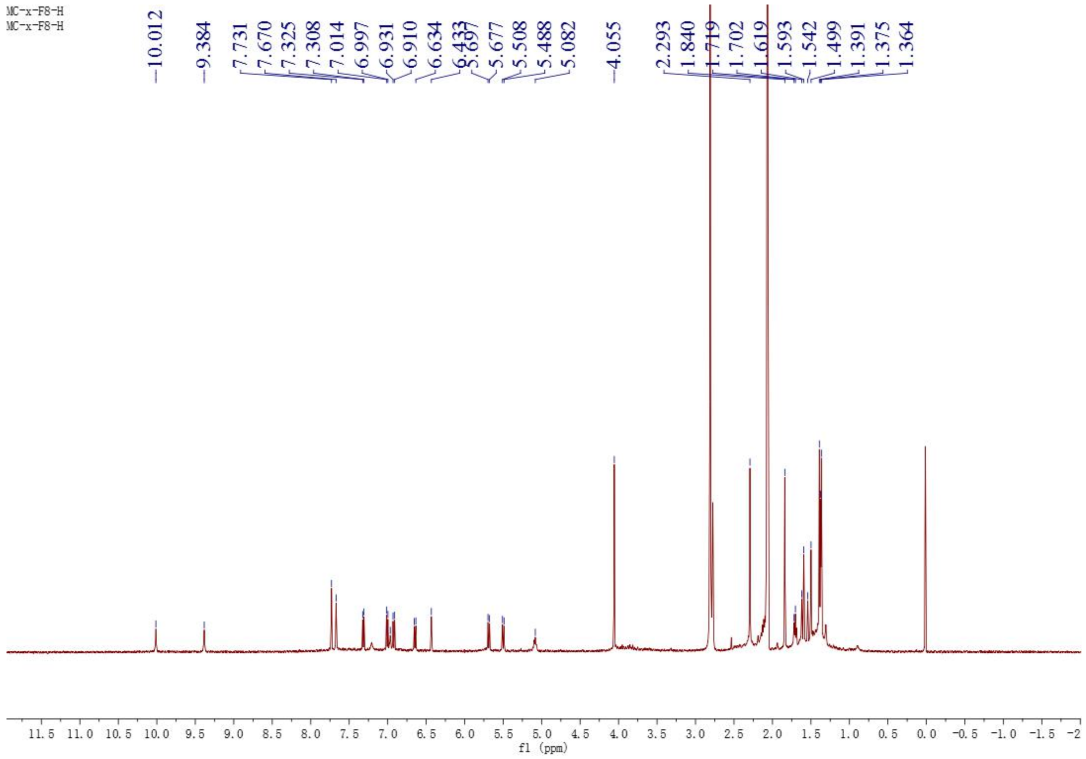


**Figure S28** $^{1}$H NMR spectrum (500 MHz, acetone-$d_6$) of compound **5**.

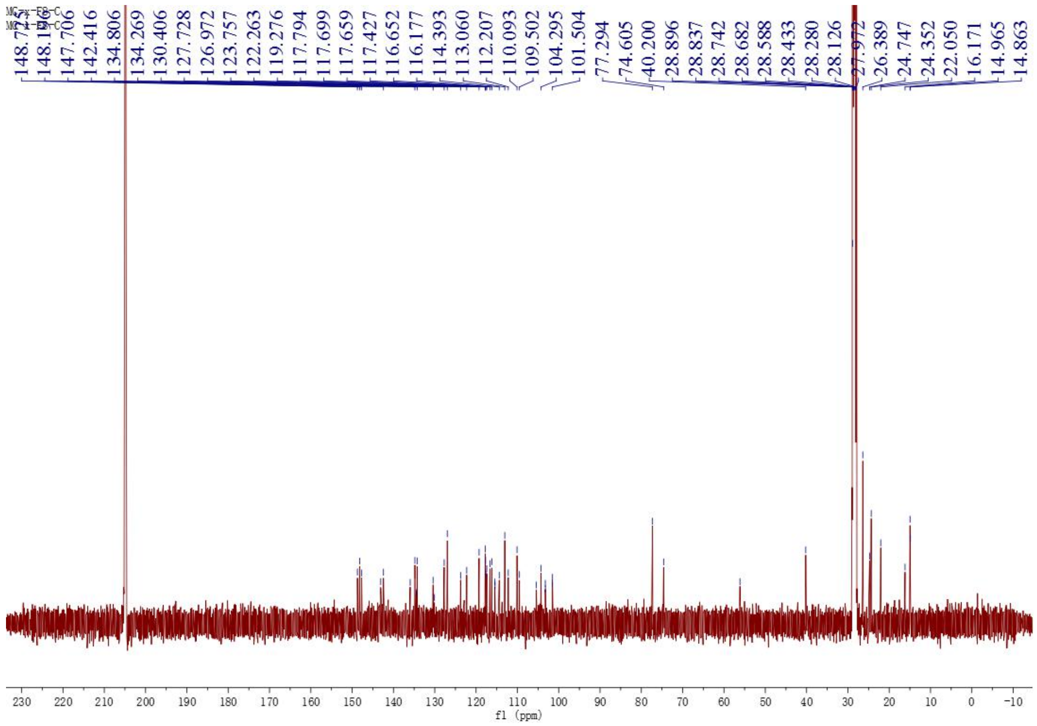


**Figure S29** $^{13}$C NMR spectrum (125 MHz, acetone-$d_6$) of compound **5**.

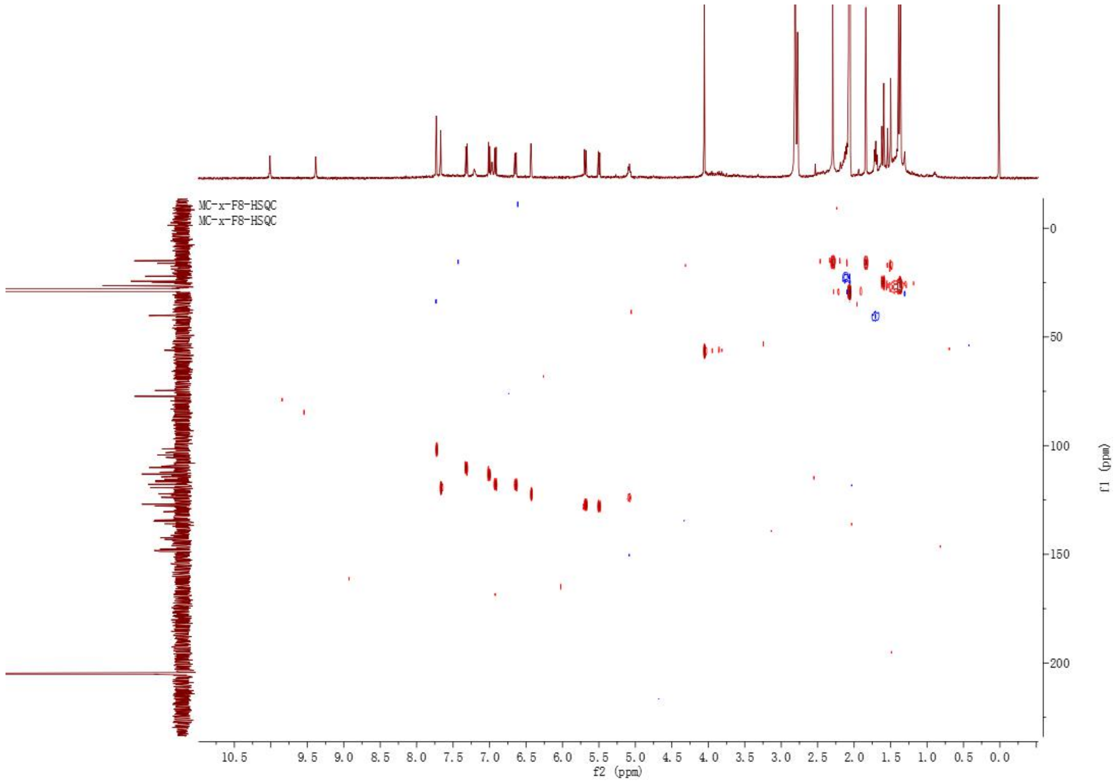


**Figure S30** HSQC spectrum of compound **5**.

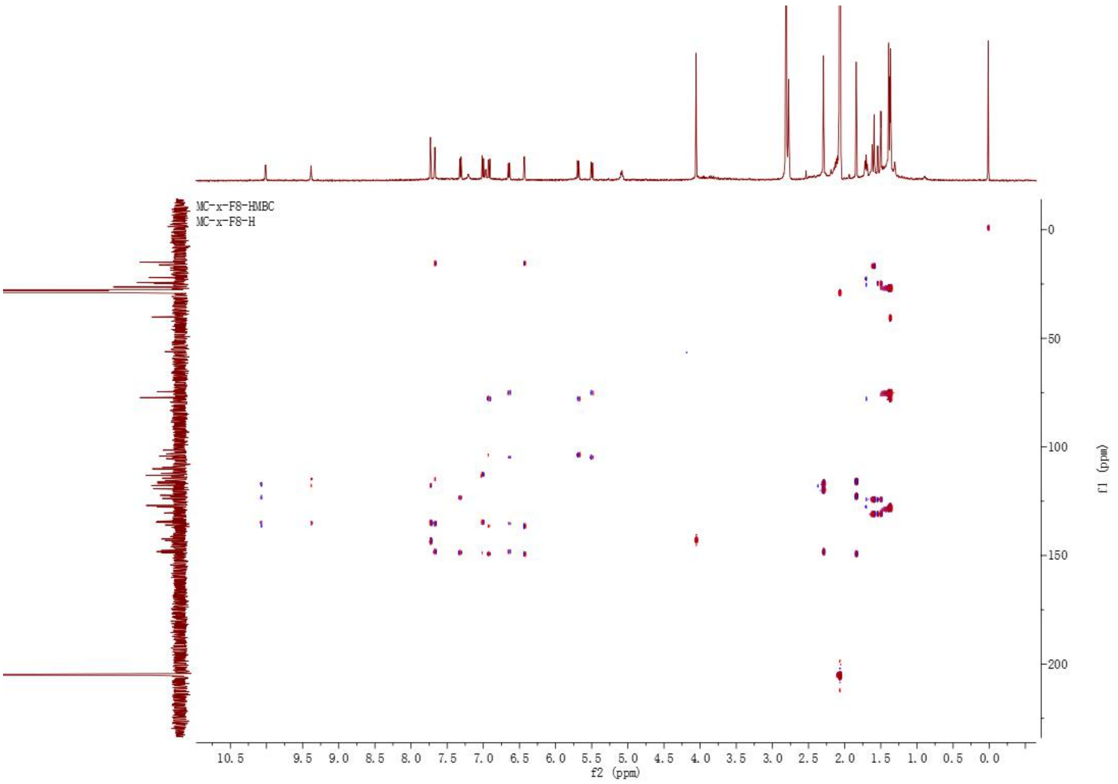


**Figure S31** HMBC spectrum of compound **5**.

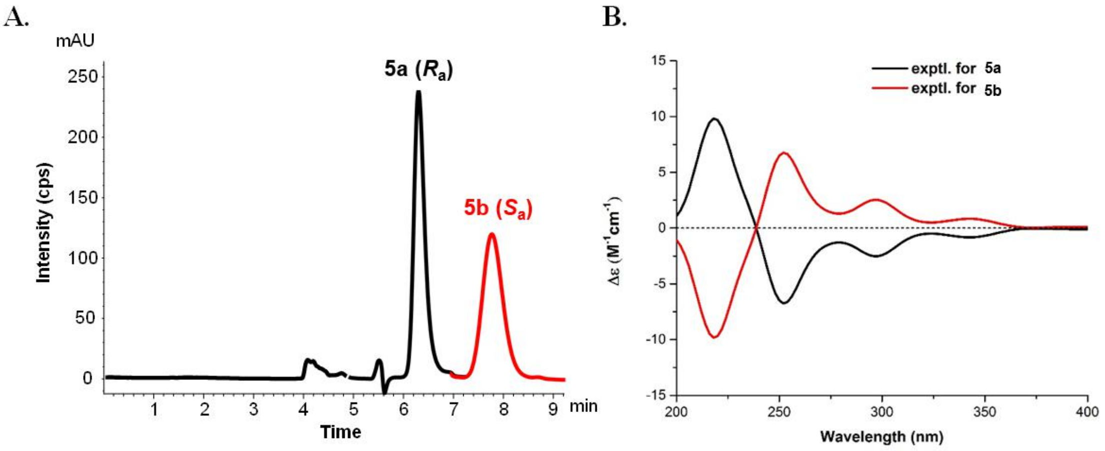


**Figure S32** The chiral HPLC separation (A) and experimental ECD data (B) of compounds **5a** and **5b**.

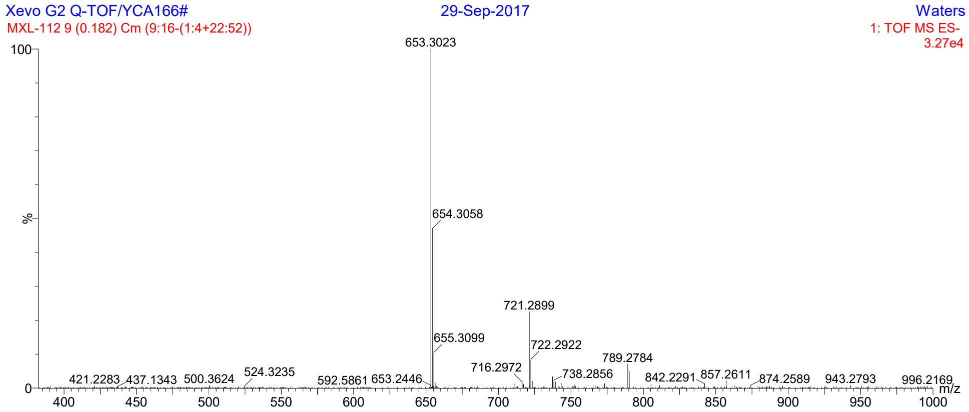


| Mass | Calc. Mass | m*Da* | PPM | *DBE* | i-FIT | Norm | Conf(%) | Formula |
|---|---|---|---|---|---|---|---|---|
| 653.3023 | 653.3015 | 0.8 | 1.2 | 23.5 | 92.7 | 0.740 | 47.69 | C42 H41 N2 O5 |

**Figure S33** HRESIMS for compound **6**.

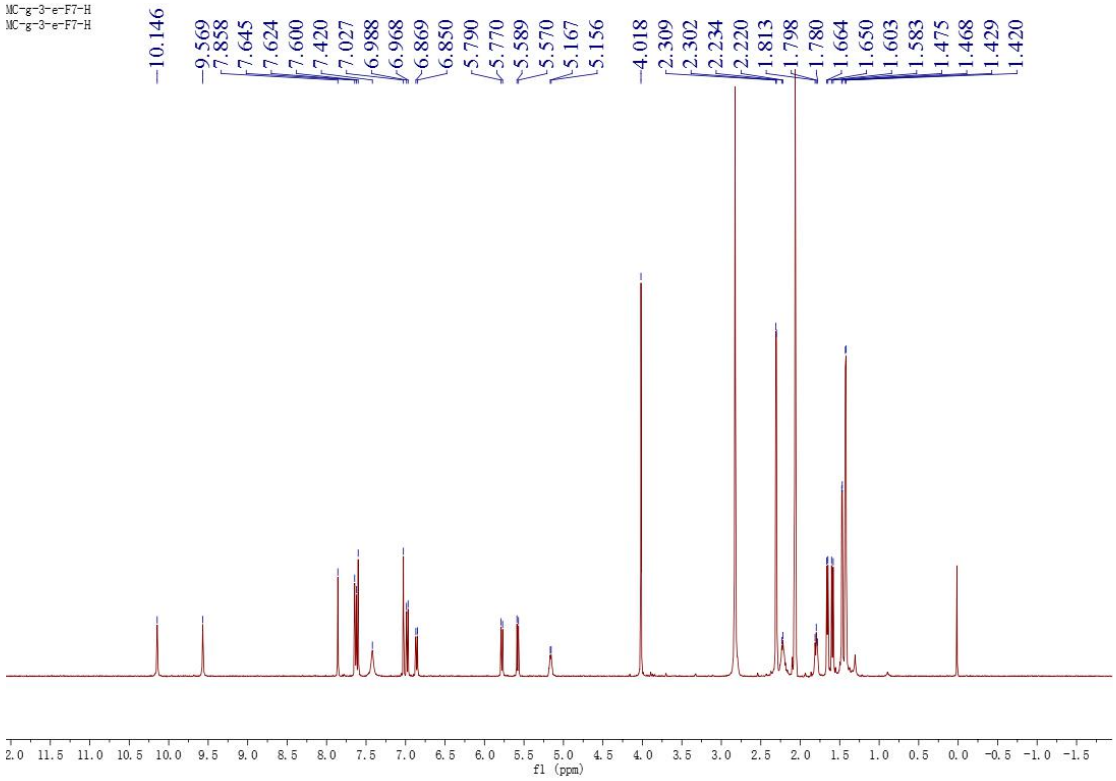


**Figure S34** $^{1}$H NMR spectrum (500 MHz, acetone-$d_6$) of compound **6**.

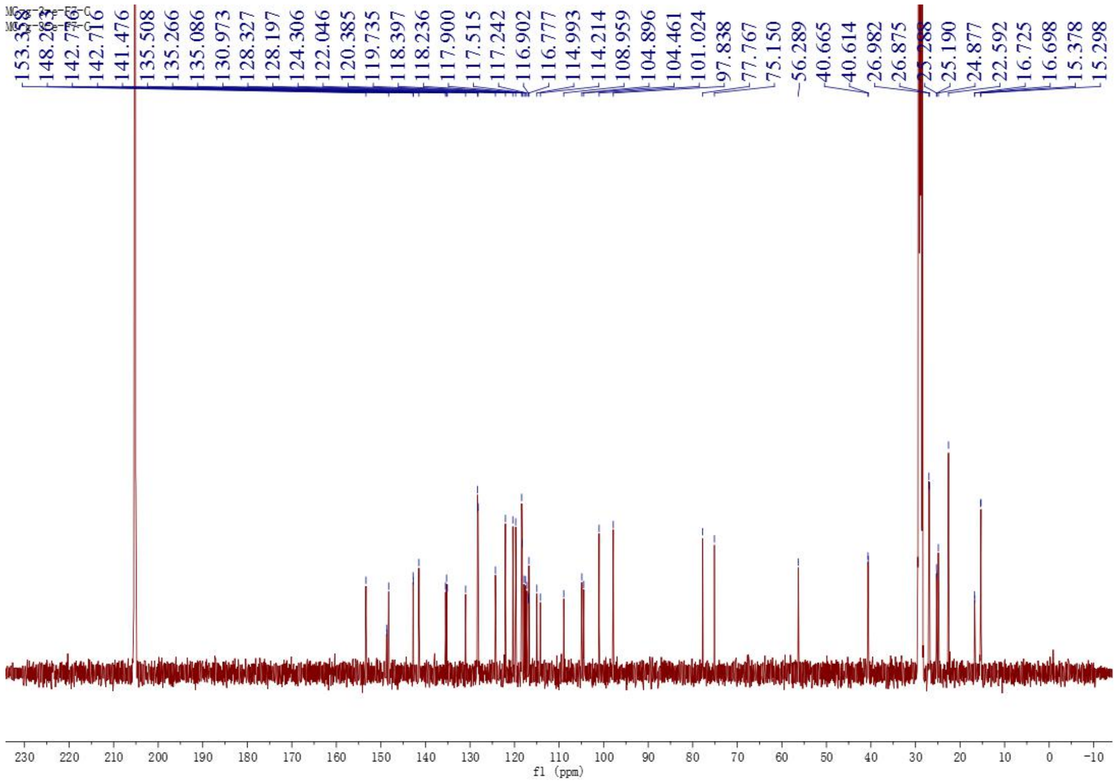


**Figure S35** $^{13}$C NMR spectrum (125 MHz, acetone-$d_6$) of compound **6**.

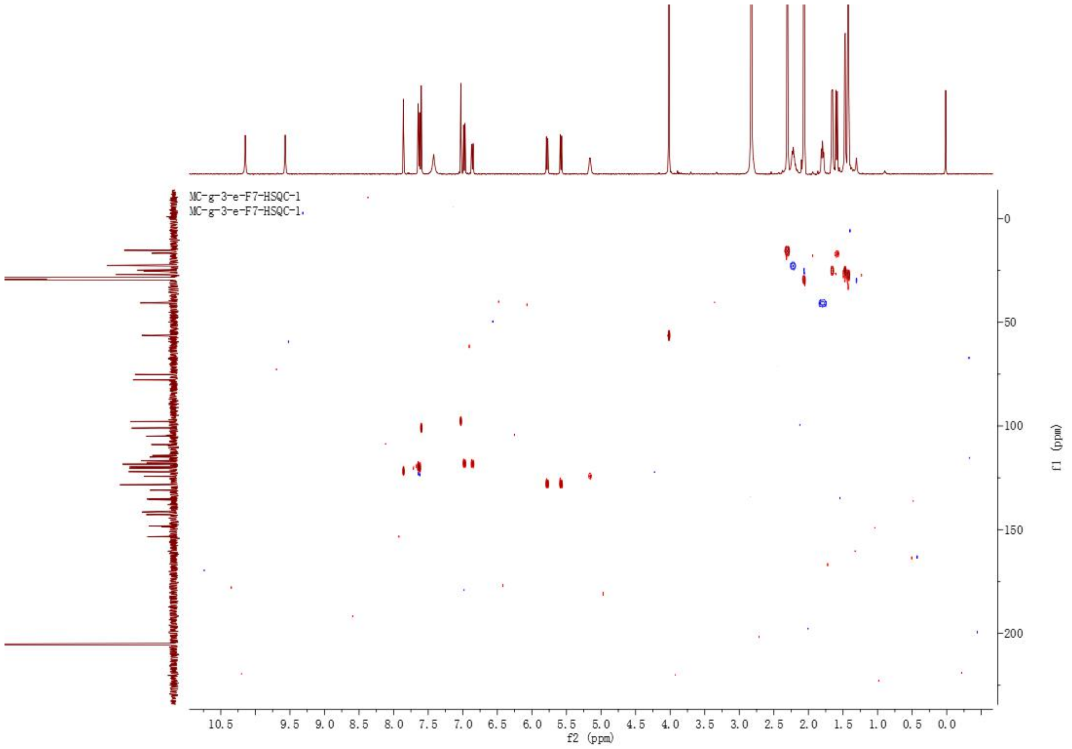


**Figure S36** HSQC spectrum of compound **6**.

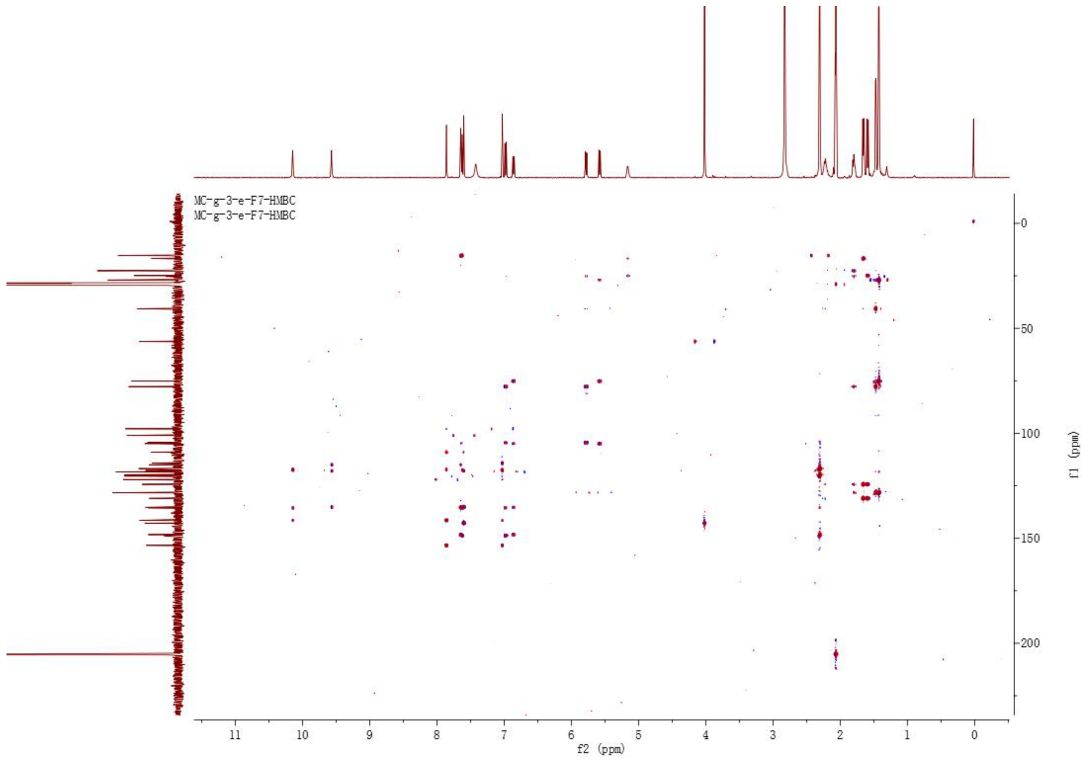


**Figure S37** HMBC spectrum of compound **6**.

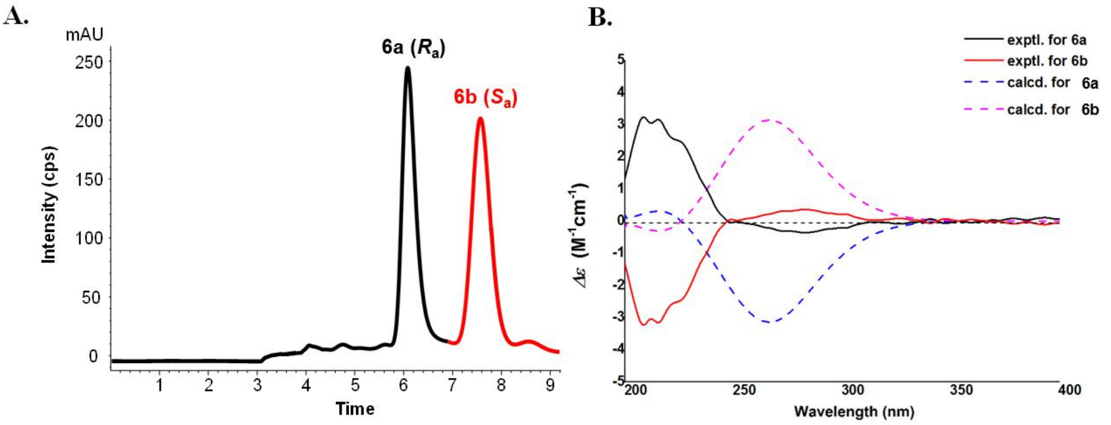


**Figure S38** The chiral HPLC separation (A) and experimental and calculated ECD data (B) of compounds **6a** and **6b**.

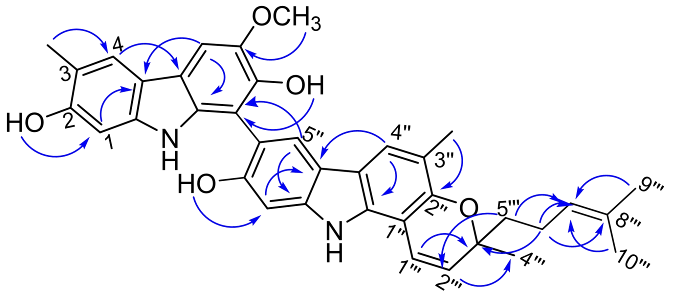


**Figure S39** Key HMBC correlations of compounds **6.**

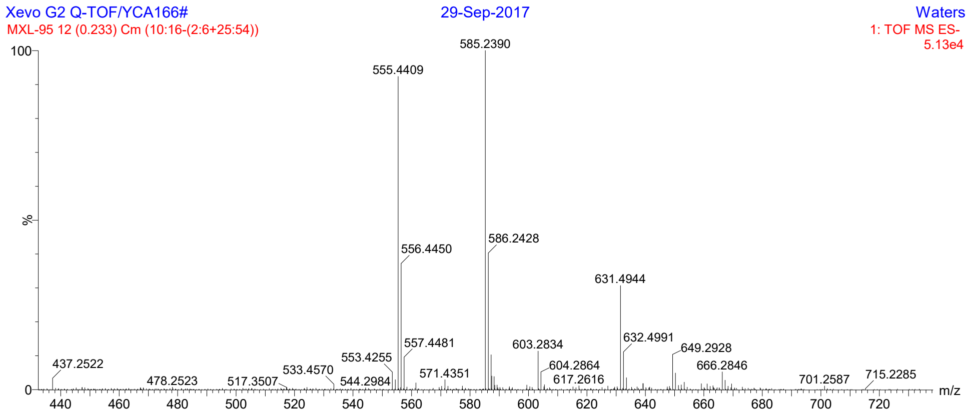


| Mass | Calc. Mass | m*Da* | PPM | *DBE* | i-FIT | Norm | Conf(%) | Formula |
|---|---|---|---|---|---|---|---|---|
| 585.2390 | 585.2389 | 0.1 | 0.2 | 22.5 | 127.1 | 0.000 | 99.98 | C37 H33 N2 O5 |

**Figure S40** HRESIMS for compound **7**.

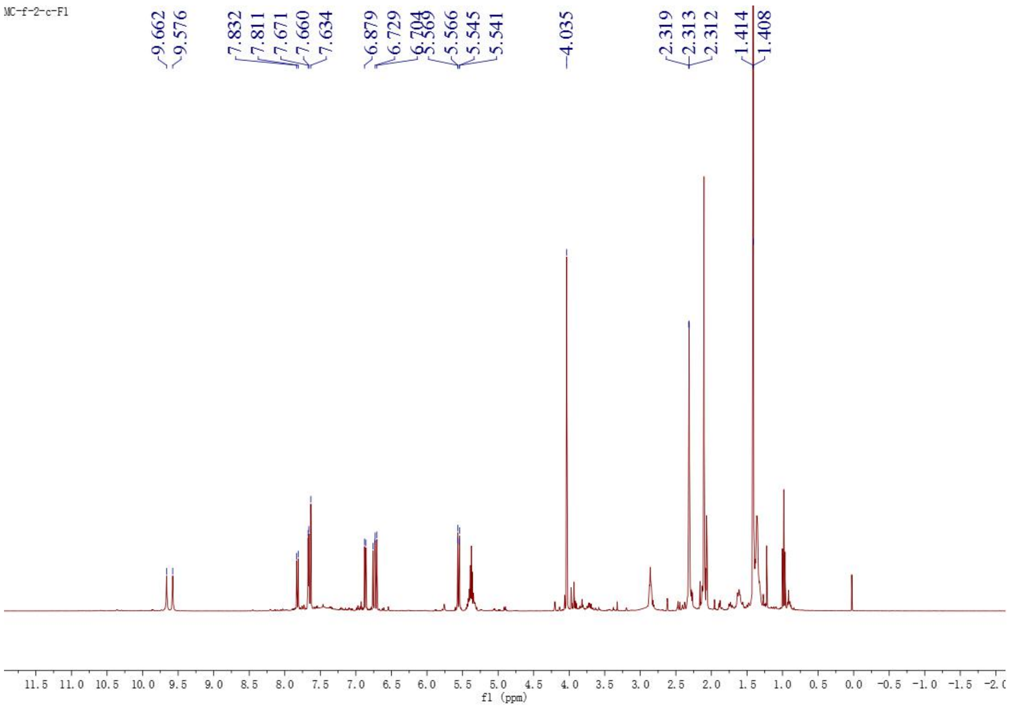


**Figure S41** $^{1}$H NMR spectrum (400 MHz, acetone-$d_6$) of compound **7**.

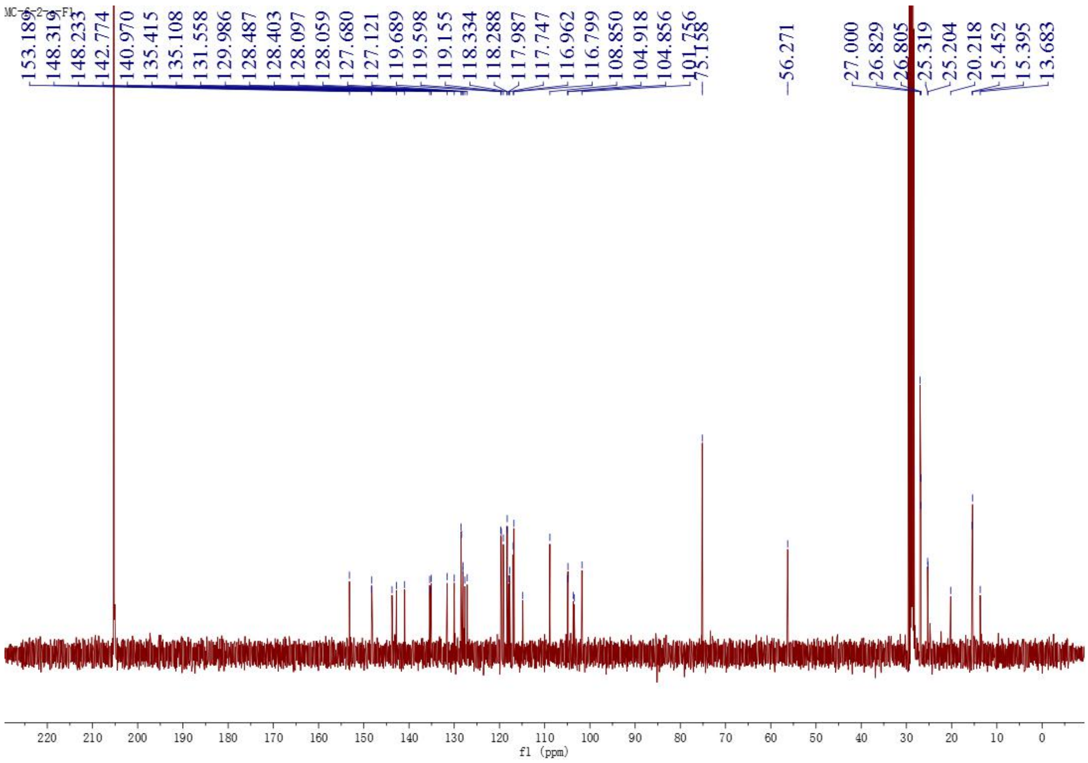


**Figure S42** $^{13}$C NMR spectrum (100 MHz, acetone-$d_6$) of compound **7**.

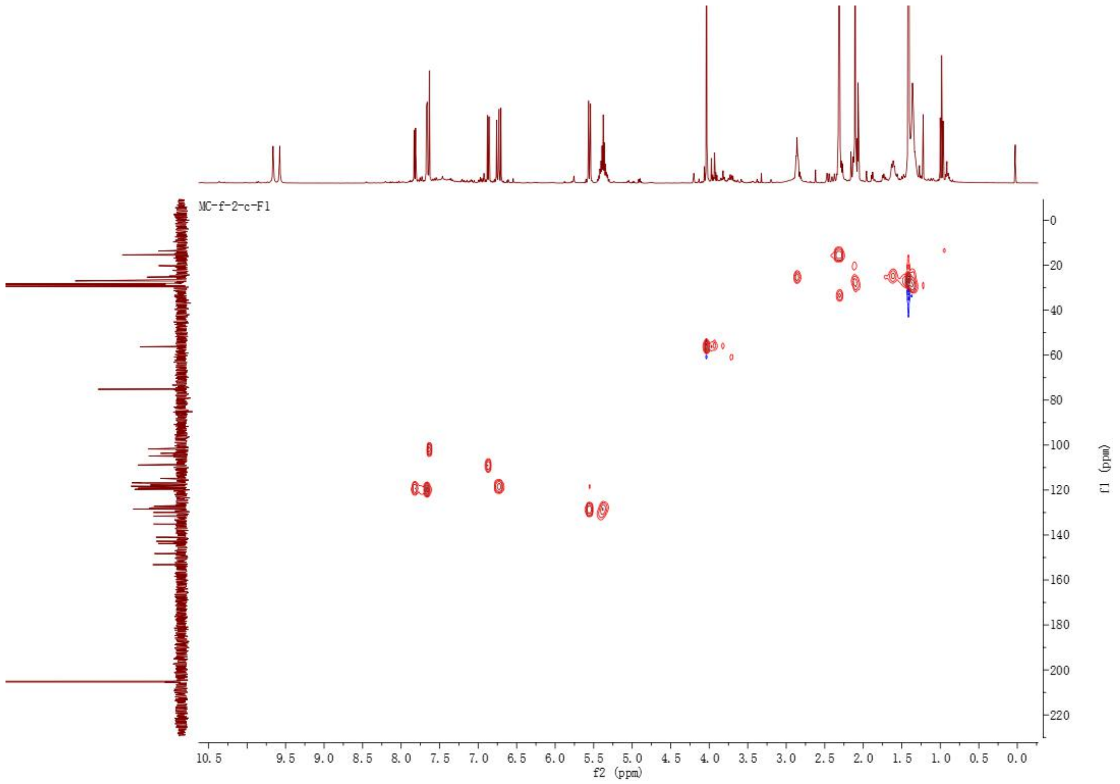


**Figure S43** HSQC spectrum of compound **7**.

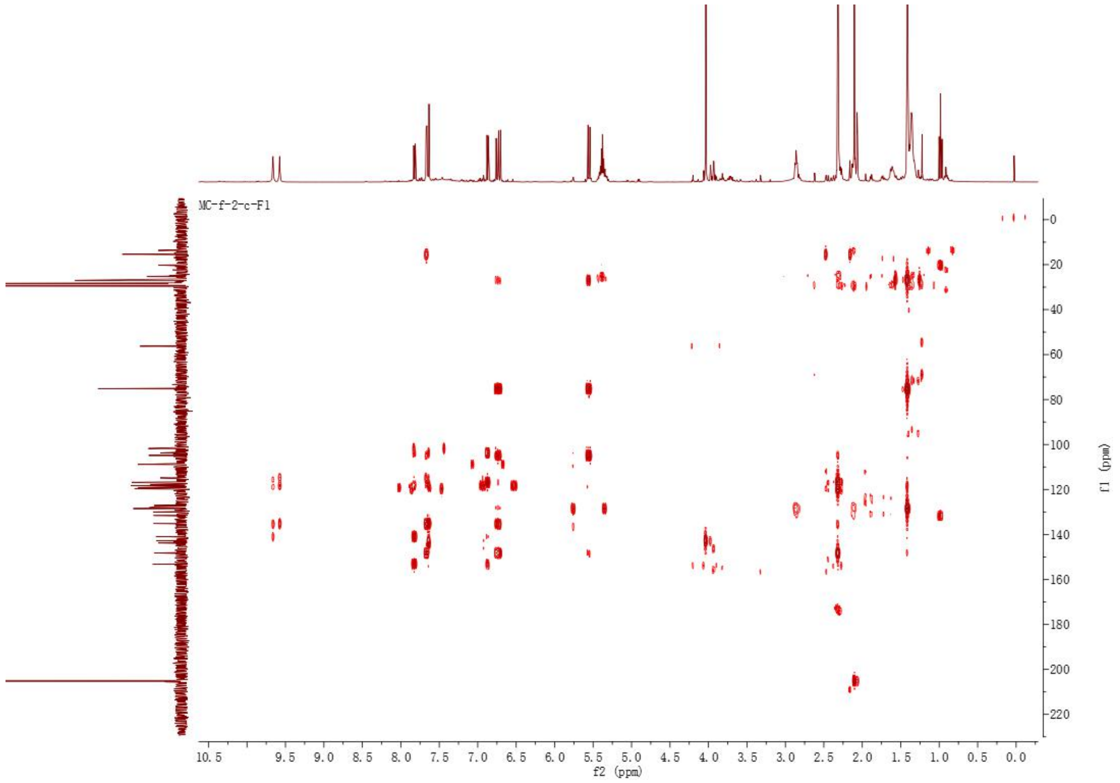


**Figure S44** HMBC spectrum of compound **7**.

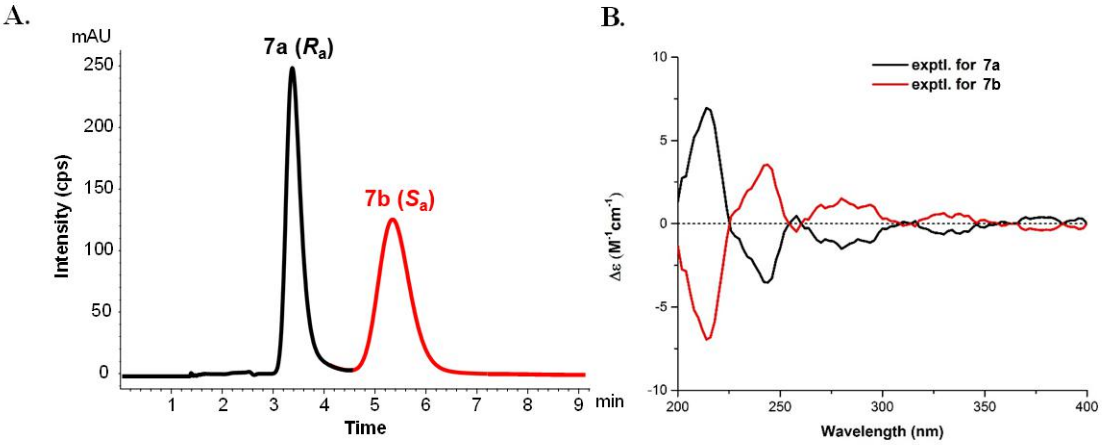


**Figure S45** The chiral HPLC separation (A) and experimental ECD data (B) of compounds **7a** and **7b**.

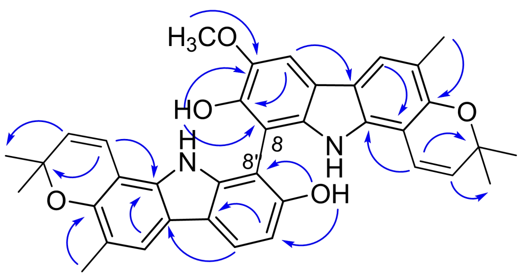


**Figure S46** Key HMBC correlations of compound **7.**

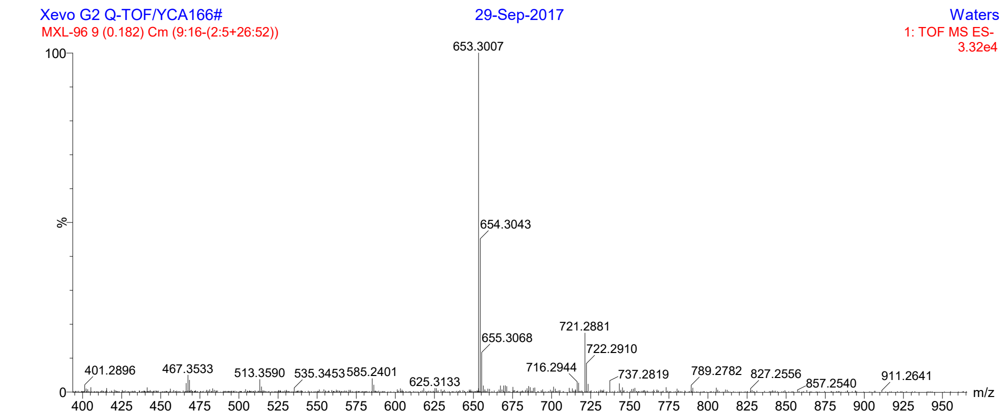


| Mass | Calc. Mass | mDa | PPM | DBE | i-FIT | Norm | Conf(%) | Formula |
|---|---|---|---|---|---|---|---|---|
| 653.3007 | 653.3015 | -0.8 | -1.2 | 23.5 | 53.5 | 0.000 | 99.99 | C42 H41 N2 O5 |

**Figure S47** HRESIMS for compound **8**.

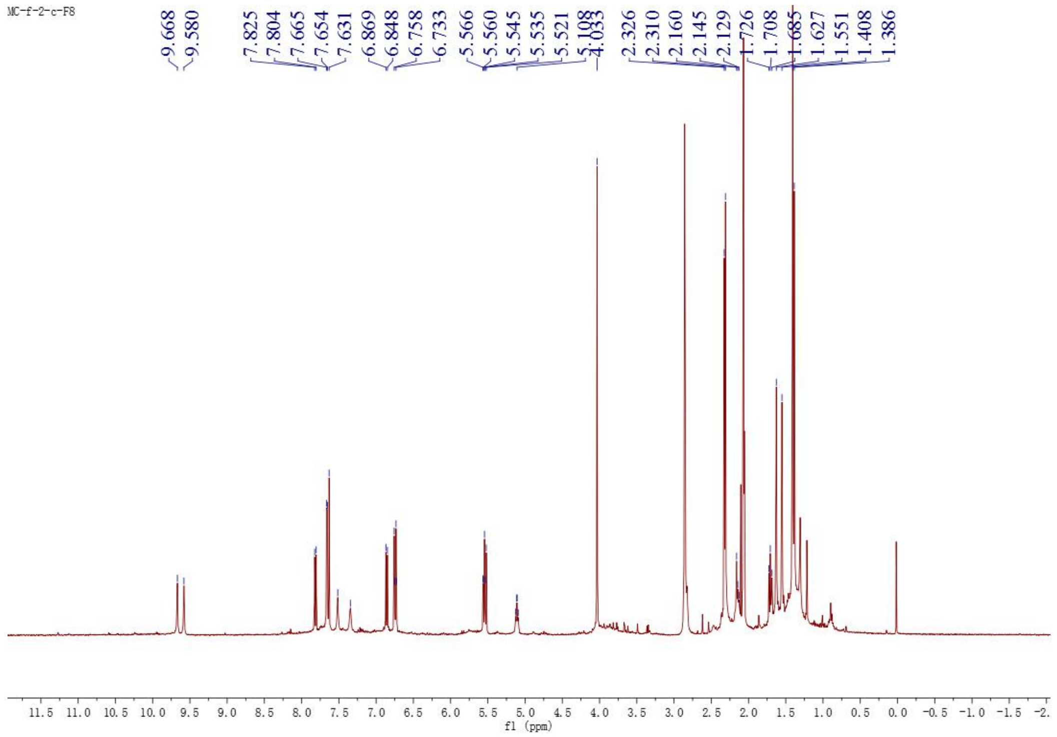


**Figure S48** $^{1}$H NMR spectrum (400 MHz, acetone-$d_6$) of compound **8**.

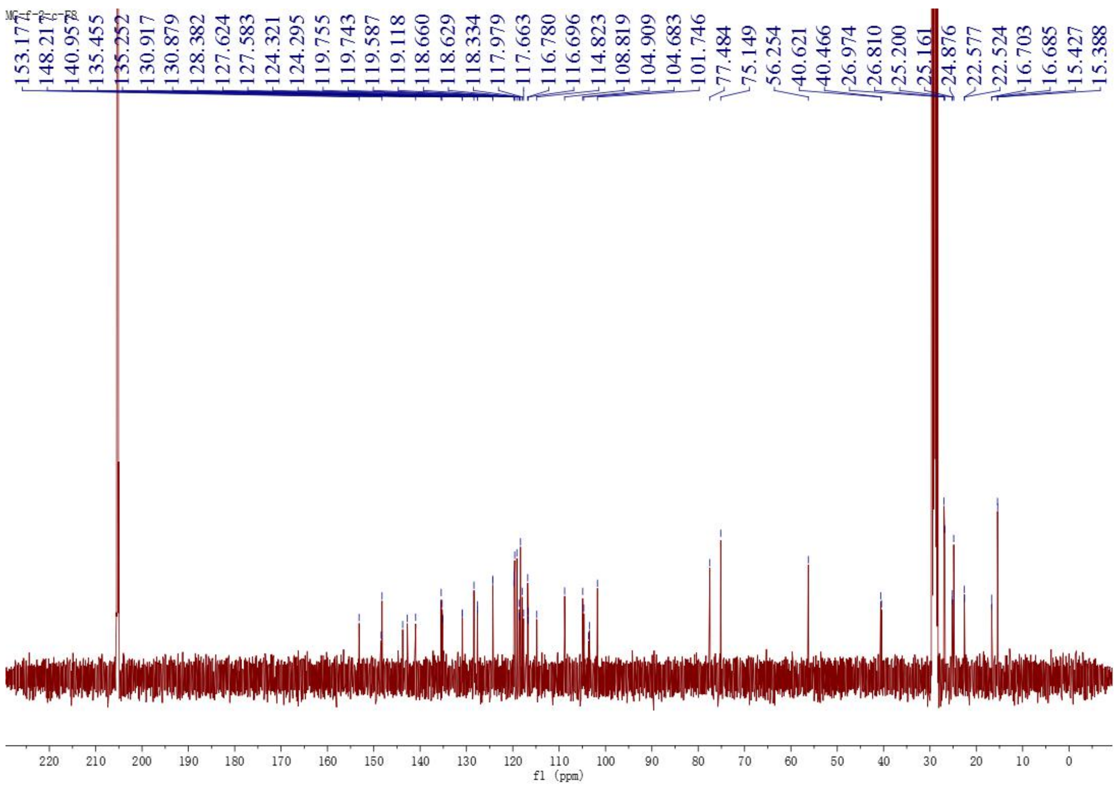


**Figure S49** $^{13}C$ NMR spectrum (100 MHz, acetone-$d_6$) of compound **8**.

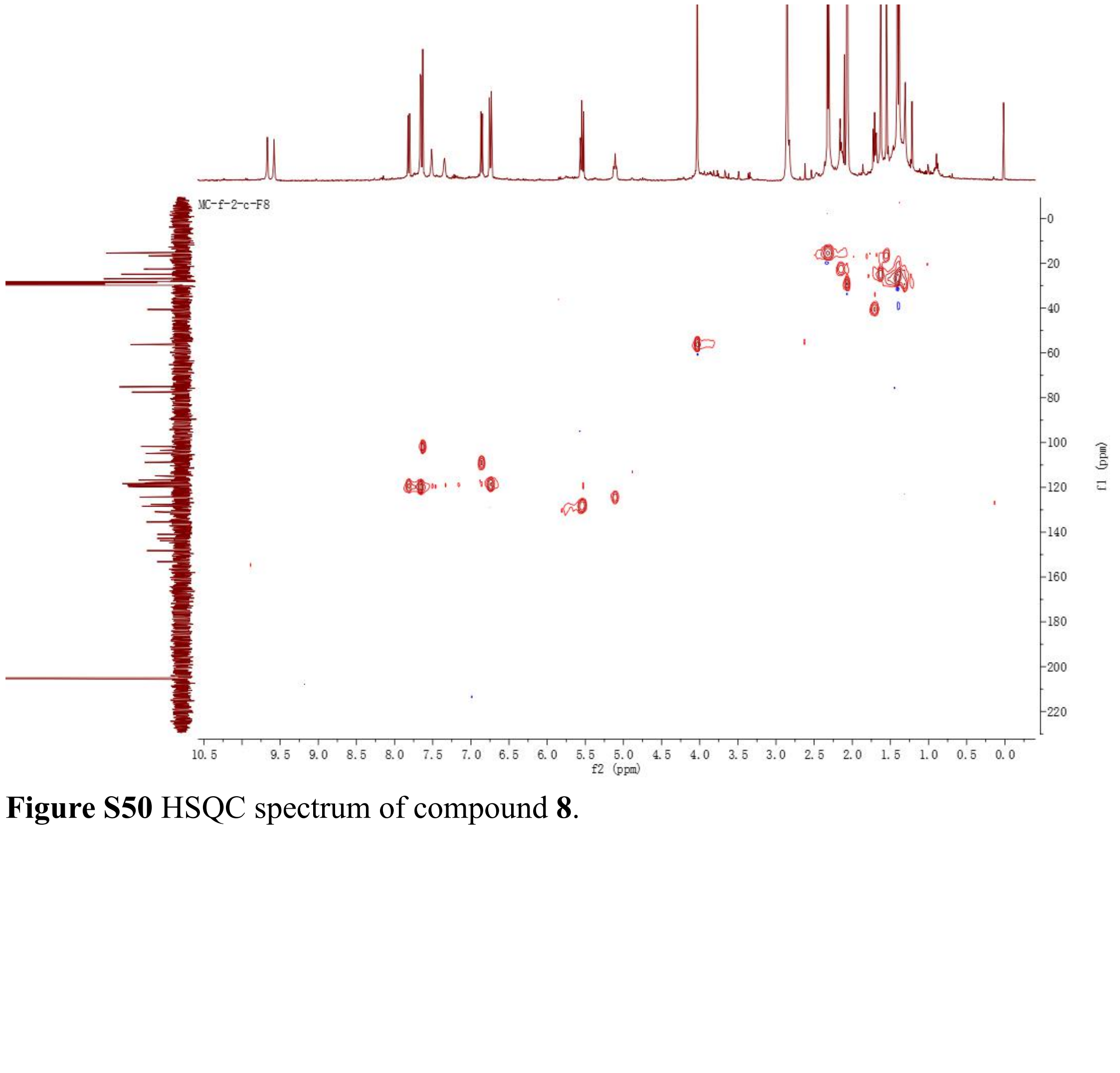


**Figure S50** HSQC spectrum of compound **8**.

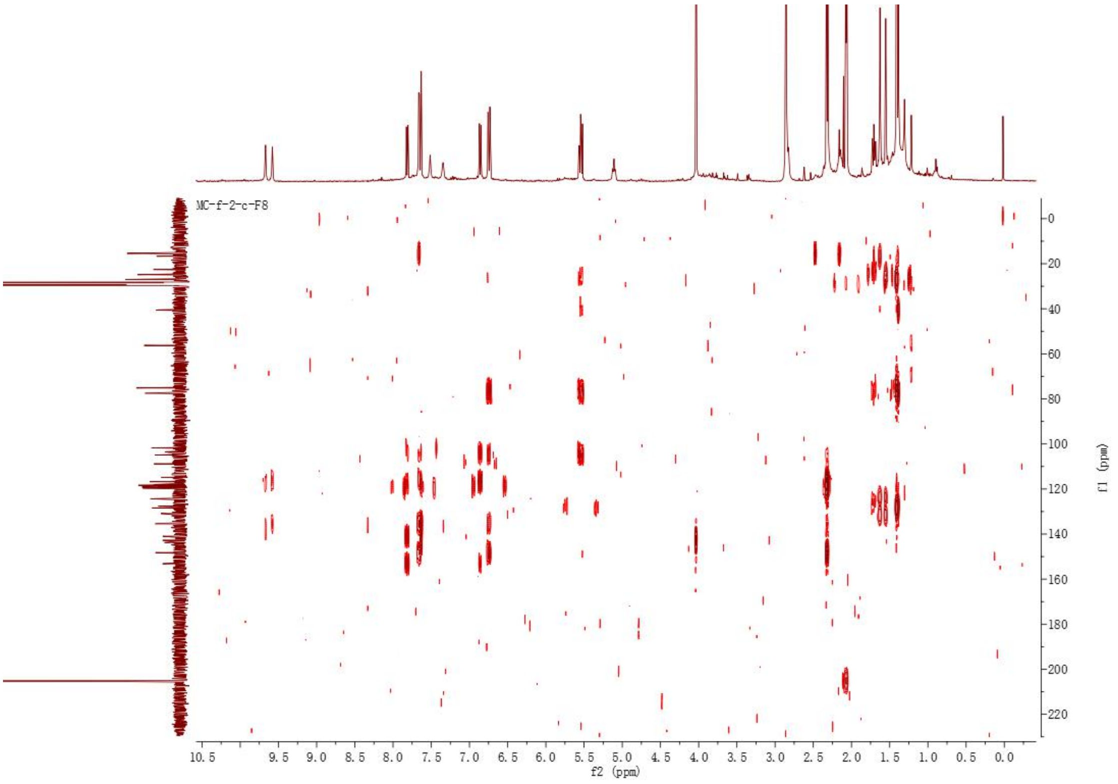


**Figure S51** HMBC spectrum of compound **8**.

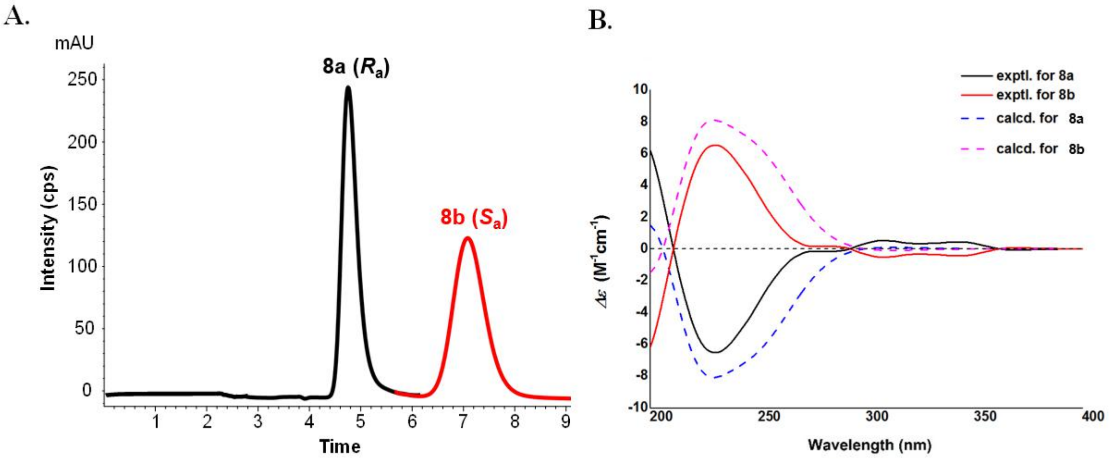


**Figure S52** The chiral HPLC separation (A) and experimental and calculated ECD data (B) of compounds **8a** and **8b**.

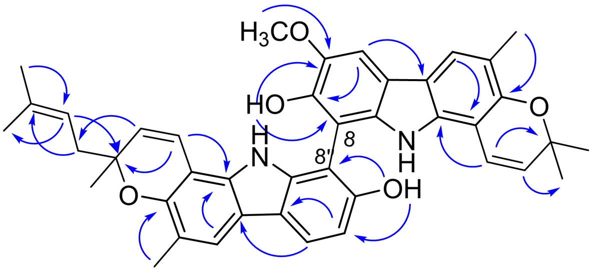


**Figure S53** Key HMBC correlations of compounds **8.**

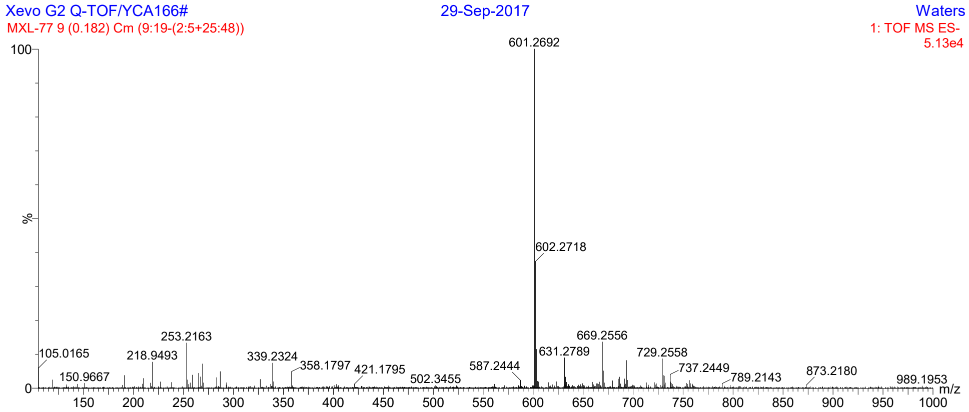


| Mass | Calc. Mass | m*Da* | PPM | *DBE* | i-FIT | Norm | Conf(%) | Formula |
|---|---|---|---|---|---|---|---|---|
| 601.2692 | 601.2702 | -1.0 | -1.7 | 21.5 | 157.7 | 0.013 | 98.66 | C38 H37 N2 O5 |

**Figure S54** HRESIMS for compound **9**.

.

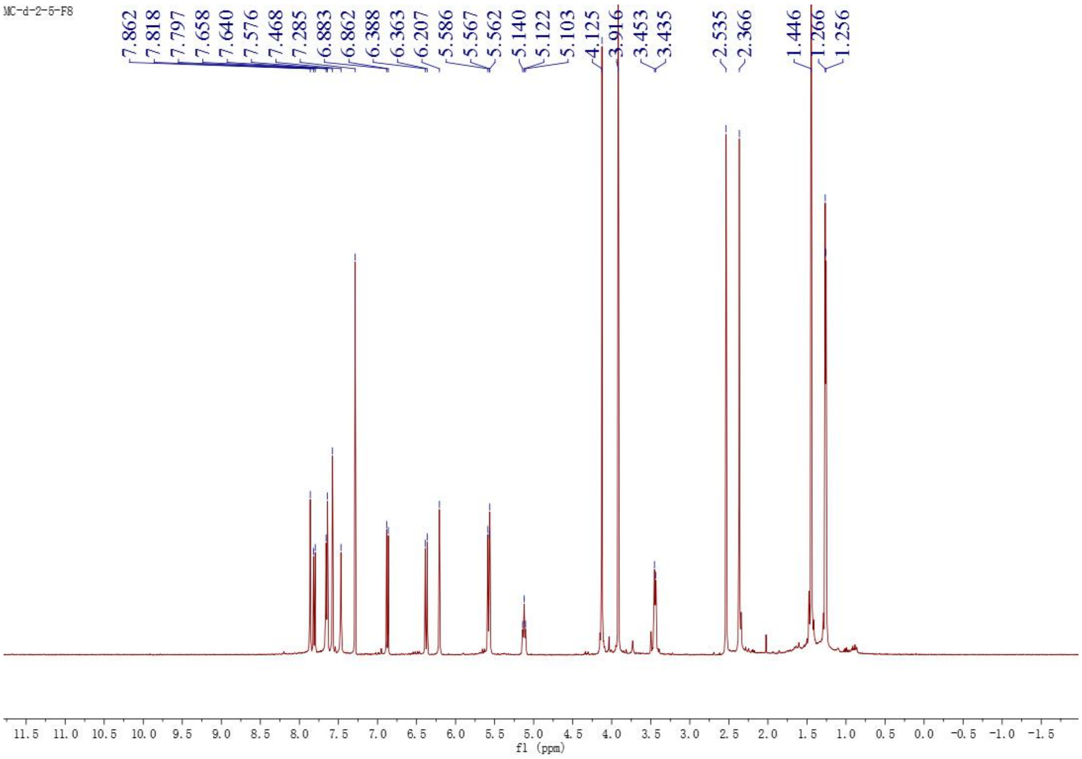


**Figure S55** $^{1}H$ NMR spectrum (400 MHz, $CDCl_3$) of compound **9**.

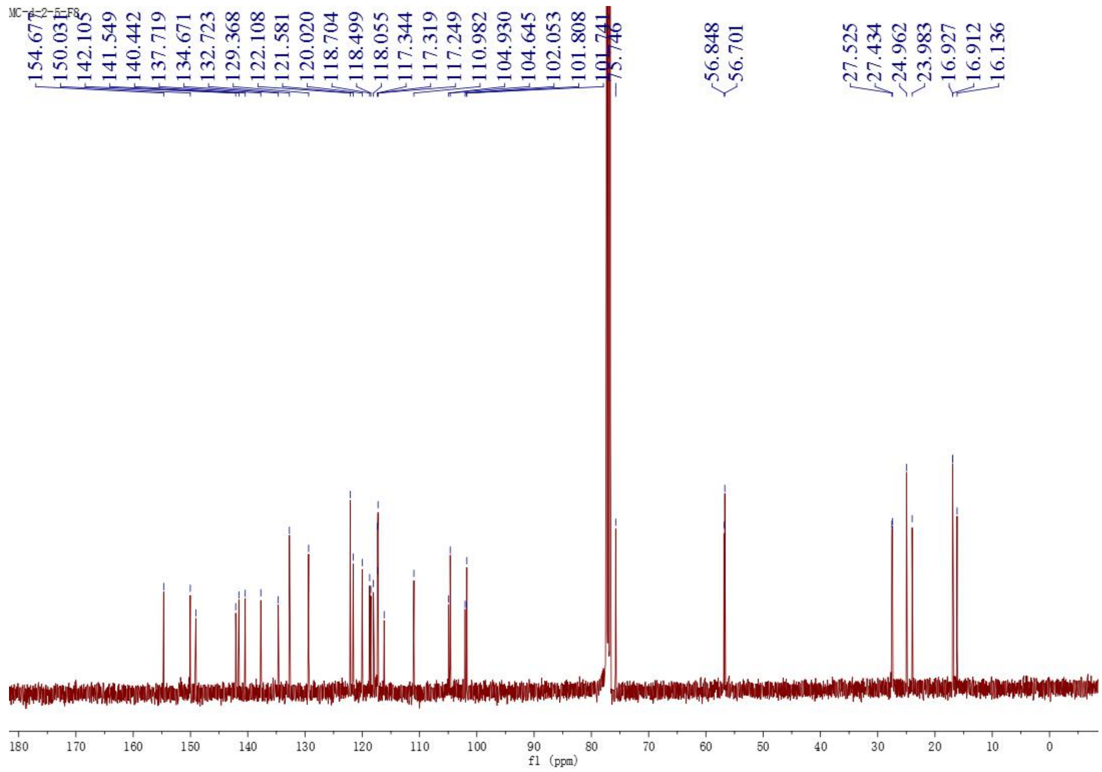


**Figure S56** $^{13}C$ NMR spectrum (100 MHz, $CDCl_3$) of compound **9**.

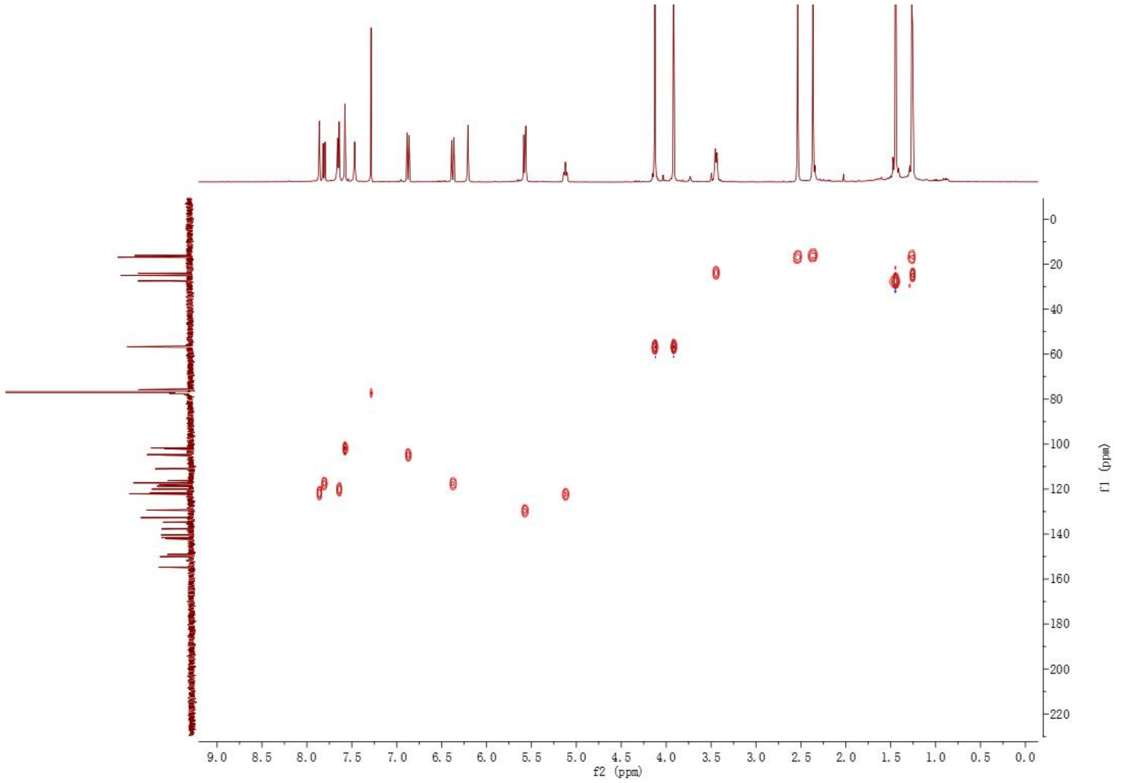


**Figure S57** HSQC spectrum of compound **9**.

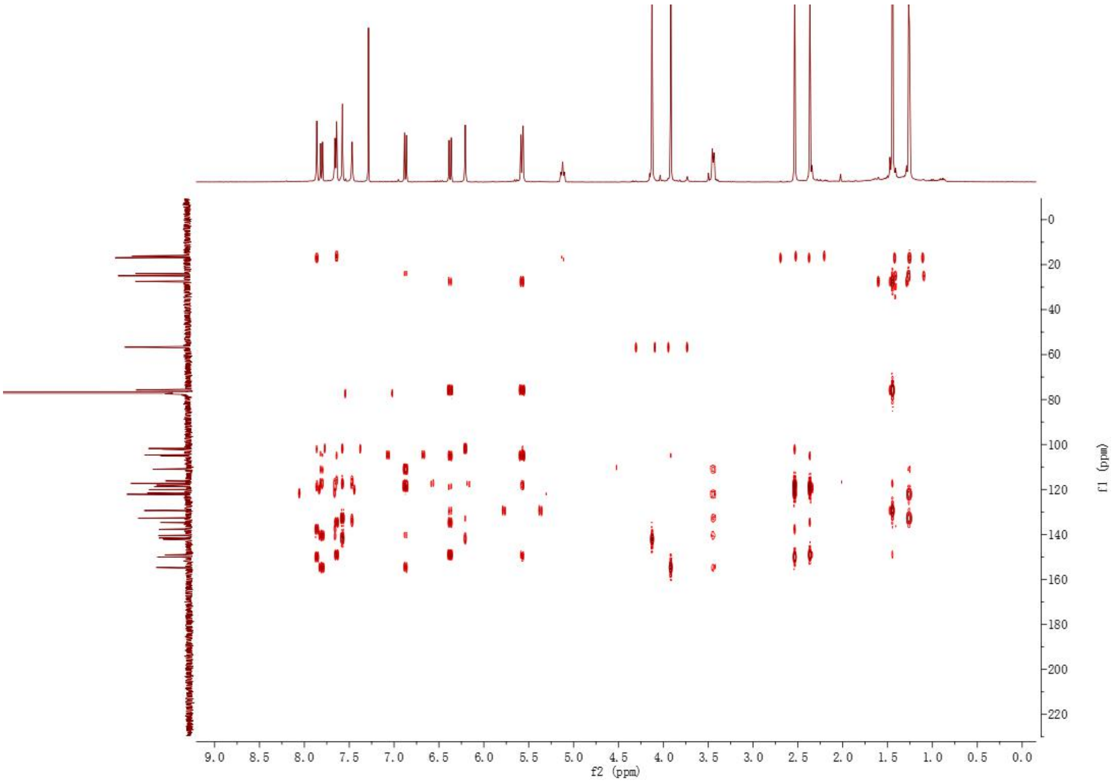


**Figure S58** HMBC spectrum of compound **9**.

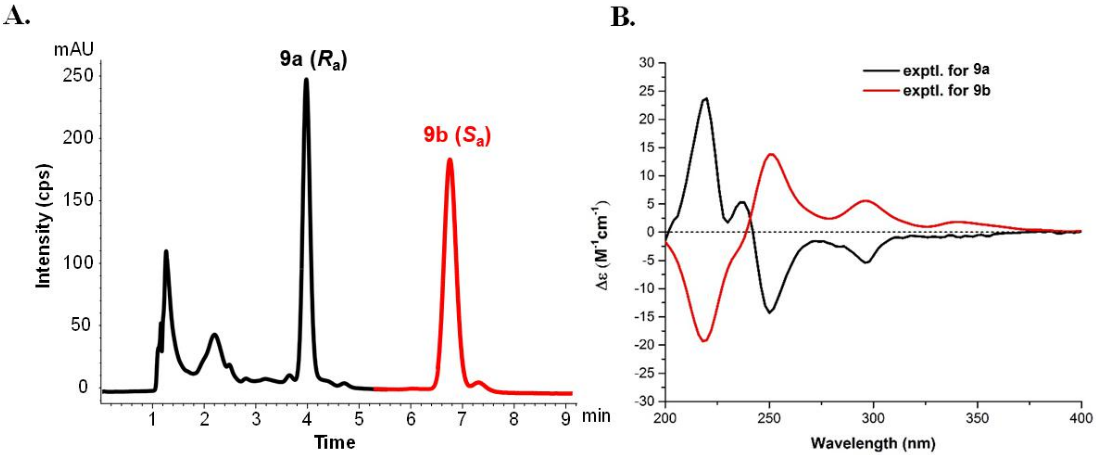


**Figure S59** The chiral HPLC separation (A) and experimental ECD data (B) of compounds **9a** and **9b**.

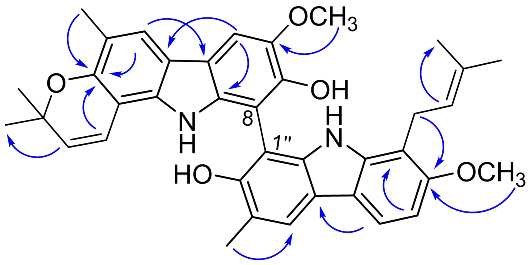


**Figure S60** Key HMBC correlations of compounds **9.**

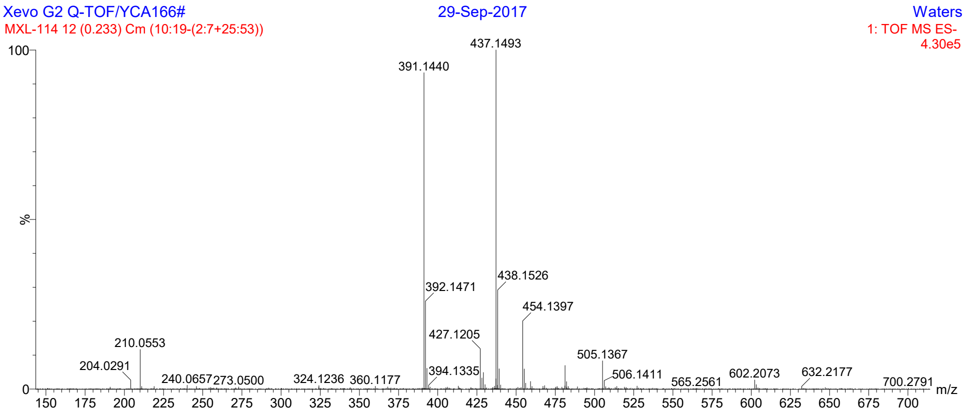


| Mass | Calc. Mass | m*D*a | PPM | *D*BE | i-FIT | Norm | Conf(%) | Formula |
|---|---|---|---|---|---|---|---|---|
| 391.1440 | 391.1447 | -0.7 | -1.8 | 18.5 | 299.0 | 0.016 | 98.41 | C26 H19 N2 O2 |

**Figure S61** HRESIMS for compound **10**.

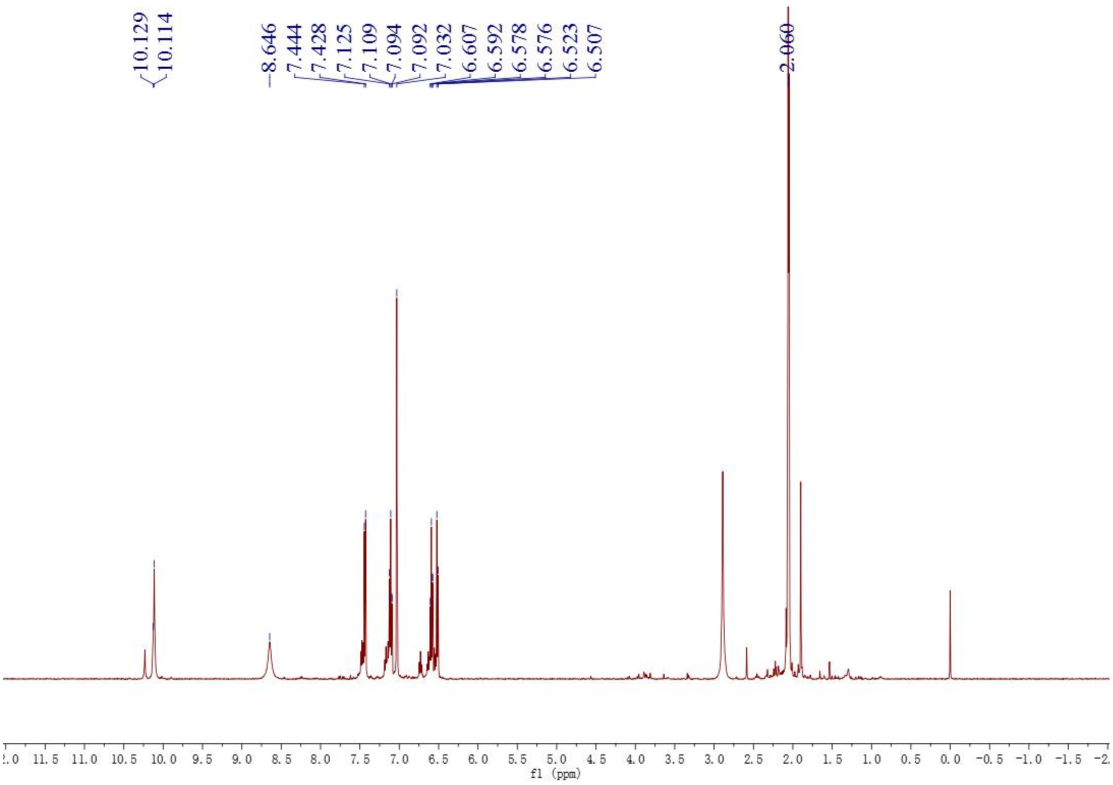


**Figure S62** $^{1}$H NMR spectrum (500 MHz, acetone-$d_6$) of compound **10**.

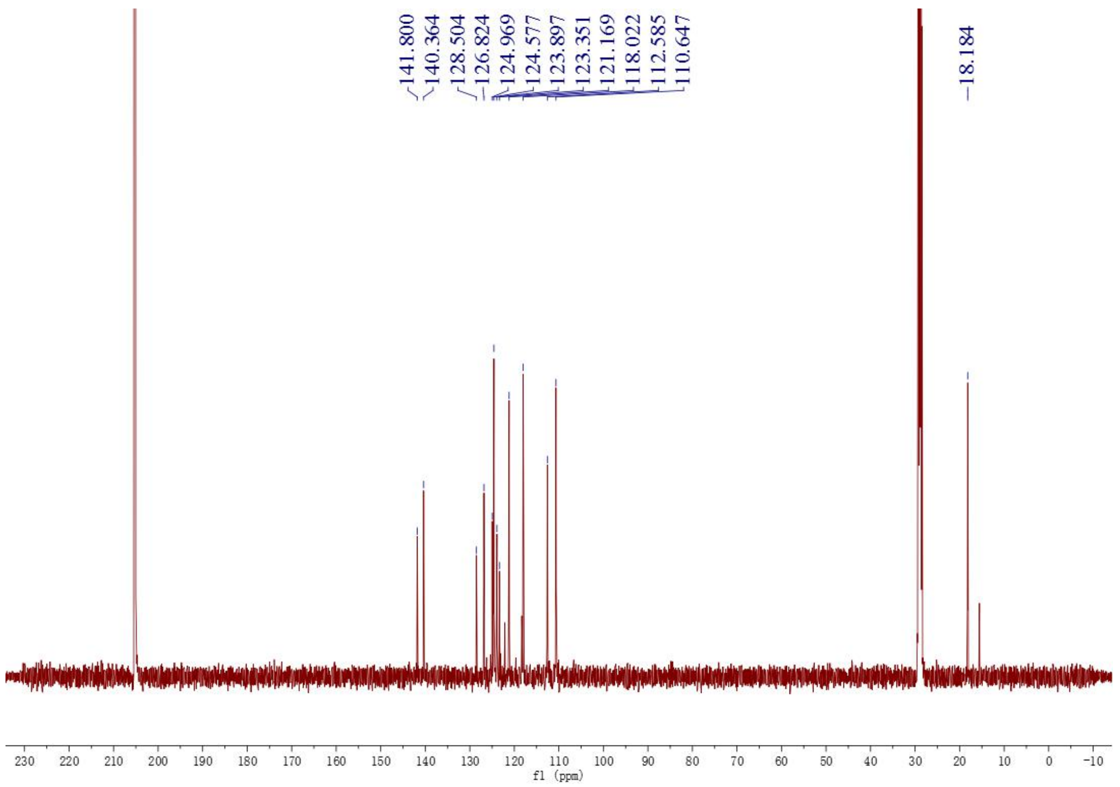


**Figure S63** $^{13}$C NMR spectrum (125 MHz, acetone-$d_6$) of compound **10**.

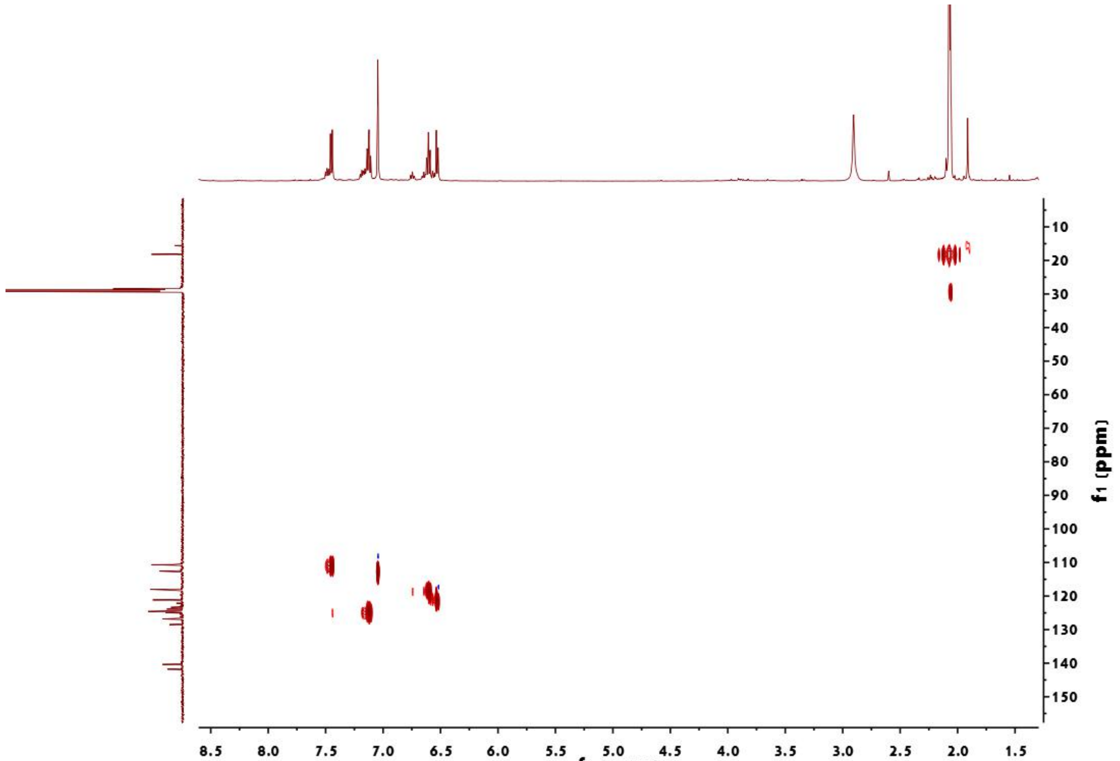


**Figure S64** HSQC spectrum of compound **10**.

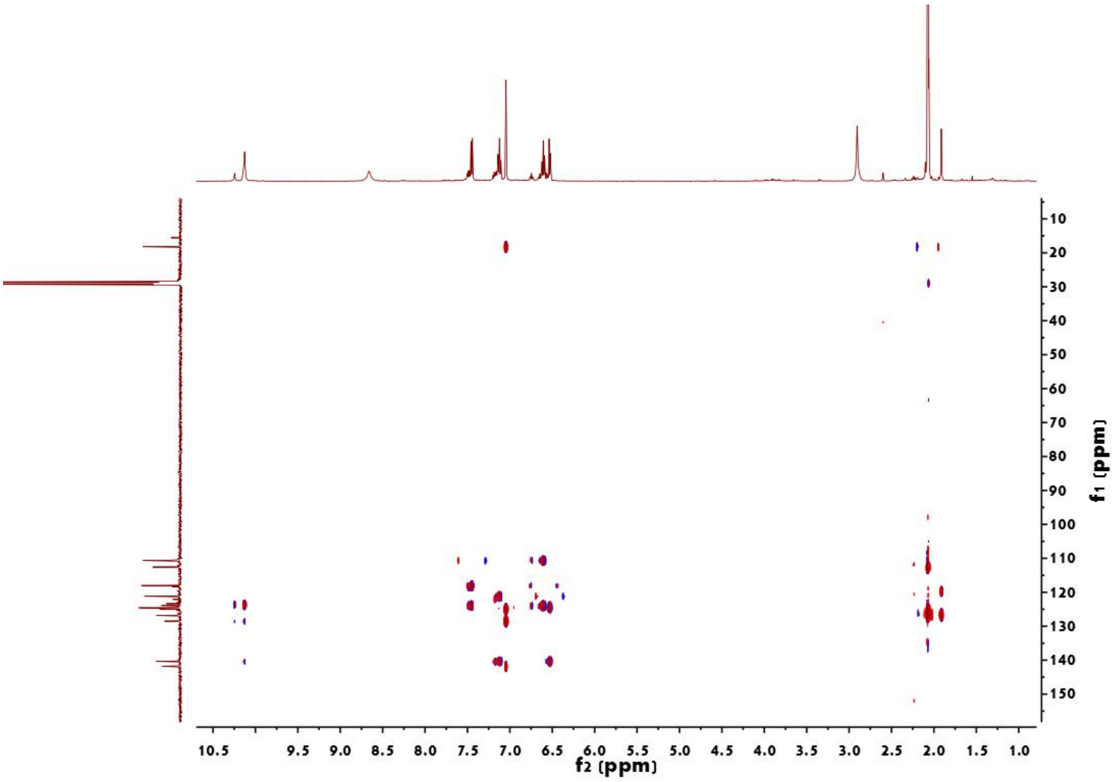


**Figure S65** HMBC spectrum of compound **10**.

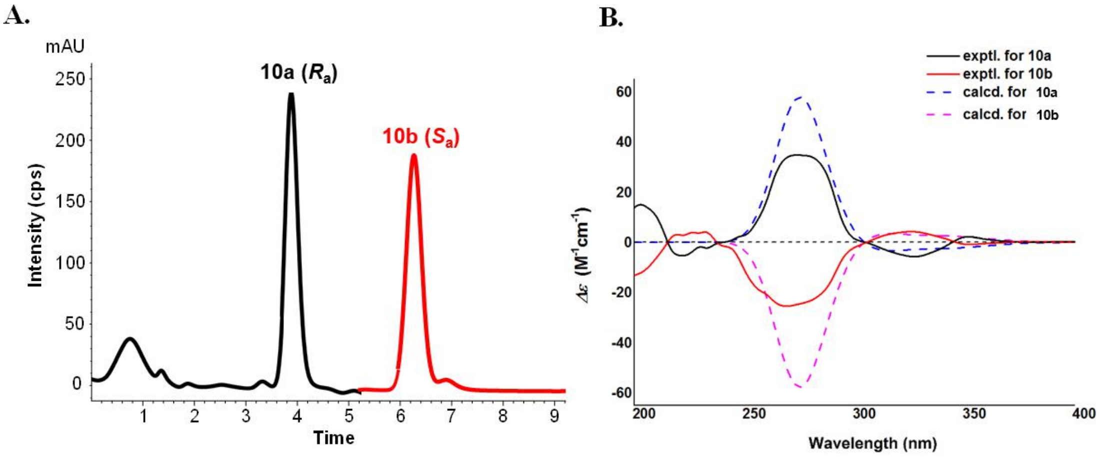


**Figure S66** The chiral HPLC separation (A) and experimental and calculated ECD data (B) of compounds **10a** and **10b**.

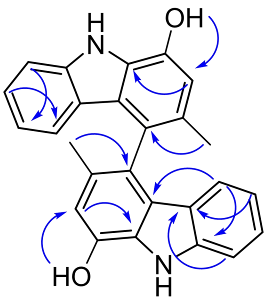


**Figure S67** Key HMBC correlations of compounds **10.**

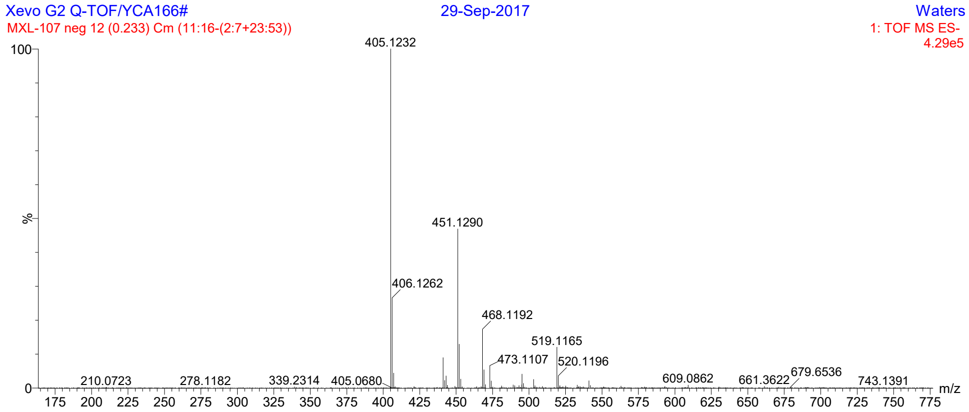


| Mass | Calc. Mass | mDa | PPM | DBE | i-FIT | Norm | Conf(%) | Formula |
|---|---|---|---|---|---|---|---|---|
| 405.1232 | 405.1239 | -0.7 | -1.7 | 19.5 | 302.1 | 0.000 | 99.99 | C26 H17 N2 O3 |

**Figure S68** HRESIMS for compound **11**.

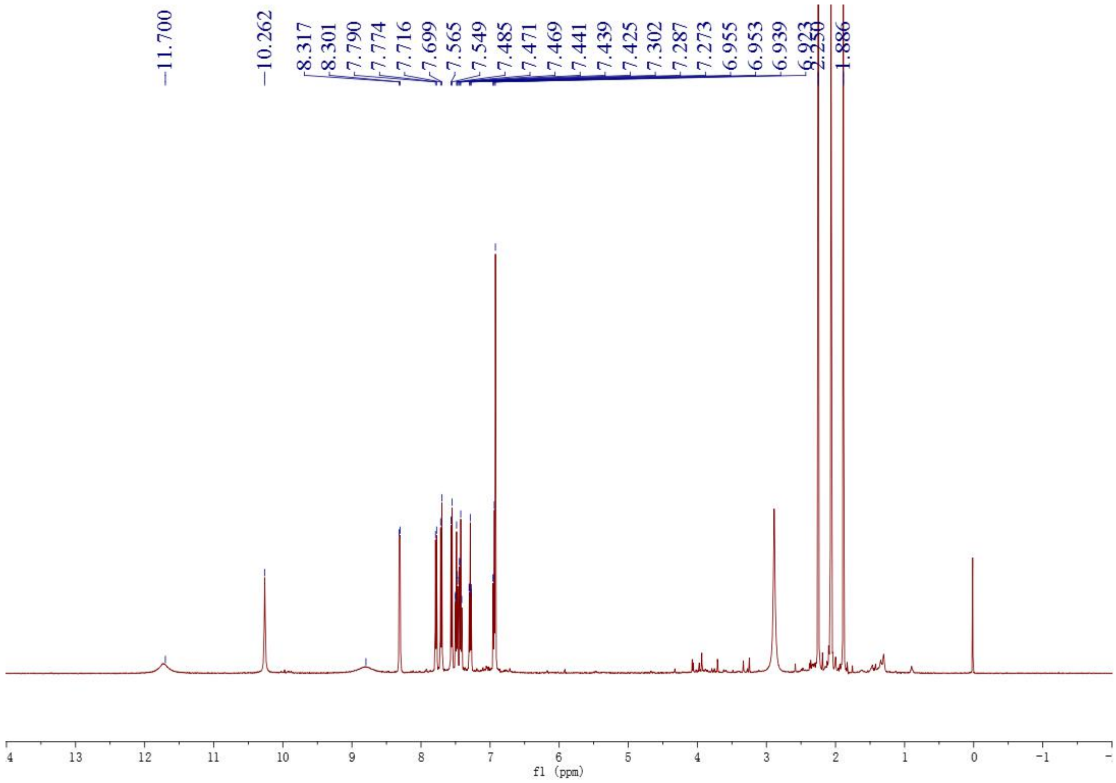


**Figure S69** $^{1}$H NMR spectrum (500 MHz, acetone-$d_6$) of compound **11**.

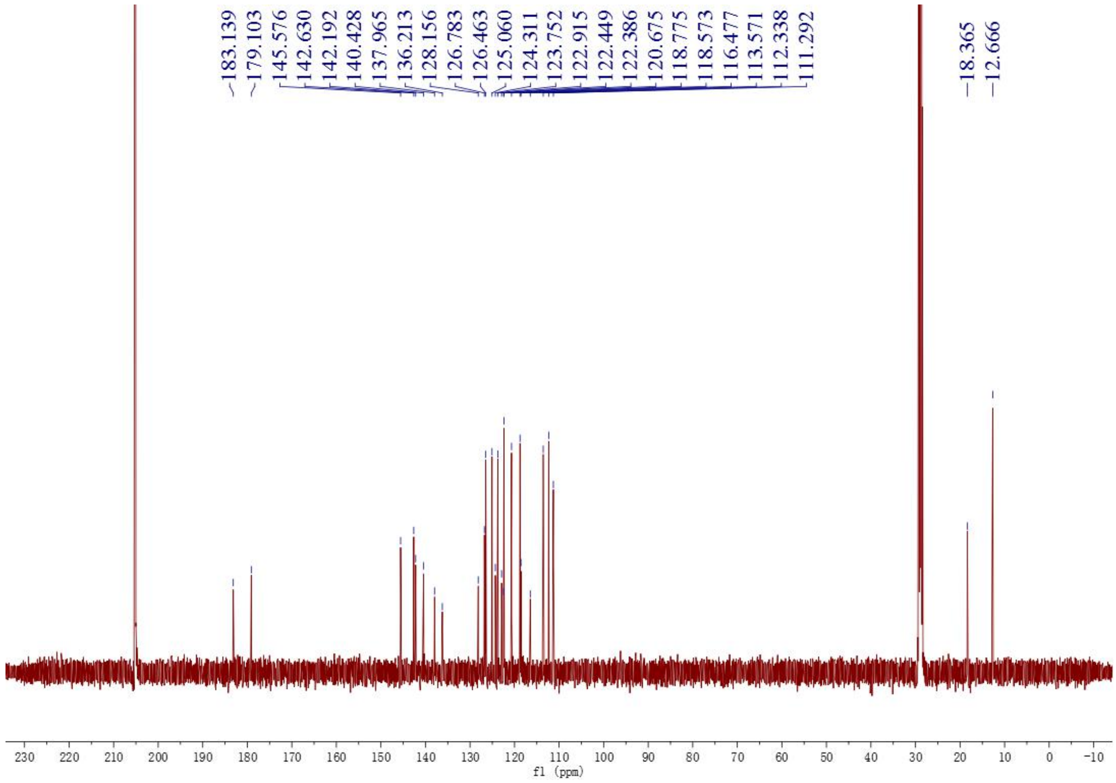


**Figure S70** $^{13}C$ NMR spectrum (125 MHz, acetone-$d_6$) of compound **11**.

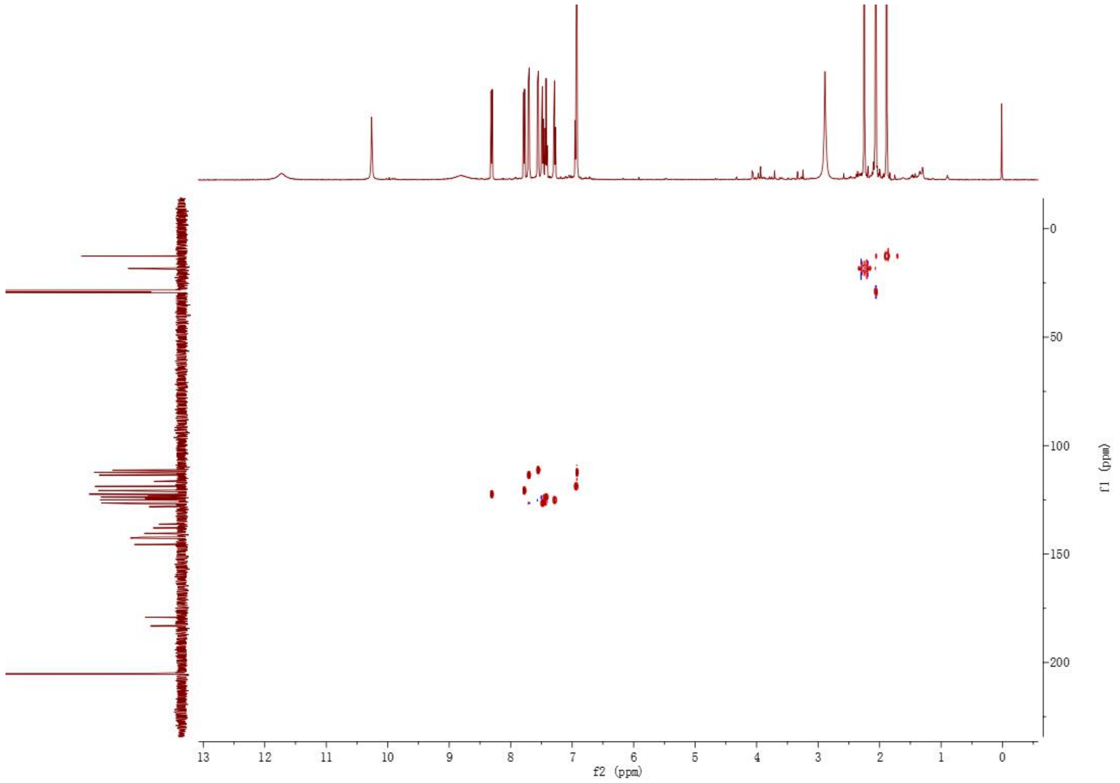


**Figure S71** HSQC spectrum of compound **11**.

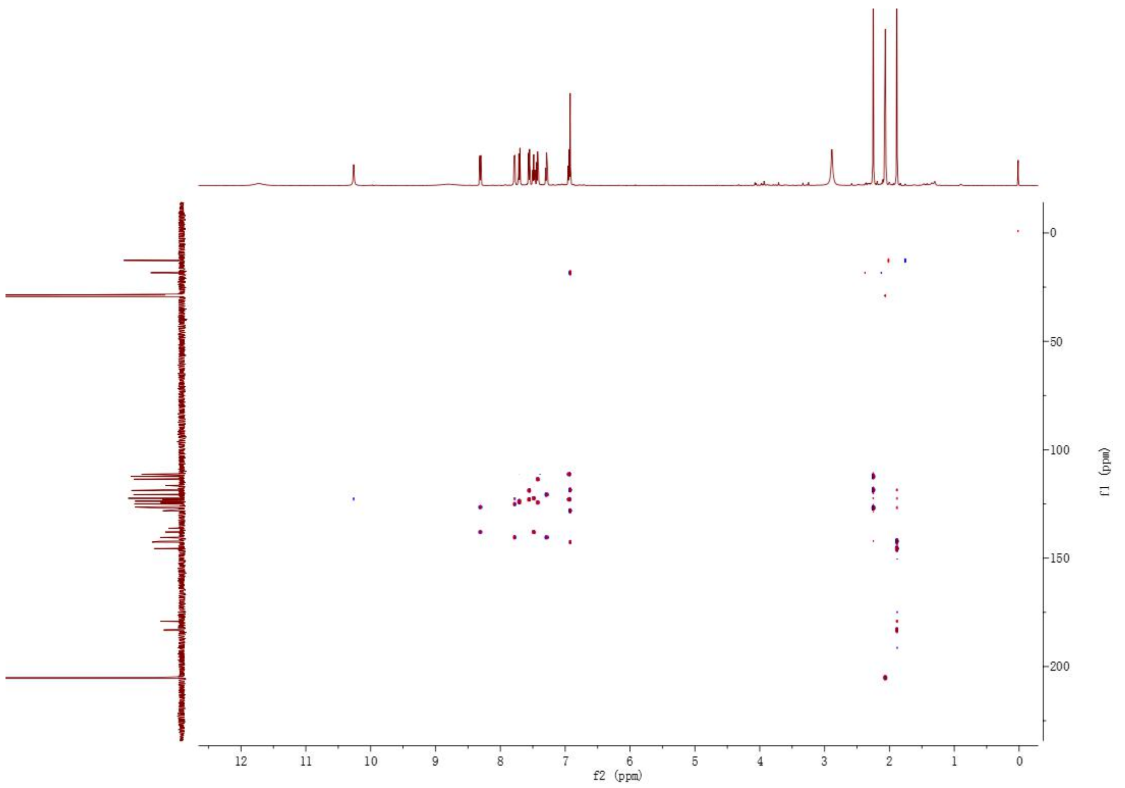


**Figure S72** HMBC spectrum of compound **11**.

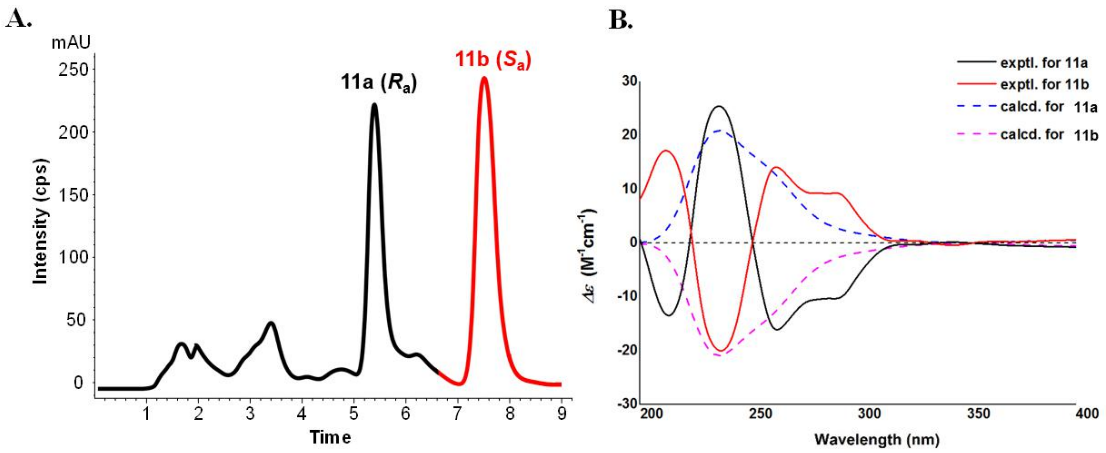


**Figure S73** The chiral HPLC separation (A) and experimental and calculated ECD data (B) of compounds **11a** and **11b**.

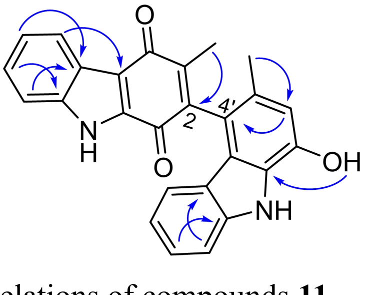


**Figure S74** Key HMBC correlations of compounds **11.**

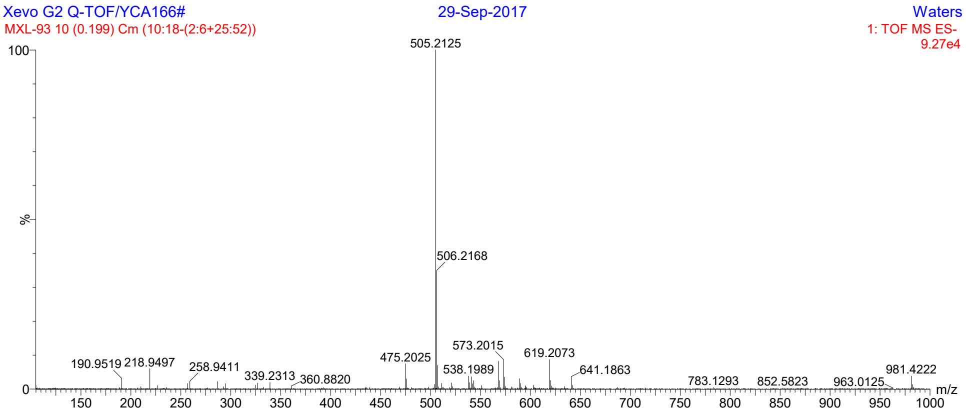


| Mass | Calc. Mass | mDa | PPM | DBE | i-FIT | Norm | Conf(%) | Formula |
|---|---|---|---|---|---|---|---|---|
| 505.2125 | 505.2127 | -0.2 | -0.4 | 19.5 | 157.3 | 0.000 | 100.00 | C32 H29 N2 O4 |

**Figure S75** HRESIMS for compound **12**.

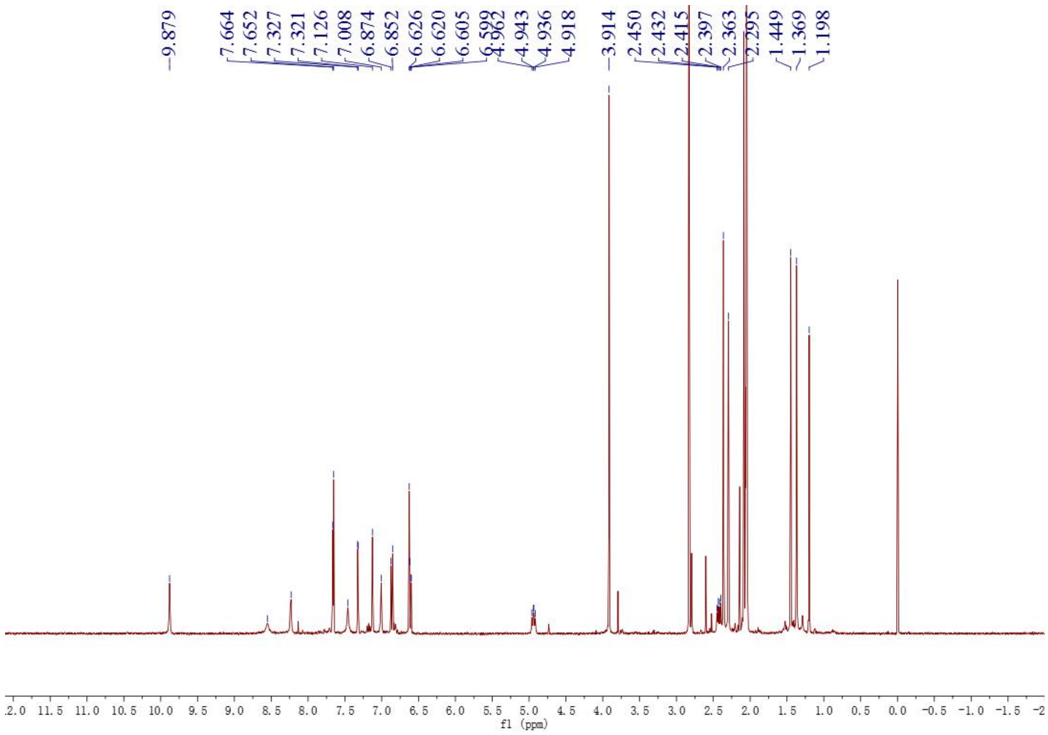


**Figure S76** $^{1}H$ NMR spectrum (400 MHz, acetone-$d_6$) of compound **12**.

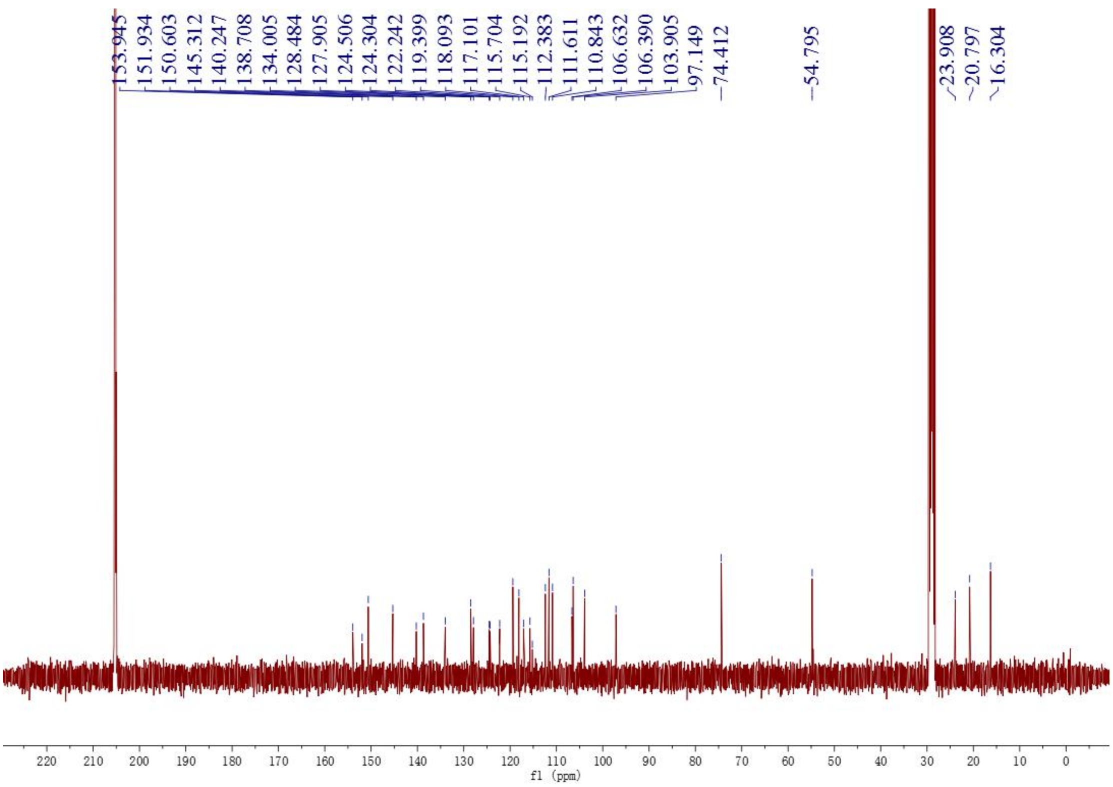


**Figure S77** $^{13}$C NMR spectrum (100 MHz, acetone-$d_6$) of compound **12**.

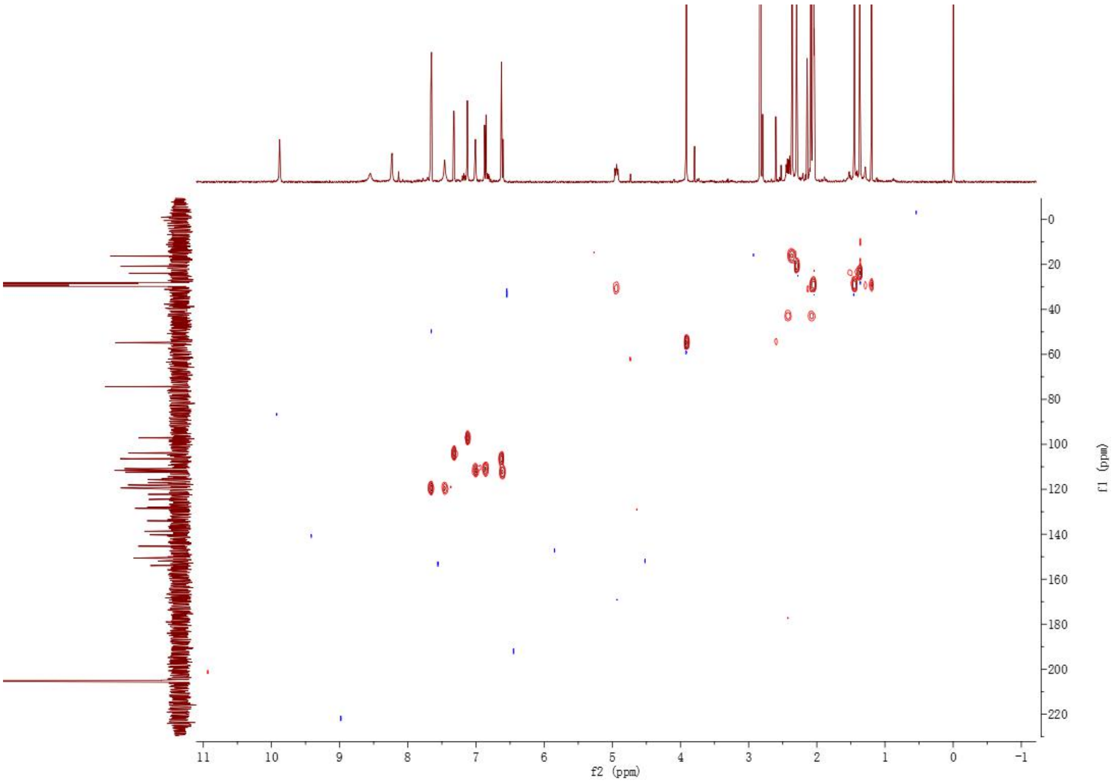


**Figure S78** HSQC spectrum of compound **12**.

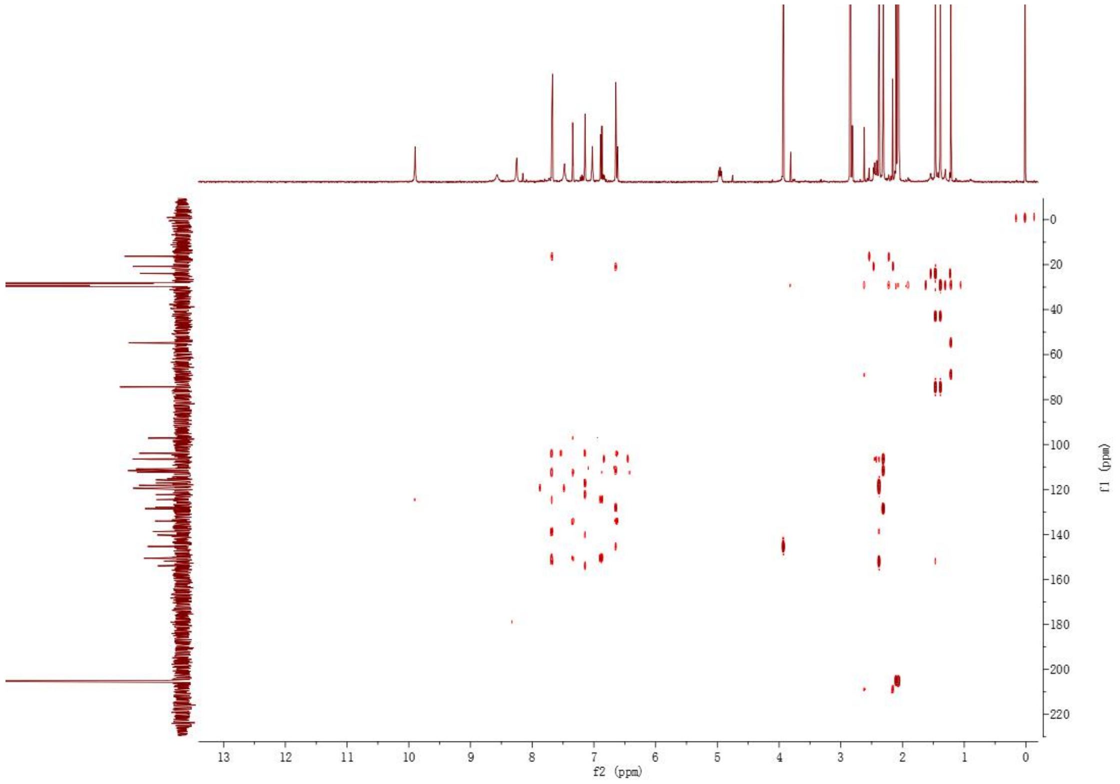


**Figure S79** HMBC spectrum of compound **12**.

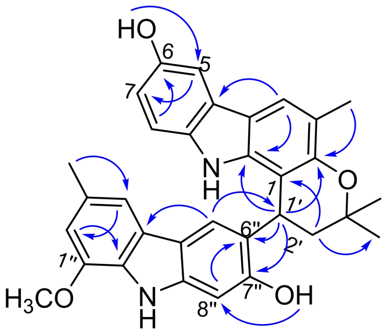


**Figure S80** Key HMBC correlations of compounds **12.**

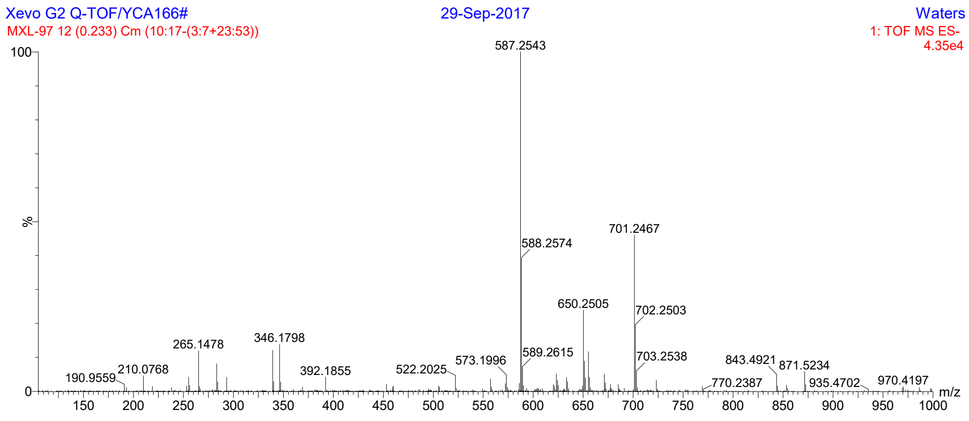


| Mass | Calc. Mass | mDa | PPM | DBE | i-FIT | Norm | Conf(%) | Formula |
|---|---|---|---|---|---|---|---|---|
| 587.2543 | 587.2546 | -0.3 | -0.5 | 21.5 | 54.5 | 0.009 | 99.09 | C37 H35 N2 O5 |

**Figure S81** HRESIMS for compound **13**.

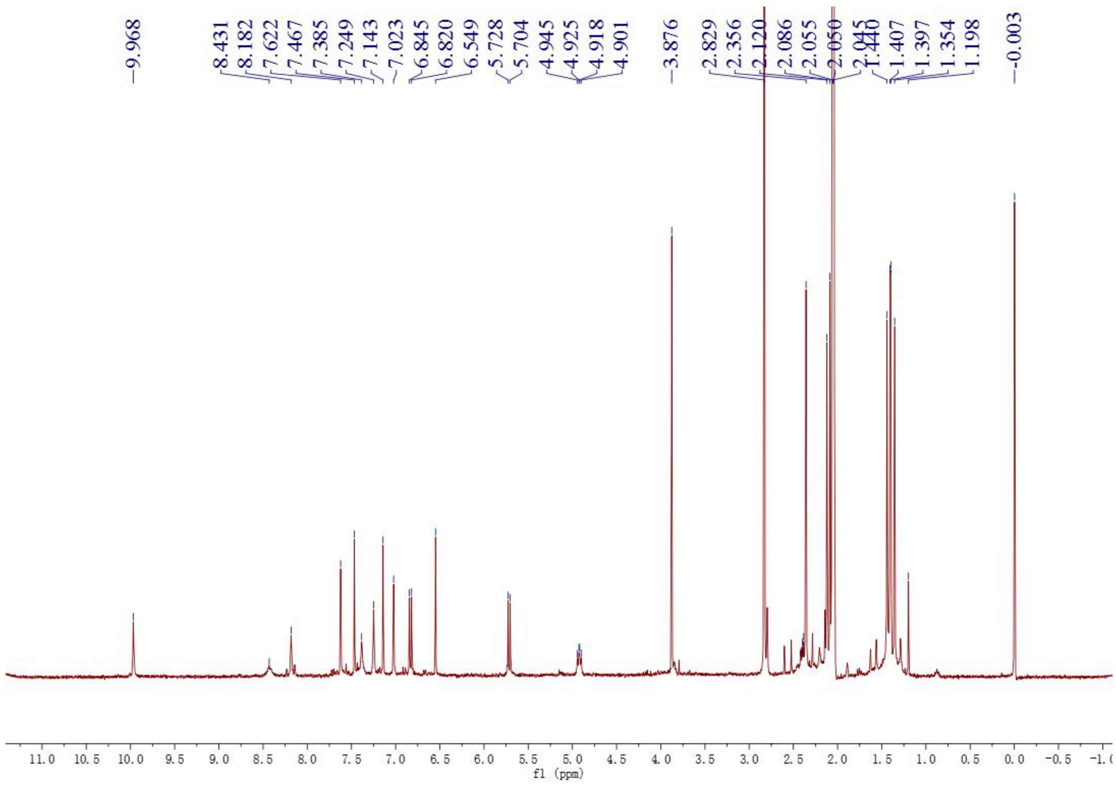


**Figure S82** $^{1}$H NMR spectrum (400 MHz, acetone-$d_6$) of compound **13**.

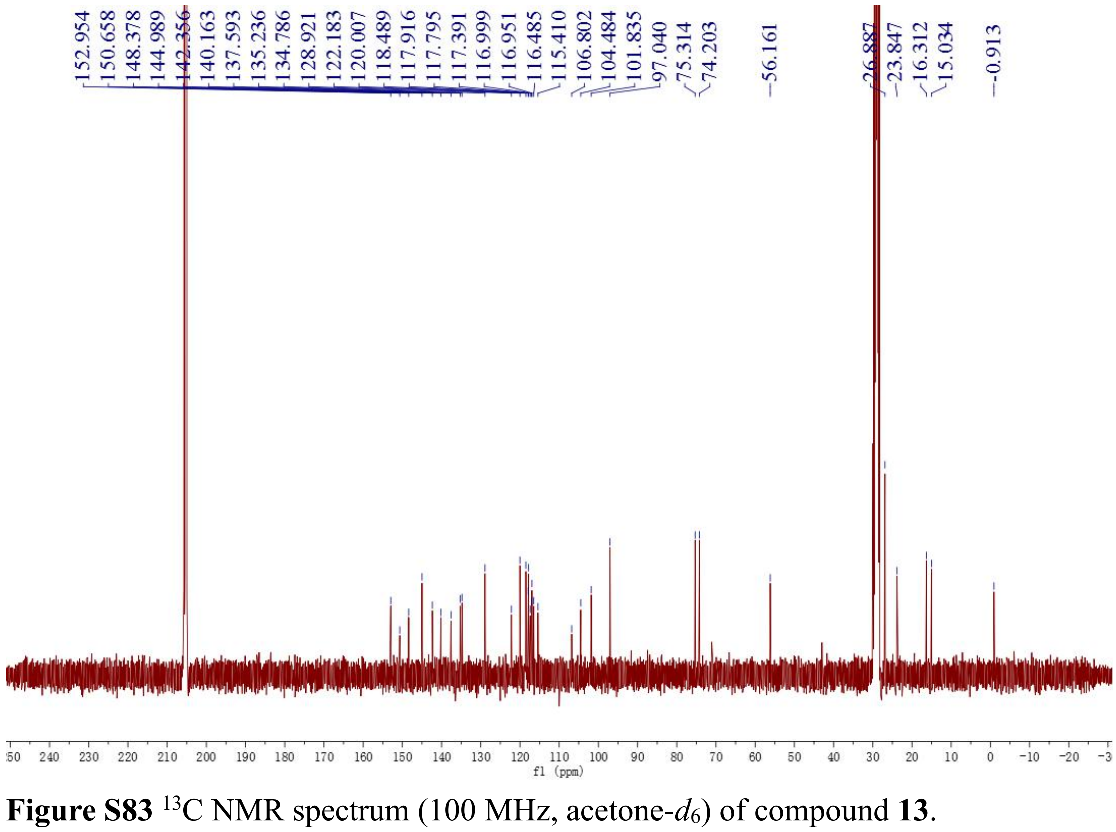


**Figure S83** $^{13}$C NMR spectrum (100 MHz, acetone-$d_6$) of compound **13**.

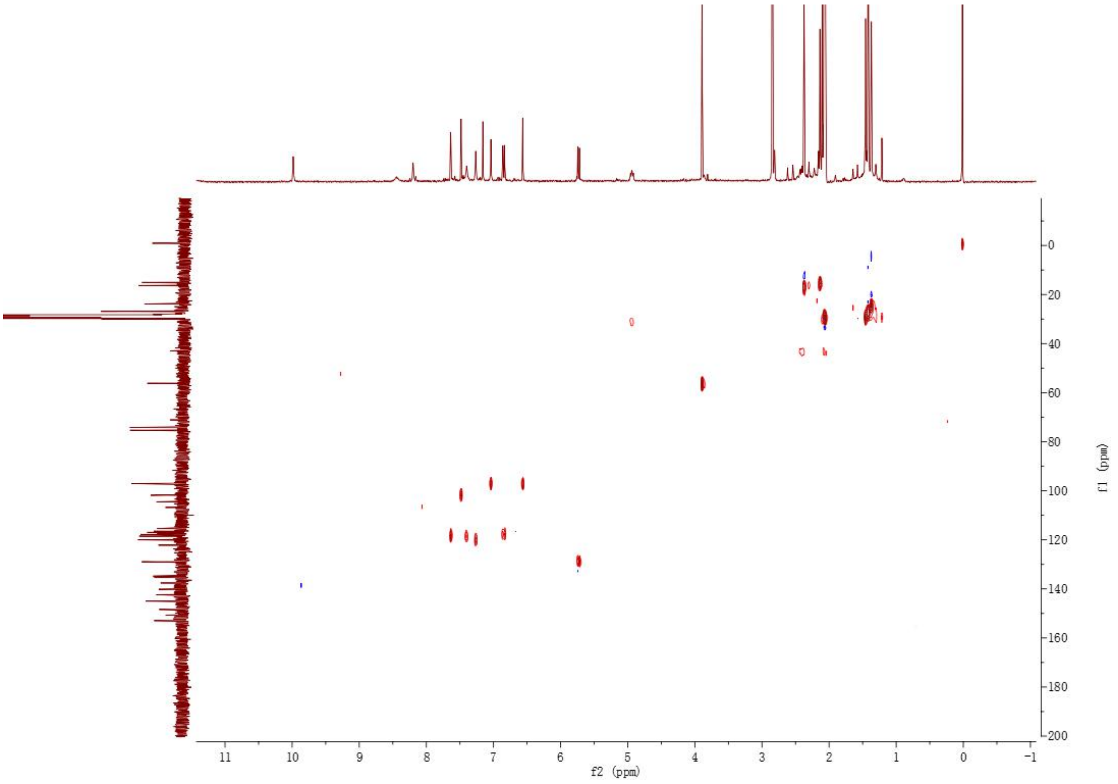


**Figure S84** HSQC spectrum of compound **13**.

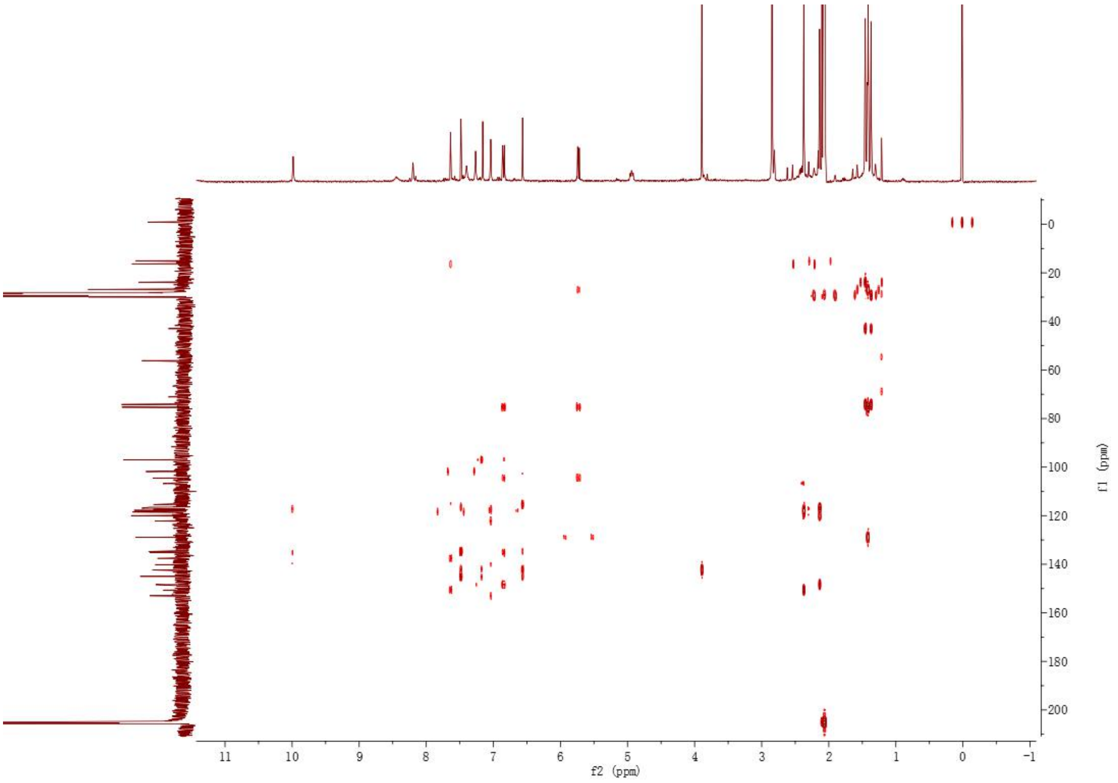


**Figure S85** HMBC spectrum of compound **13**.

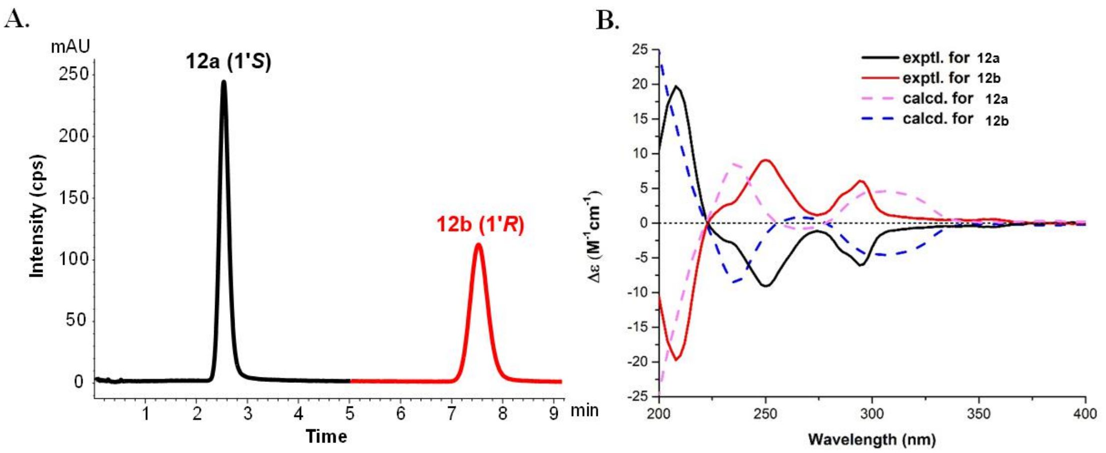


**Figure S86** The chiral HPLC separation (A) and experimental and calculated ECD data (B) of compounds **13a** and **13b**.

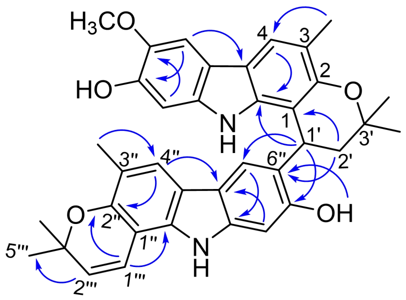

**Figure S87** Key HMBC correlations of compounds **13.**

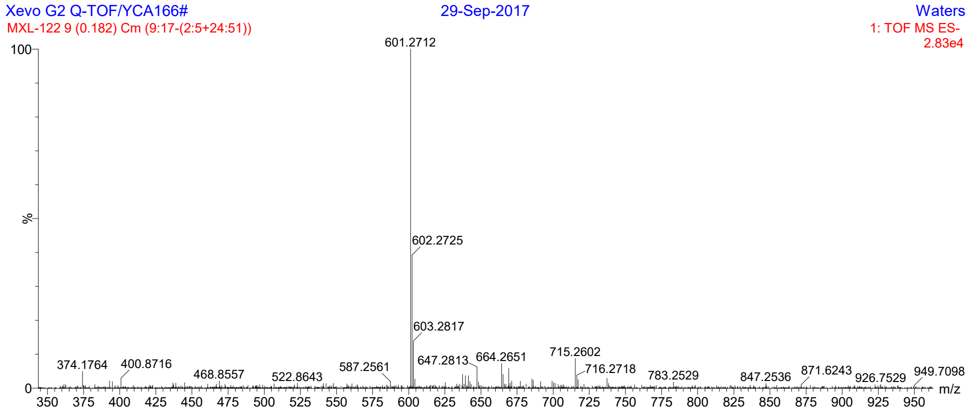


| Mass | Calc. Mass | mDa | PPM | DBE | i-FIT | Norm | Conf(%) | Formula |
|---|---|---|---|---|---|---|---|---|
| 601.2712 | 601.2702 | 1.0 | 1.7 | 21.5 | 136.4 | 0.651 | 52.17 | C38 H37 N2 O5 |

**Figure S88** HRESIMS for compound **14**.

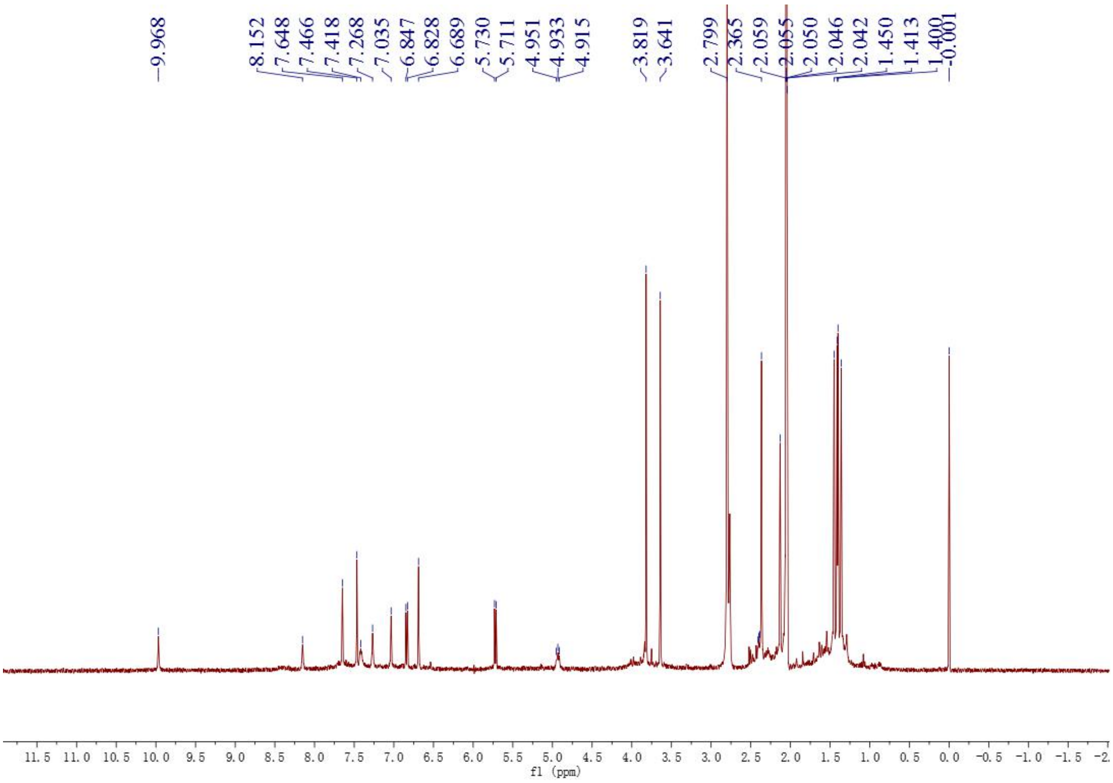


**Figure S89** $^{1}$H NMR spectrum (500 MHz, acetone-$d_6$) of compound **14**.

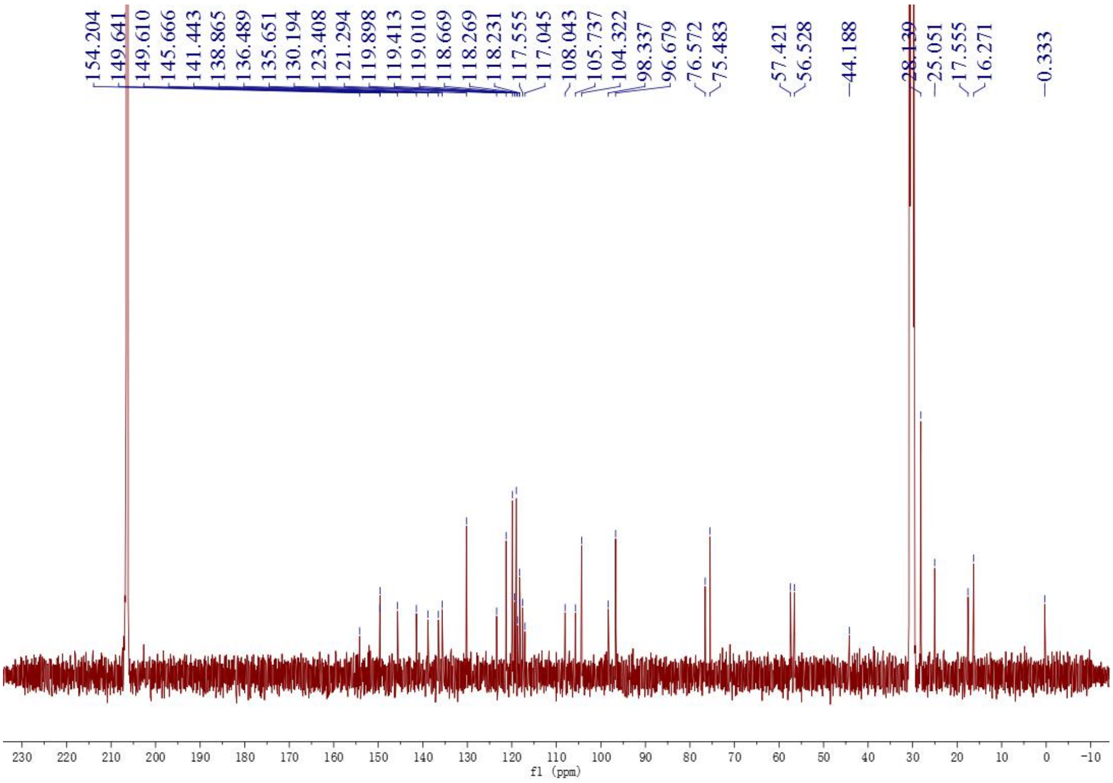


**Figure S90** $^{13}$C NMR spectrum (125 MHz, acetone-$d_6$) of compound **14**.

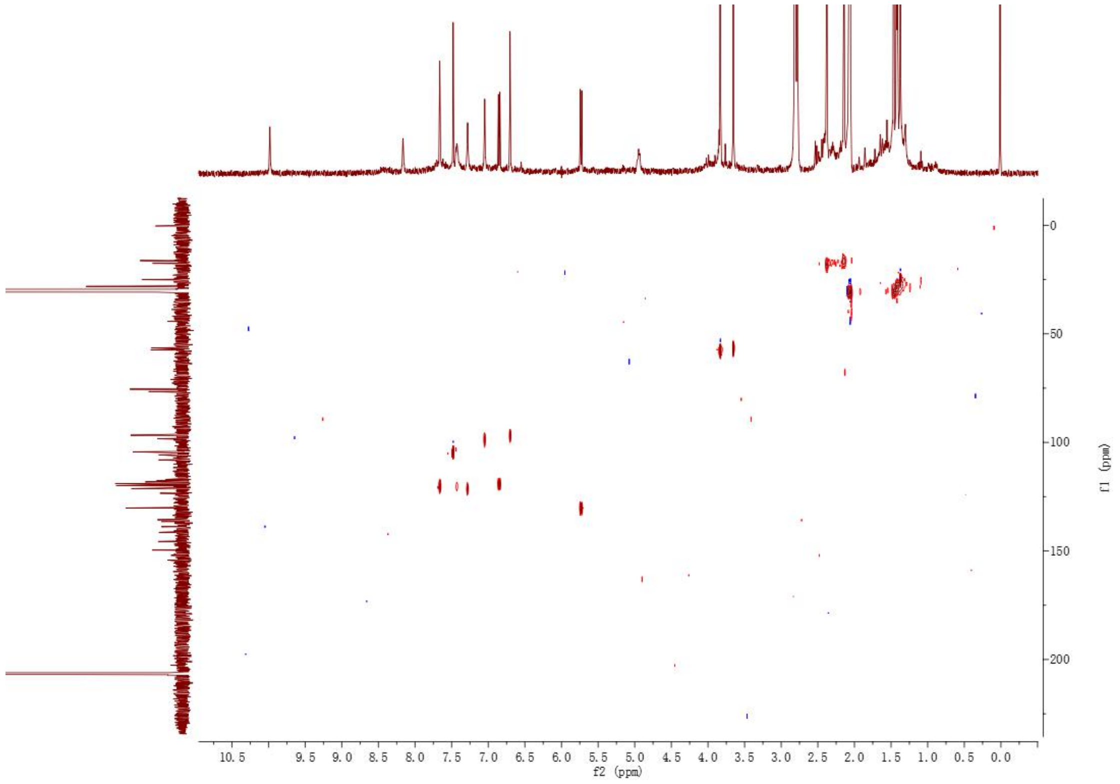


**Figure S91** HSQC spectrum of compound **14**.

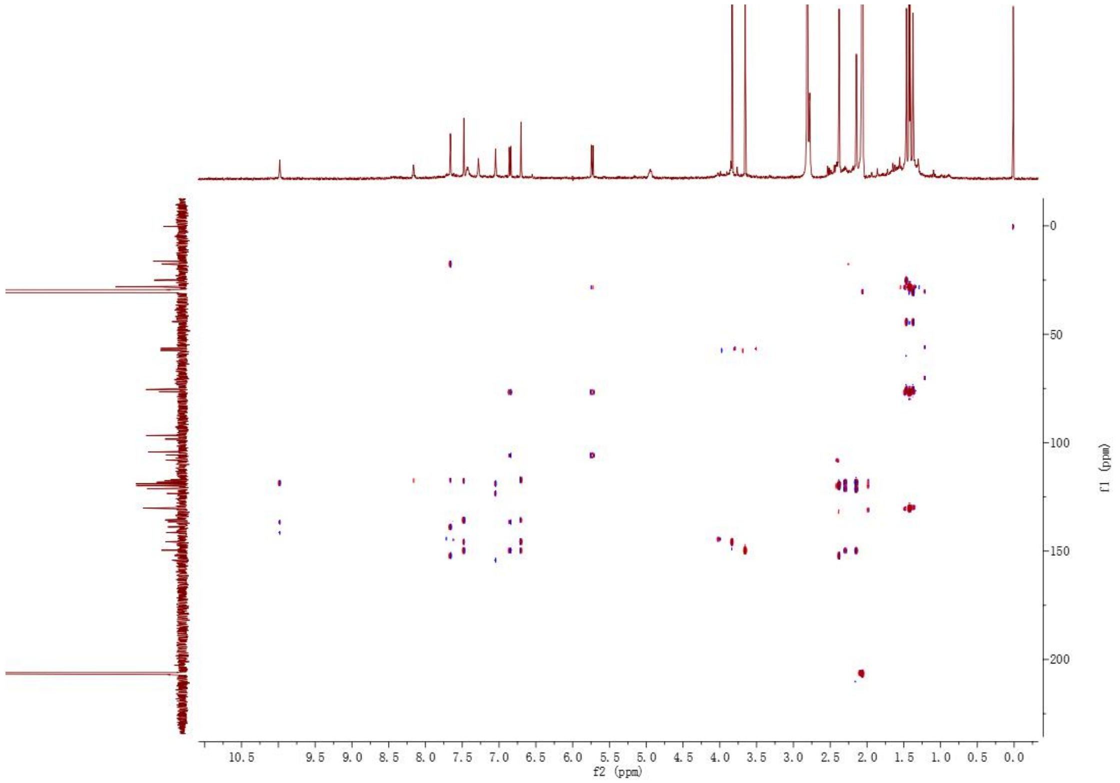


**Figure S92** HMBC spectrum of compound **14**.

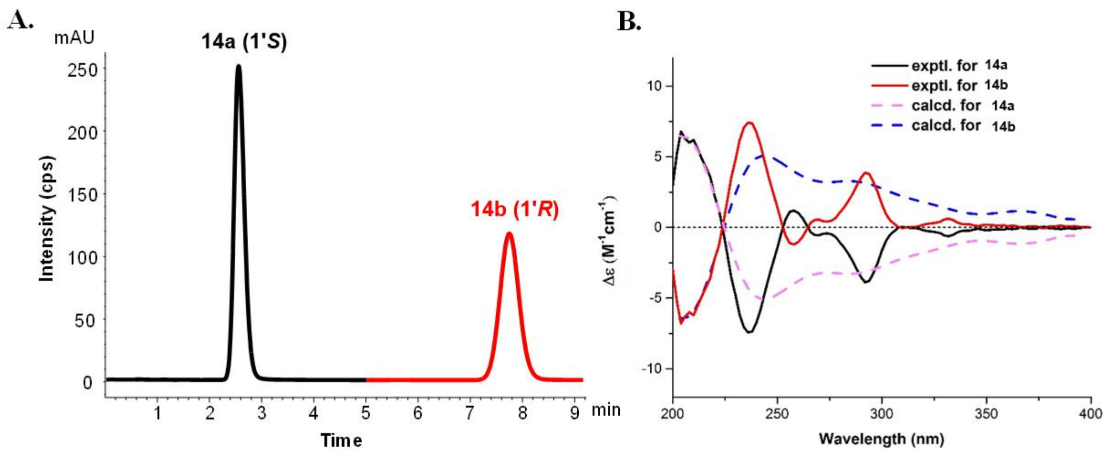


**Figure S93** The chiral HPLC separation (A) and experimental and calculated ECD data (B) of compounds **14a** and **14b**.

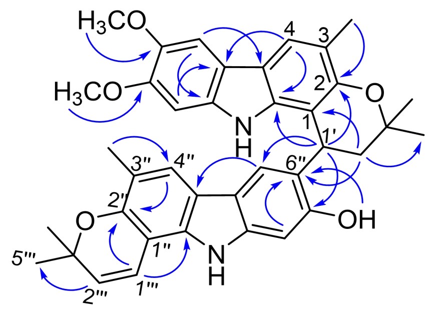


**Figure S94** Key HMBC correlations of compounds **14.**

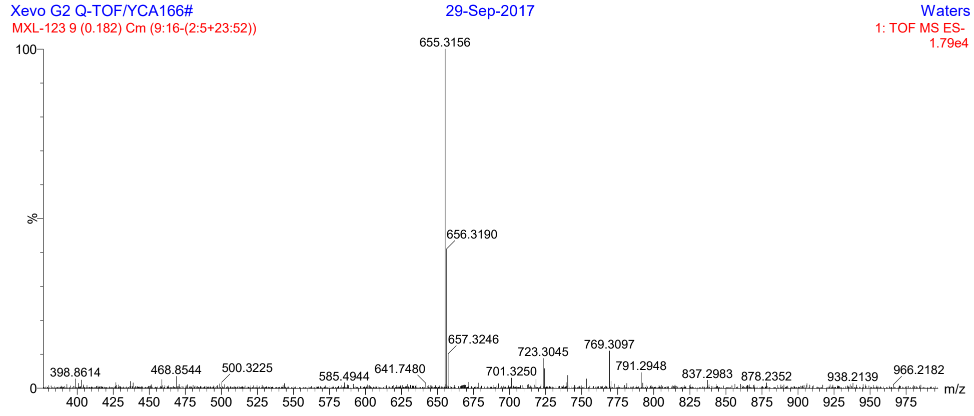


| Mass | Calc. Mass | mDa | PPM | DBE | i-FIT | Norm | Conf(%) | Formula |
|---|---|---|---|---|---|---|---|---|
| 655.3156 | 655.3172 | -1.6 | -2.4 | 22.5 | 93.9 | 1.416 | 24.27 | C42 H43 N2 O5 |

**Figure S95** HRESIMS for compound **15**.

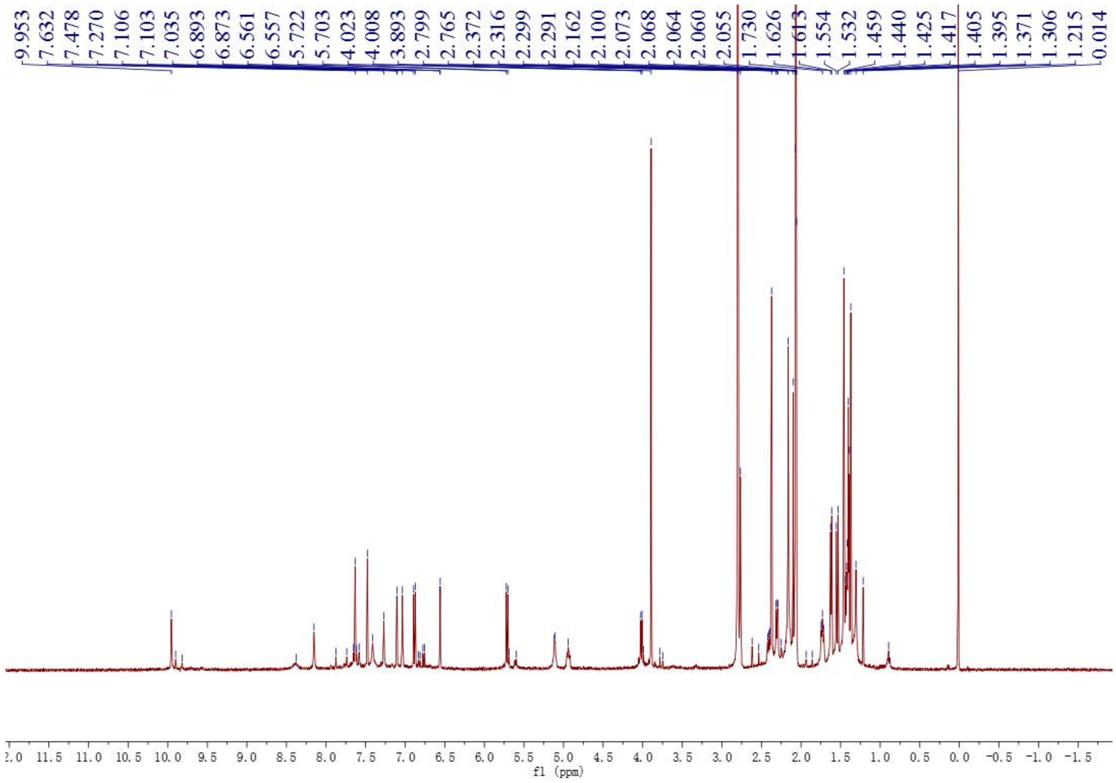


**Figure S96** $^{1}$H NMR spectrum (500 MHz, acetone-$d_6$) of compound **15**.

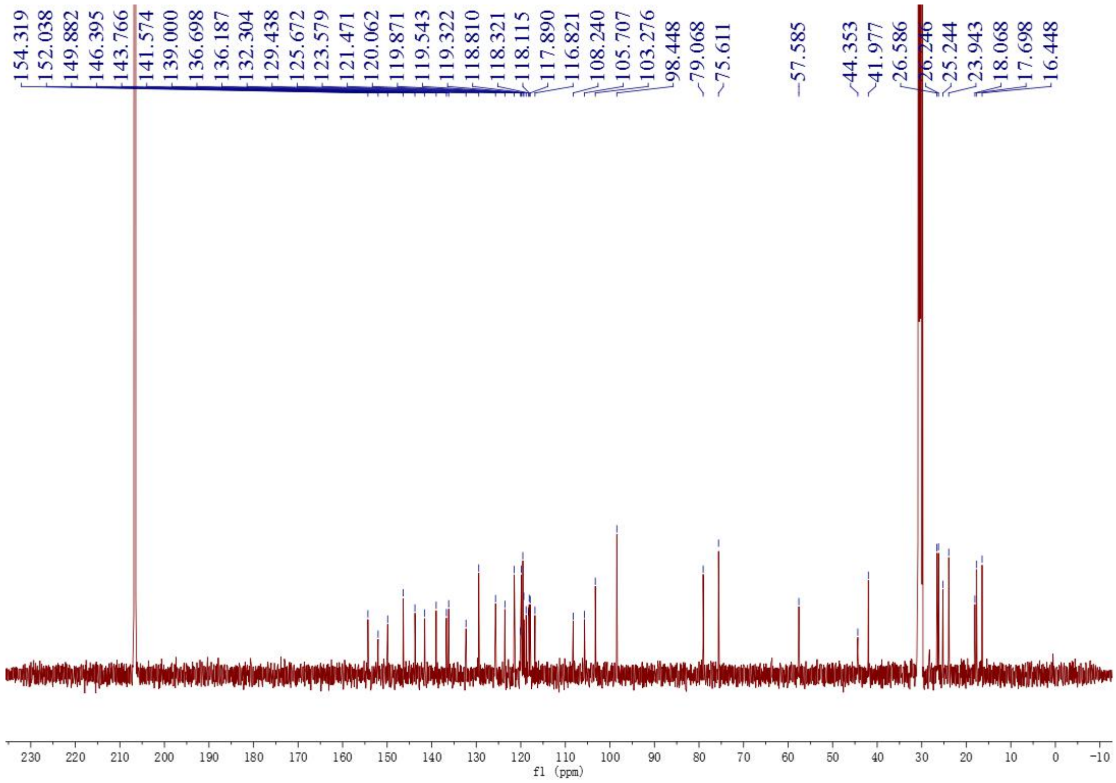


**Figure S97** $^{13}$C NMR spectrum (125 MHz, acetone-$d_6$) of compound **15**.

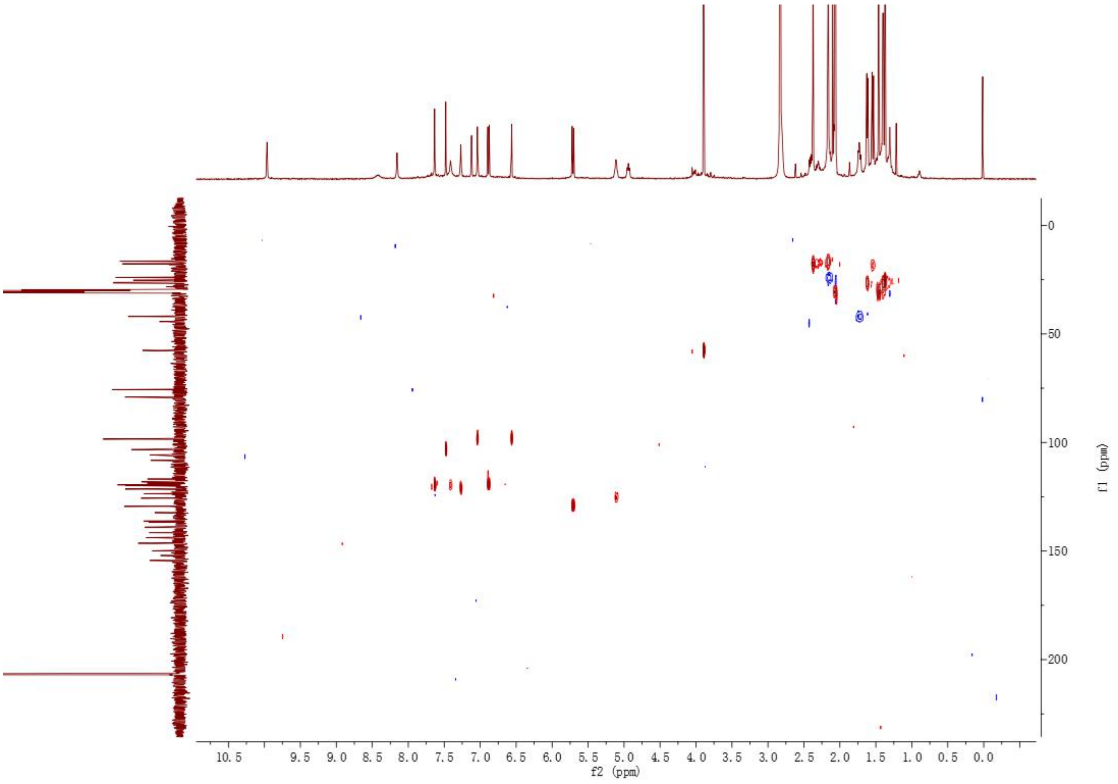


**Figure S98** HSQC spectrum of compound **15**.

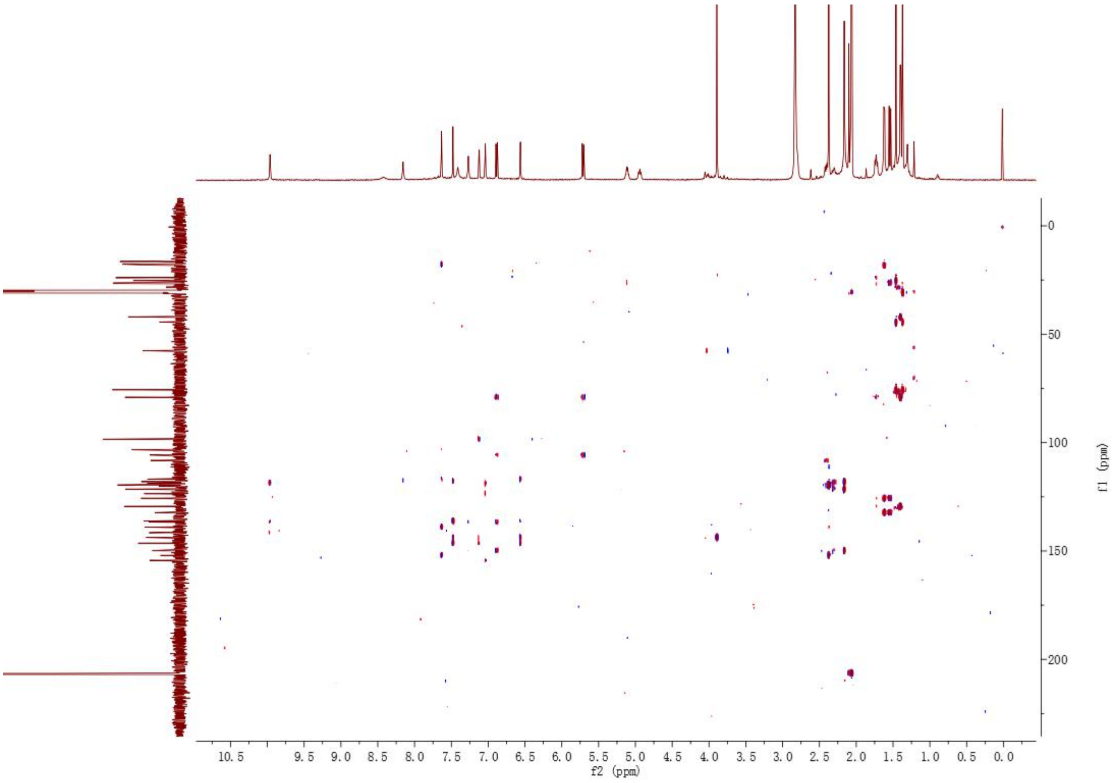


**Figure S99** HMBC spectrum of compound **15**.

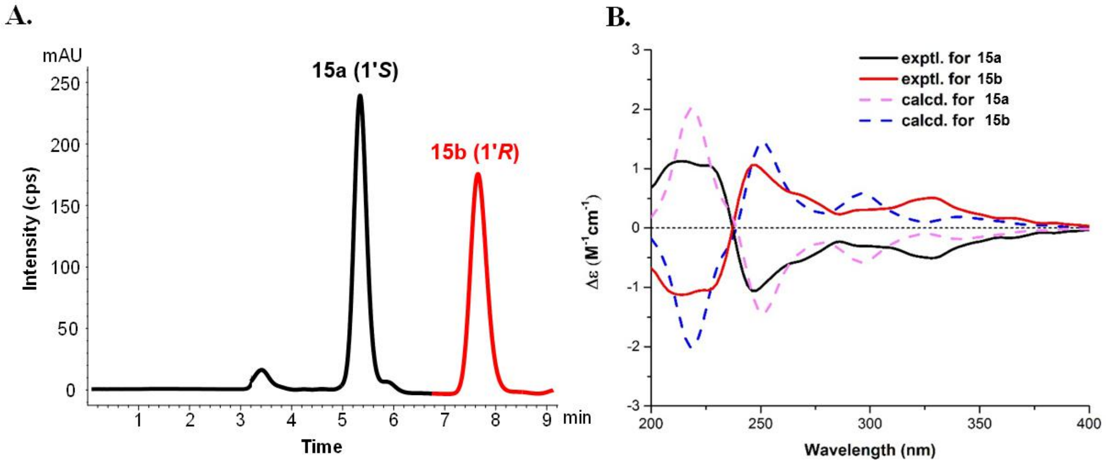


**Figure S100** The chiral HPLC separation and experimental and calculated ECD data of compounds **15a** and **15b**.

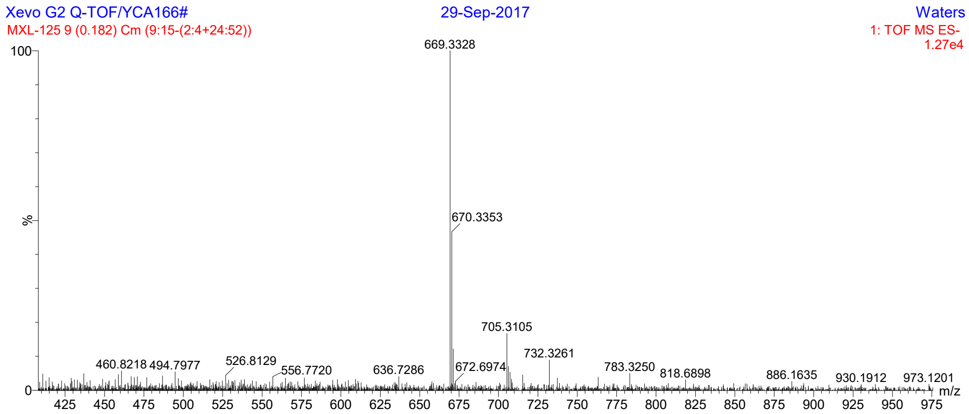


| Mass | Calc. Mass | mDa | PPM | DBE | i-FIT | Norm | Conf(%) | Formula |
|---|---|---|---|---|---|---|---|---|
| 669.3328 | 669.3328 | 0.0 | 0.0 | 22.5 | 116.9 | 0.007 | 99.26 | C43 H45 N2 O5 |

**Figure S101** HRESIMS for compound **16**.

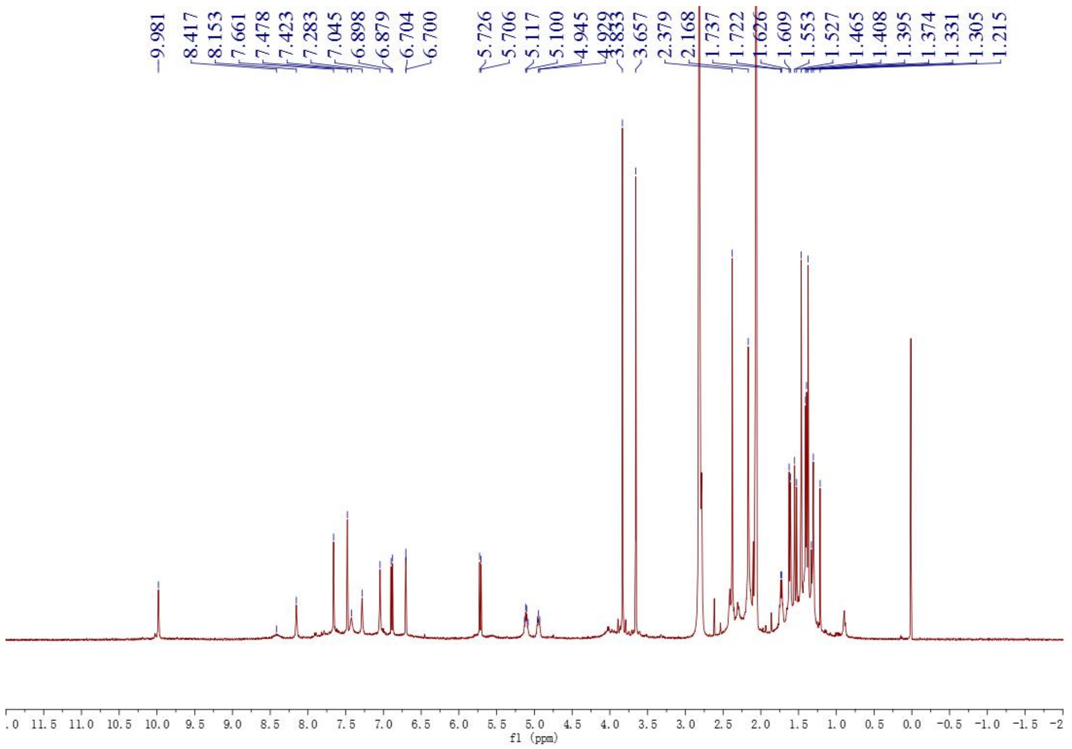

**Figure S102** $^{1}H$ NMR spectrum (500 MHz, acetone-$d_6$) of compound **16**.

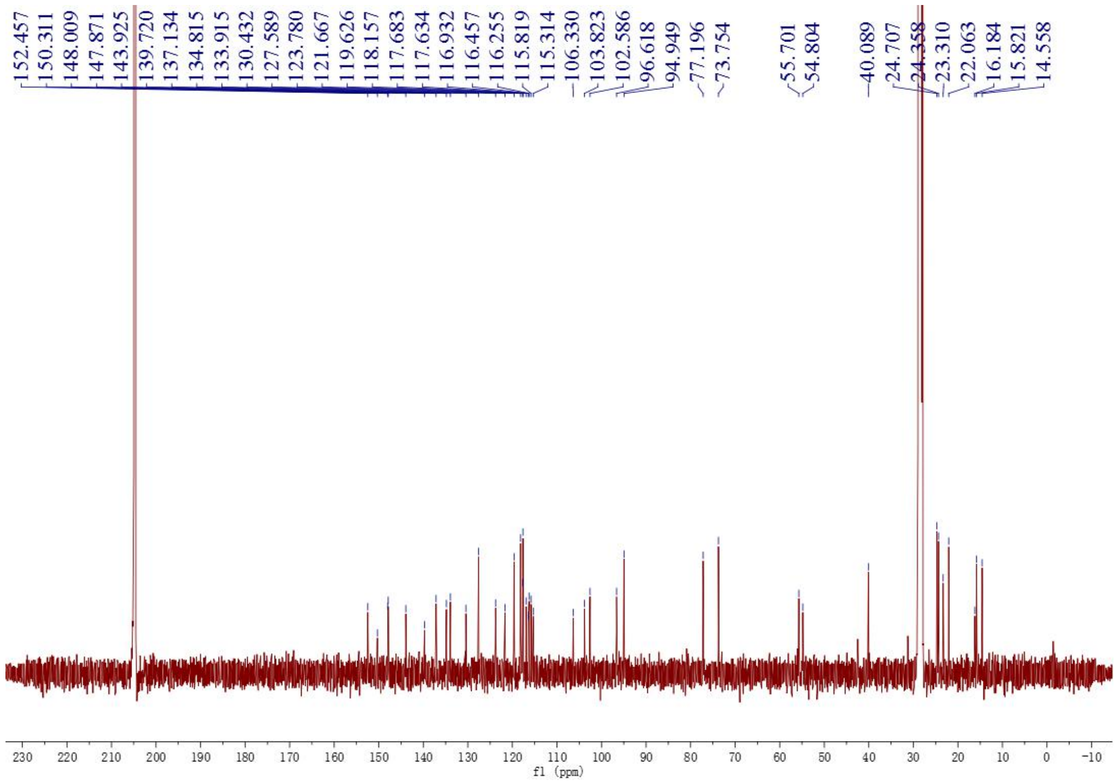

**Figure S103** $^{13}C$ NMR spectrum (125 MHz, acetone-$d_6$) of compound **16**.

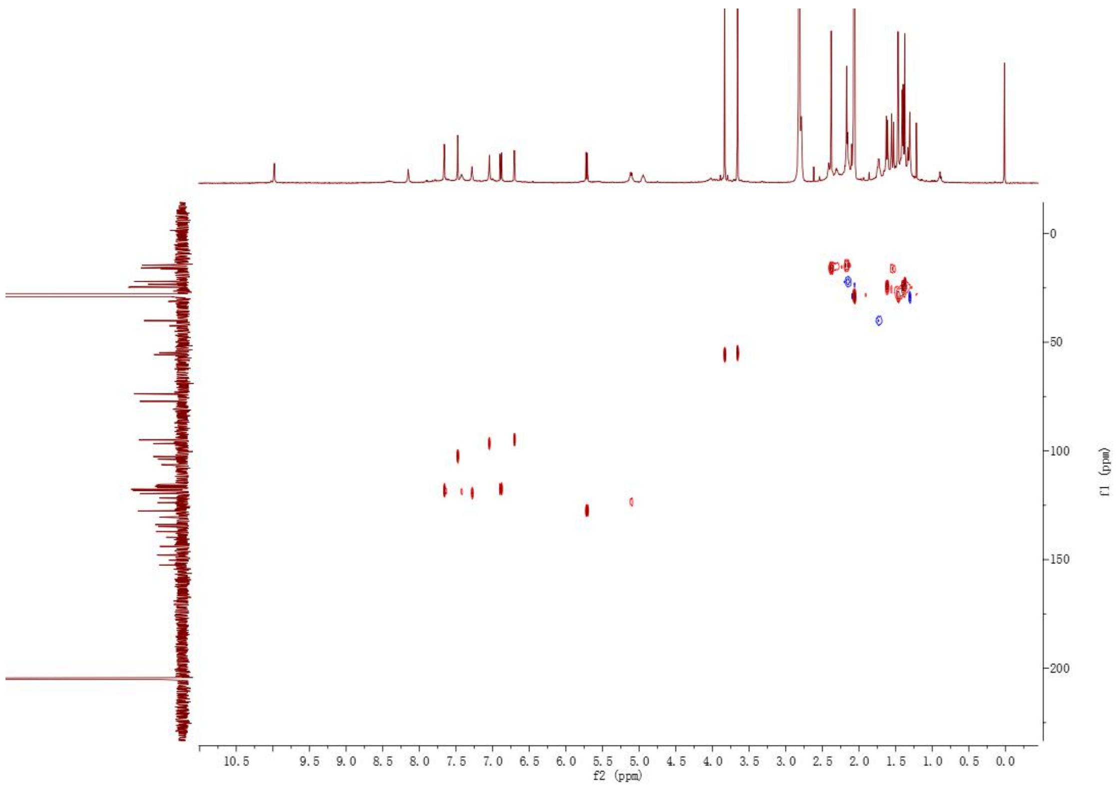


**Figure S104** HSQC spectrum of compound **16**.

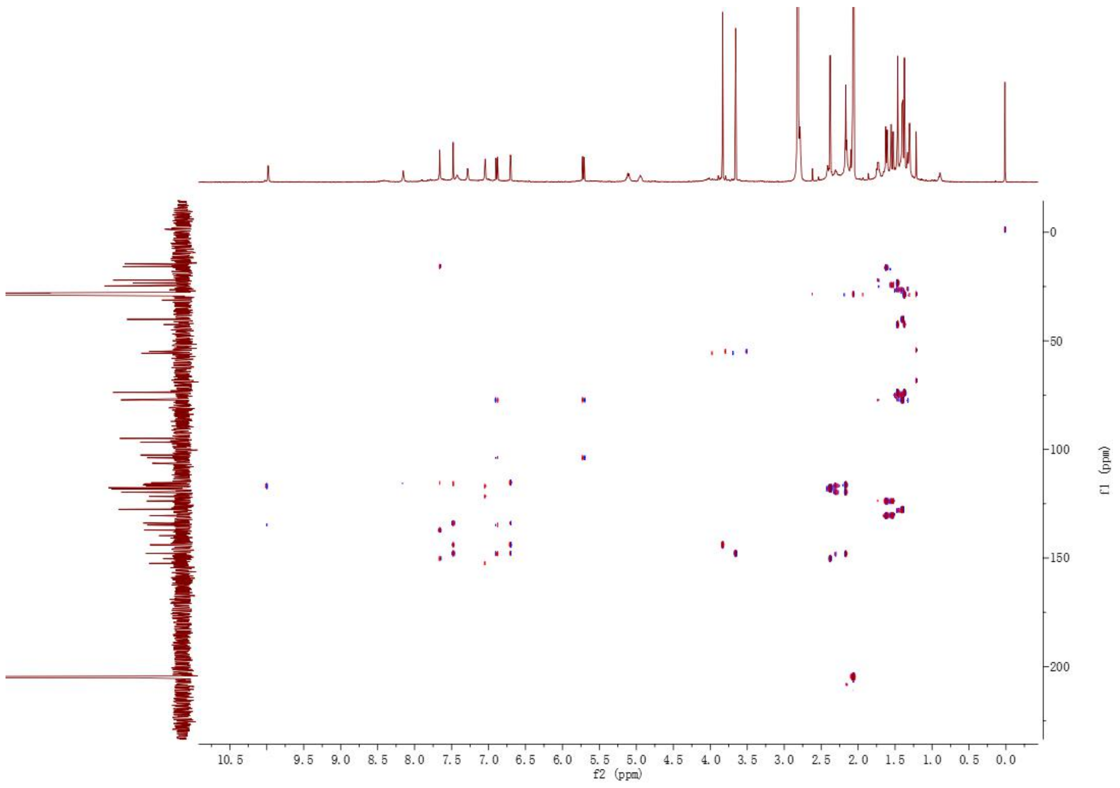


**Figure S105** HMBC spectrum of compound **16**.

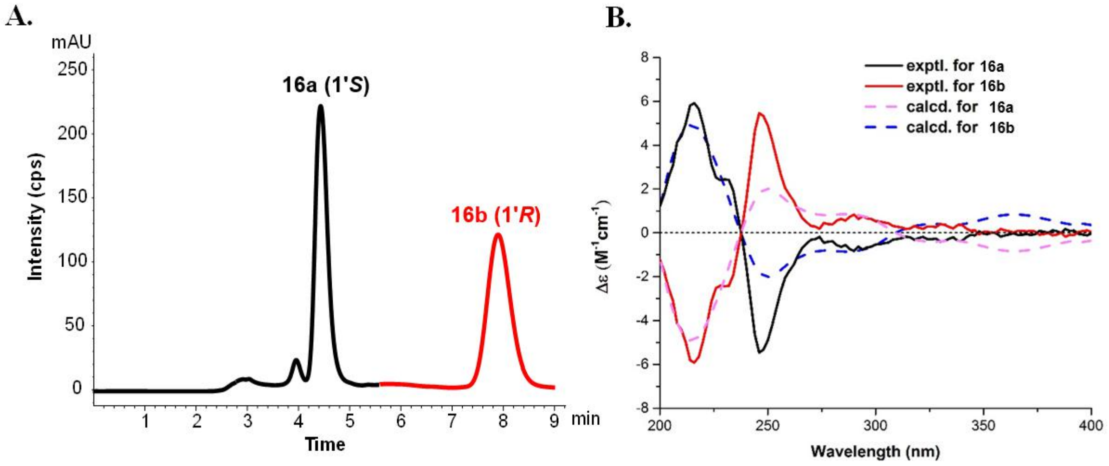


**Figure S106** The chiral HPLC separation (A) and experimental and calculated ECD data (B) of compounds **16a** and **16b**.

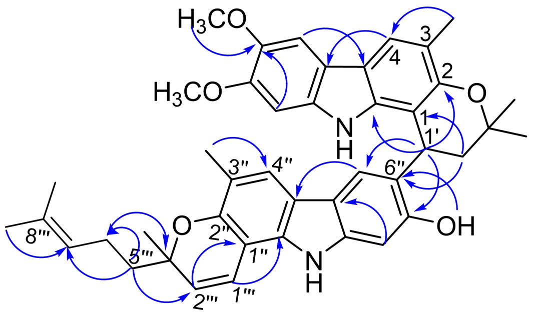


**Figure S107** Key HMBC correlations of compounds **16.**

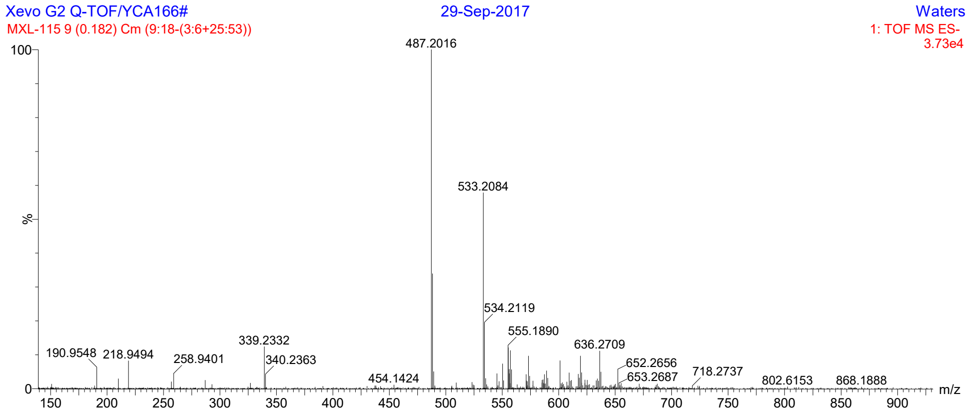


| Mass | Calc. Mass | mDa | PPM | DBE | i-FIT | Norm | Conf(%) | Formula |
|---|---|---|---|---|---|---|---|---|
| 487.2016 | 487.2022 | -0.6 | -1.2 | 20.5 | 126.9 | 0.014 | 98.59 | C32 H27 N2 O3 |

**Figure S108** HRESIMS for compound **17**.

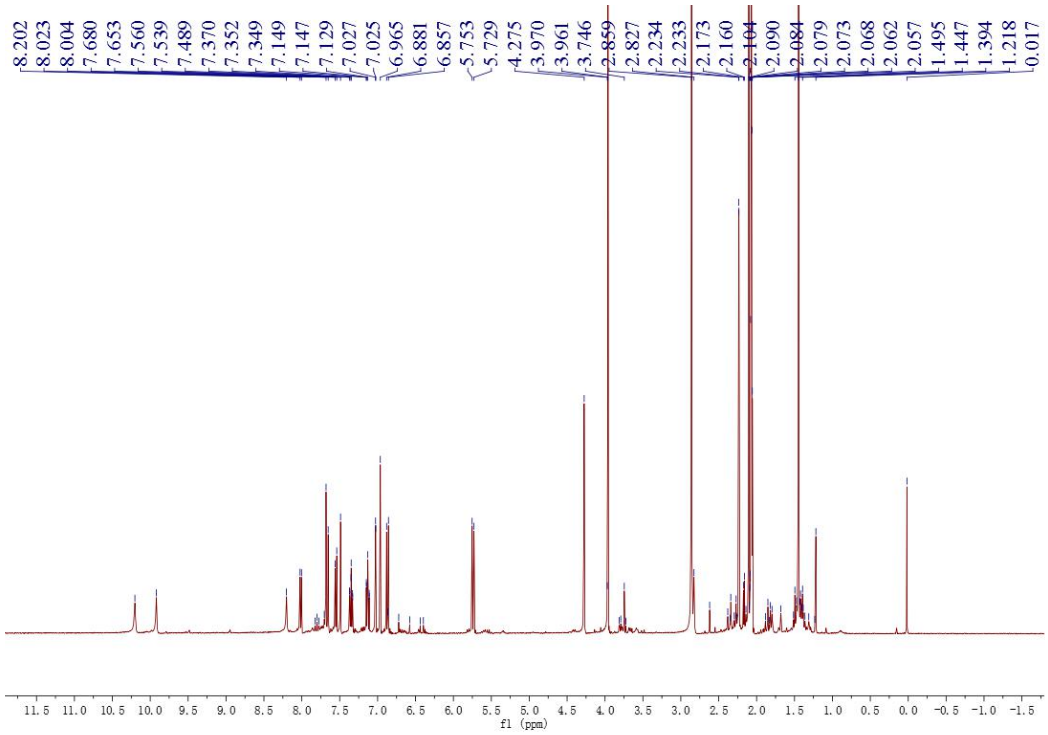


**Figure S109** $^{1}$H NMR spectrum (400 MHz, acetone-$d_6$) of compound **17**.

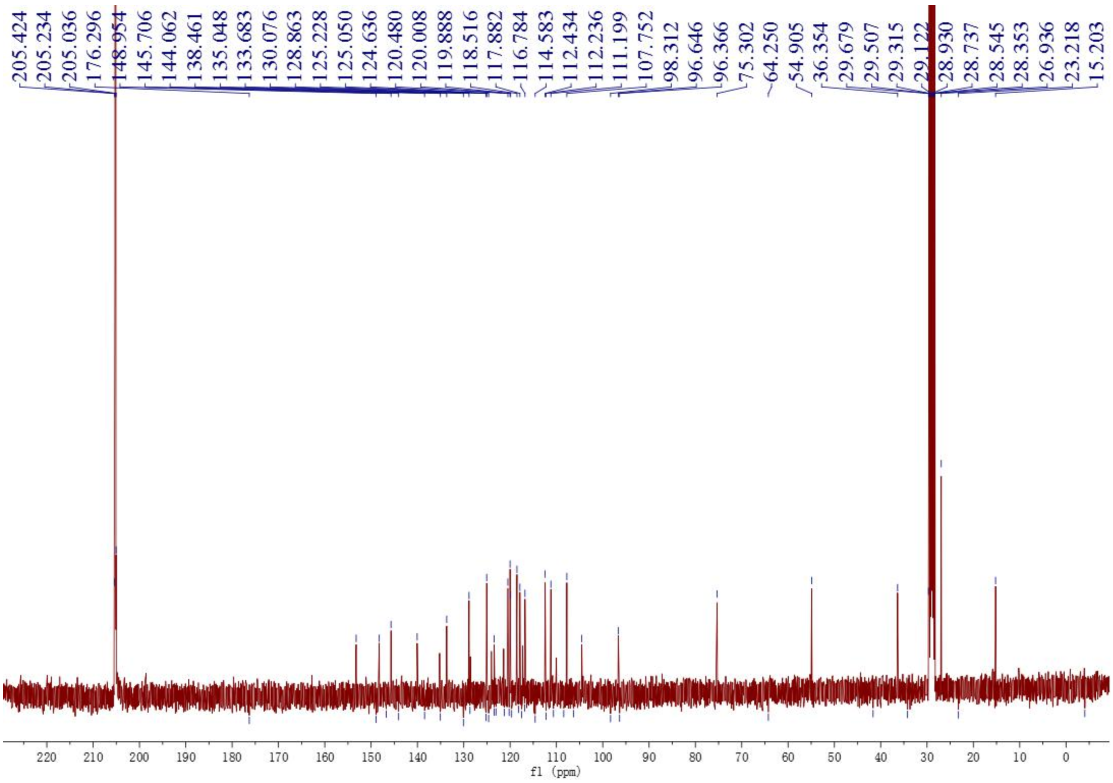


**Figure S110** $^{13}$C NMR spectrum (100 MHz, acetone-$d_6$) of compound **17**.

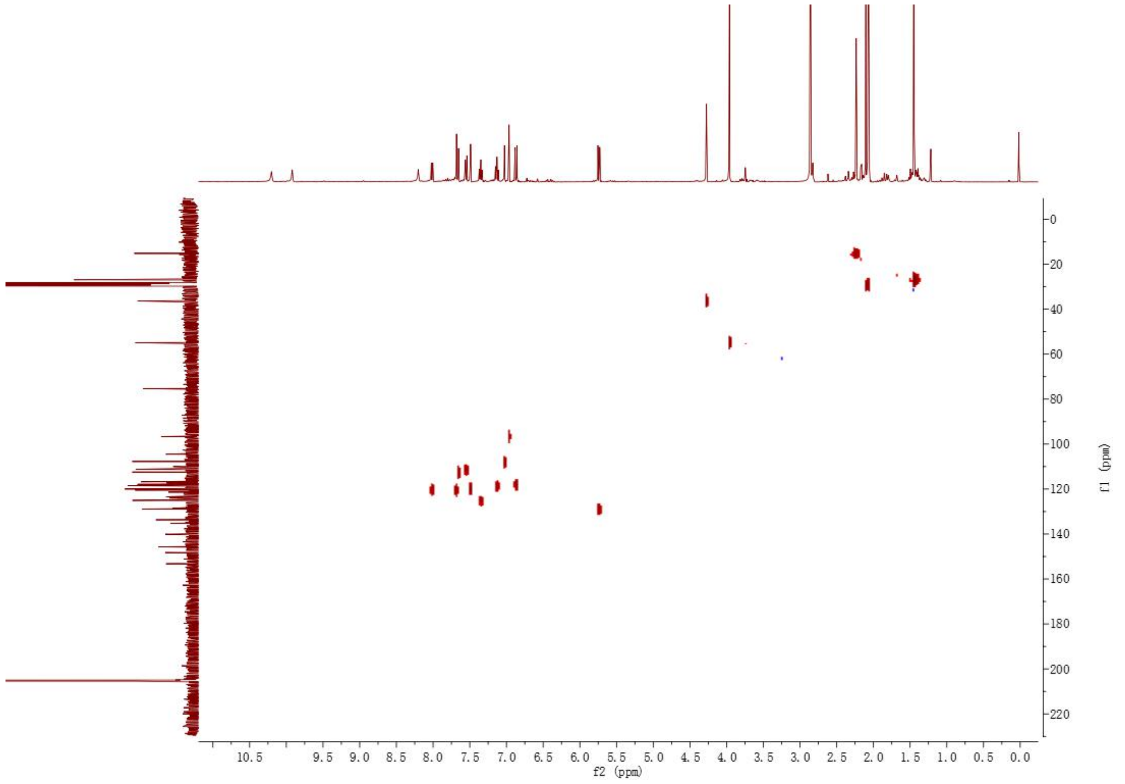


**Figure S111** HSQC spectrum of compound **17**.

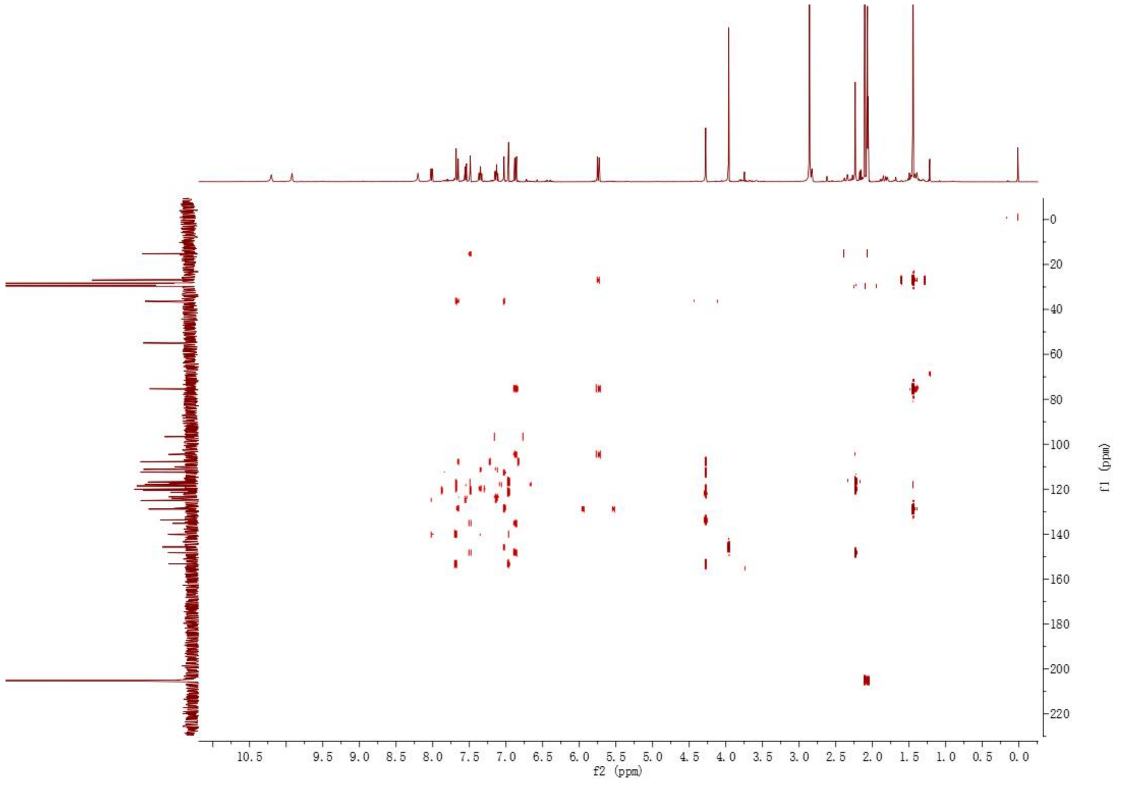


**Figure S112** HMBC spectrum of compound **17**.
